# Heterogeneous photocatalysis in flow chemical reactors

**DOI:** 10.3762/bjoc.16.125

**Published:** 2020-06-26

**Authors:** Christopher G Thomson, Ai-Lan Lee, Filipe Vilela

**Affiliations:** 1 Institute of Chemical Sciences, School of Engineering and Physical Sciences, Heriot-Watt University, Edinburgh, EH14 4AS Scotland, United Kingdom

**Keywords:** air purification, flow chemistry, heterogeneous photoredox catalysis, organic synthesis, reactor design, water purification

## Abstract

The synergy between photocatalysis and continuous flow chemical reactors has shifted the paradigms of photochemistry, opening new avenues of research with safer and scalable processes that can be readily implemented in academia and industry. Current state-of-the-art photocatalysts are homogeneous transition metal complexes that have favourable photophysical properties, wide electrochemical redox potentials, and photostability. However, these photocatalysts present serious drawbacks, such as toxicity, limited availability, and the overall cost of rare transition metal elements. This reduces their long-term viability, especially at an industrial scale. Heterogeneous photocatalysts (HPCats) are an attractive alternative, as the requirement for the separation and purification is largely removed, but typically at the cost of efficiency. Flow chemical reactors can, to a large extent, mitigate the loss in efficiency through reactor designs that enhance mass transport and irradiation. Herein, we review some important developments of heterogeneous photocatalytic materials and their application in flow reactors for sustainable organic synthesis. Further, the application of continuous flow heterogeneous photocatalysis in environmental remediation is briefly discussed to present some interesting reactor designs that could be implemented to enhance organic synthesis.

## Review

### 1 Introduction

#### 1.1 Scope of the review

This review aims to be of interest to synthetic organic chemists who are considering applying heterogeneous photocatalysis (HPC) and flow chemistry in their research, and especially those who are already involved in one of the two areas. Many independent reviews have focused on individual types of HPCat materials [1–7], and their applications in organic synthesis [8–11], solar fuel production [12–17], and environmental remediation [18–23]. Herein, we review the recent applications of HPCats in flow reactors for organic synthesis, as to the best of our knowledge, there is no review dedicated to this important and developing research area. Within the introduction, we cover the importance of the field and some important historical developments of photocatalysis (Section 1.2). We also discuss the synergy between flow chemistry and HPCats that allows chemists and engineers to achieve more efficient irradiance and mass transport, leading to a higher productivity (Section 1.3). Section 2 covers the fundamental aspects and recent developments of the major categories of HPCat materials. Section 3 details the types of flow reactors that HPCats can be applied in, with a discussion of the advantages and disadvantages to guide the reader in selecting the reactor best suited to their system. Following this, in Section 4, we review the recent applications of HPCats in flow reactors for synthetic organic chemistry through photoredox catalysis (PRC, Section 4.1) and energy transfer catalysis (EnT, Section 4.2). The final two subsections review the importance of HPCats in flow reactors for the photocatalytic remediation of wastewater (Section 4.3) and air pollution (Section 4.4), an increasingly significant area of research to prevent damage to the environment and human health. We conclude in Section 5 with a summary and our perspective on the future of HPCats in flow as a methodology for sustainable and scalable photosynthesis.

#### 1.2 Importance of the review and brief historical perspective

**Figure 1:**
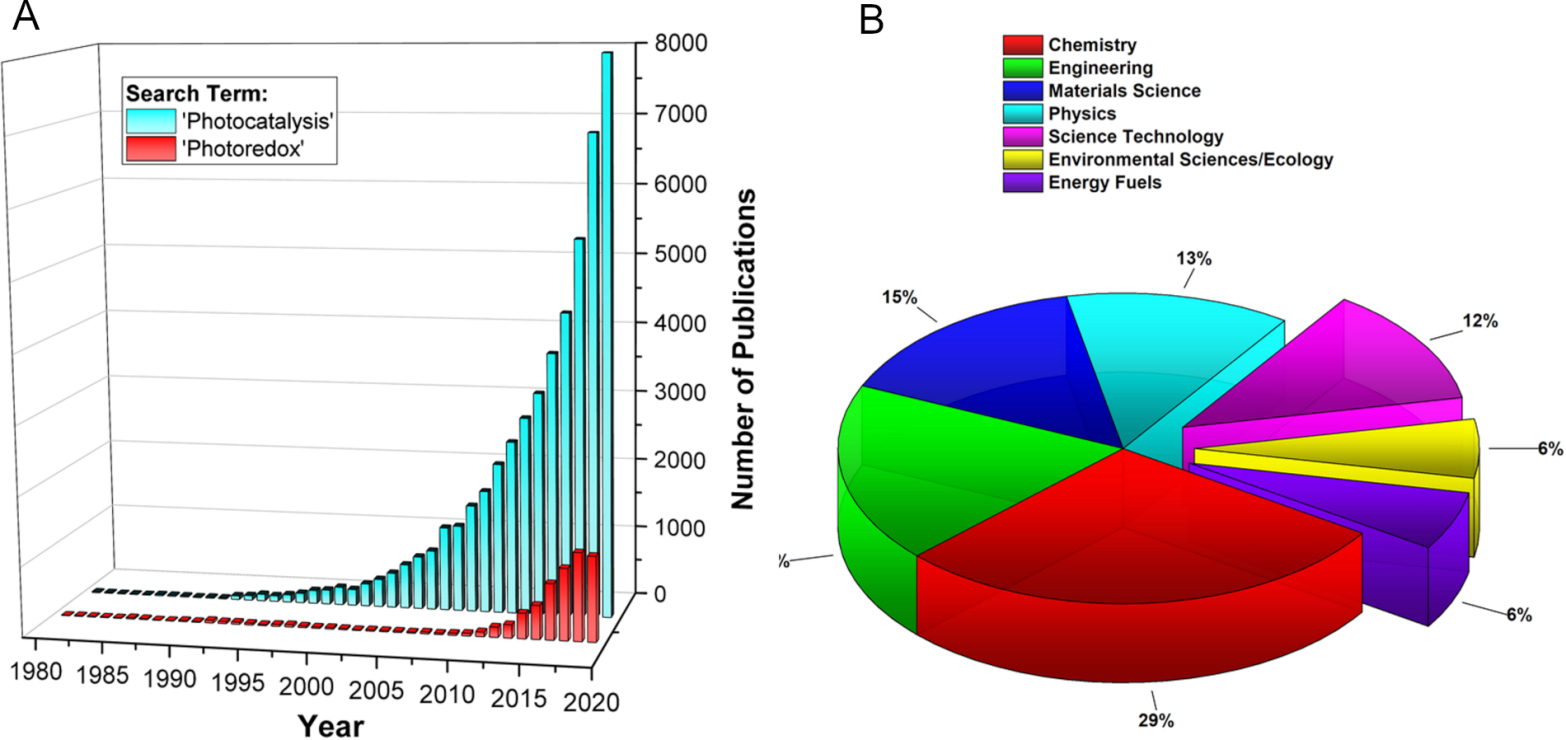
A) Bar chart of the publications per year for the topics “Photocatalysis” (49,662 instances) and “Photoredox” (6166 instances) between 1980 and 2019, queried from the Web of Science database. B) Pie chart of “Photocatalysis” publications by research areas for the top seven categories (contains 74% of the total data set) using the same data as in A.

With growing socioeconomic and political pressure to act on anthropogenic emissions and subsequent environmental damage, much of the scientific community has shifted its focus to the development of sustainable methodologies, particularly in synthetic chemistry. The prospect of utilising light as a renewable source of energy to drive chemical reactions is an appealing solution to this problem, which has led photocatalysis to become one of the most active areas of chemical research in recent years. A crude search of the Web of Science database for the term “Photocatalysis” shows the exponential growth of interest in this area since the 1990s, with almost 8000 publications on the topic in 2019 alone (Figure 1A). The credit for the development of this field is often given to MacMillan [24], Yoon [25], and Stephenson [26], whose seminal papers in 2008 and 2009 elegantly demonstrated the photocatalytic ability of ruthenium–bipyridyl complexes to drive chemical reactions with visible light through single-electron transfer processes, now referred to as visible light photoredox catalysis (PRC). A similar query on the Web of Science for the term “Photoredox” clearly shows the surge in PRC research following those reports, from 2010 onwards (Figure 1A). However, what is interesting is that PRC is still only a fraction of all photocatalysis research, which was already a vibrant field prior to 2010. This 20-year period of photocatalysis development can be largely attributed to the developing field of HPC and the work of Fujishima and Honda, who first reported the photocatalytic decomposition of water on illuminated titanium dioxide (TiO_2_) electrodes in 1972 [27]. This critical report began the field of semiconductor HPC, and in combination with the development of organic electronics, established fundamental principles that still underpin much of the cutting-edge photocatalysis research performed today.

Equally as impressive to the volume of photocatalysis publications are the broad interdisciplinary contributions to its development. Analysing the same data set of photocatalysis publications by discipline reveals that significant contributions came from engineering, materials science, and physics, in addition to chemistry (Figure 1B). This reflects the multiplex nature of photocatalysis as it requires an advanced chemical and photophysical theory to rationalise its complex mechanisms, as well as skilled engineering and reactor design to overcome the limitations of photon and mass transport.

**Figure 2:**
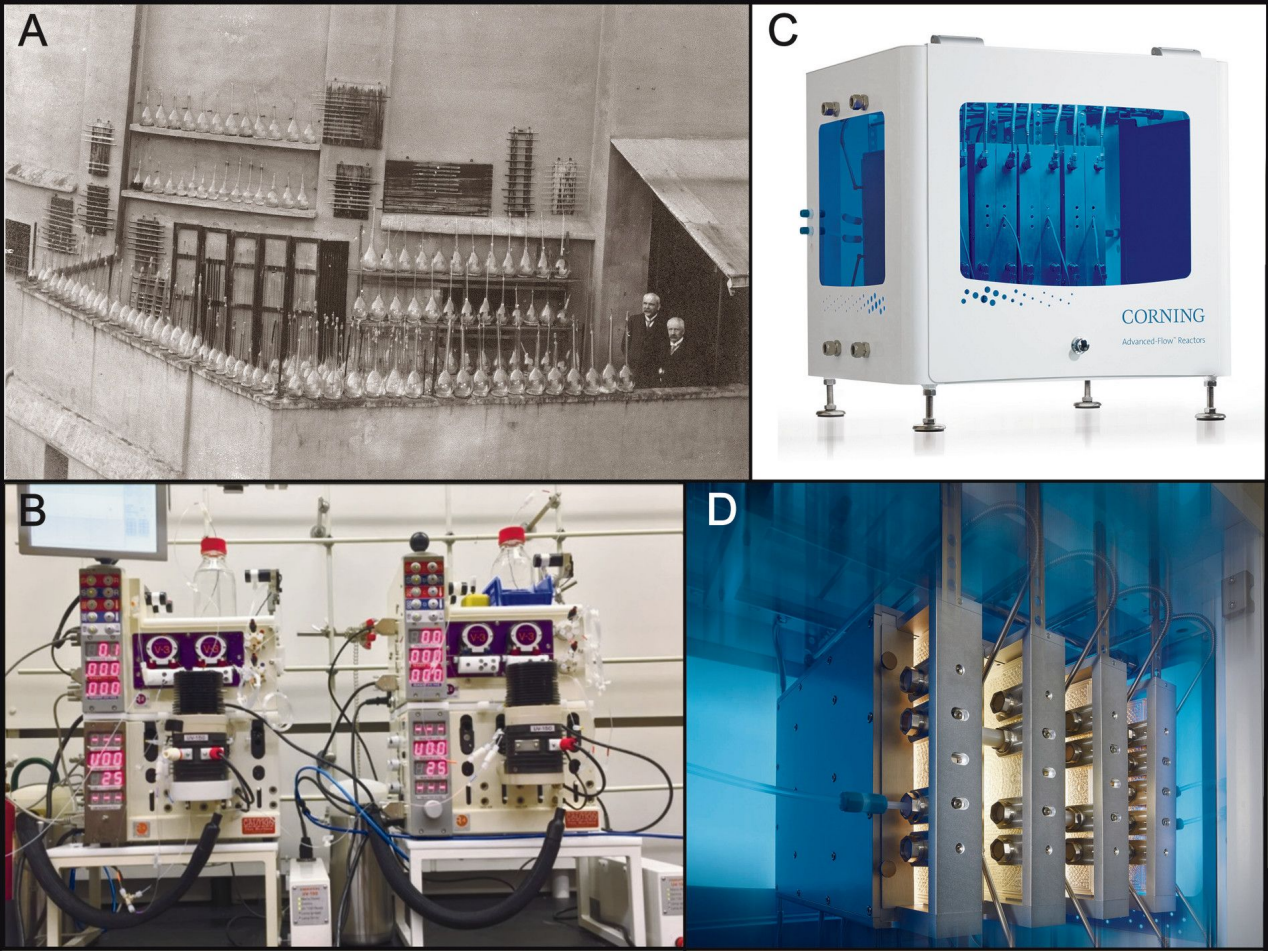
A) Professor Giacomo Ciamician and Dr. Paolo Silber on their roof laboratory at the University of Bologna, Italy, ca. 1912. Reprinted with permission, courtesy of Alma Mater Studiorum University of Bologna- University Museum System - “Giacomo Ciamician” Chemistry Collection. B) Image of two connected Vapourtec Ltd. R-Series photoreactors running in series, used by Merck for a g/h-scale photochemical synthesis. Reprinted with permission from [36], Copyright 2018 Wiley-VCH Verlag GmbH & Co. KGaA. C) Image of a Corning Inc. G3 photoreactor, capable of a 1000 tons/year production. Reprinted with permission, Copyright 2018 Corning Incorporated. D) Image of an illuminated G3 photoreactor interior featuring vertical flow panels with patented HEART design static mixing channels. Reprinted with permission, Copyright 2018 Corning Incorporated.

Photocatalysis provides a unique route to carry out complex chemical transformations under mild conditions that are often impossible with “standard” organic synthetic procedures. The earliest report of synthetic photochemistry can be traced back to Trommsdorf in 1834, who observed that crystals of santonine would shatter when exposed to sunlight [28]. Credit for the pioneering of photochemistry is usually given to Ciamician, the “grandfather of photochemistry”, who reported many interesting transformations of chemical solutions when irradiated by sunlight [29–30]. His visionary work was unfortunately limited to primitive glassware and solar irradiation, with low-yielding reactions extending for several months (Figure 2A) [29,31]. Modern day photocatalysis coupled with flow chemistry has dramatically enhanced the efficiencies and led to photochemical flow reactors being developed by organisations such as Vapourtec Ltd. (Figure 2B) and Corning Inc. (Figure 2C and D), capable of producing photochemical products on a kg/day scale [32–38].

Whilst heterogeneous catalysts are generally preferred in many industrial-scale processes [39], photocatalysis for organic synthesis is dominated by homogeneous organic dyes and phosphorescent transition metal complexes [40–42]. This is largely due to the higher efficiency of molecular photocatalysts, which disperse in solution and can be irradiated uniformly, especially in narrow flow channels [43]. The photophysical properties and surface structure of HPCats are more challenging to characterise and requires specialised techniques that are not readily available and often require multidisciplinary collaborations [44]. Additionally, the penetration of light through the bulk of the HPCat is difficult and can render large quantities of the material redundant. Overcoming these issues and producing efficient HPCats and reactors that can compete with transition metal complex photocatalysts has been described as one of the greatest challenges and opportunities in the field of photocatalysis [43,45–46]. This has led to great progress within the last decade, with new HPCat materials and reactor designs beginning to shift the photocatalysis paradigms towards HPCats because of their advantages of reduced purification and facile recycling.

#### 1.3 Benefits of heterogeneous photocatalysts in flow

In addition to separation and recycling, HPCats show advantages such as an enhanced photostability and selectivity [47–48]. A heterogeneous catalyst with a high surface area is often associated with a greater number of surface-active sites for catalysis to occur and makes morphological control critical to the catalyst efficiency. This generally holds true for HPCats, but the anisotropic surface environment of a HPCat is prone to having more trapping states for charge carriers (see Section 2), leading to more surface charge recombination events that are non-productive [49].

Often, it is only the surface and outermost layers of the HPCat that can be penetrated and activated by the incident irradiation, rendering the HPCat bulk redundant. As the mean free path of photons is proportional to their wavelength, irradiating a system with longer wavelength irradiation that still exceeds the band gap energy can enhance the depth of the photon penetration and provide more charge separation events. Upconversion photocatalysis is an emerging field of research that utilises near-infrared (NIR) radiation to penetrate deep into a reaction medium or material [50]. Multiple NIR photons are then combined through energy transfer processes by an upconversion system to produce higher-frequency, ultraviolet (UV), or visible light photons that are reemitted in close proximity to, or directly from within the HPCat solid matrix [51]. This is discussed in more detail in Section 2.5.

The “Achilles’ heel” of all heterogeneous catalysts is mass transport limitations, the circumstance in which a process becomes diffusion-limited and independent of the catalyst efficiency [43]. This is often thought of as an “engineering problem” by chemists, which will be solved through the reactor design [52], but smart design of the HPCat structure and the interfaces by the chemist can contribute, as is shown later in this review.

Fortunately, many of the complexities listed above are largely overcome through flow chemistry. The significant enhancement of the homogeneous photochemistry efficiency in flow is mostly attributed to the small reactor channel dimensions, reducing the path length of the light required to totally irradiate the reaction solution in accordance with the Beer–Lambert law (Equation 1) where the light attenuation (*A*) is the ratio of the incident light intensity (*I*_0_) to the transmitted light intensity (*I*) and is proportional to the molar attenuation coefficient of the photocatalyst (ε), its concentration (*c*), and the optical path length (*l*).

[1] $$A=\log_{10}\left(\frac{I_{0}}{I}\right)=\varepsilon \cdot c\cdot l$$

**Scheme 1:**
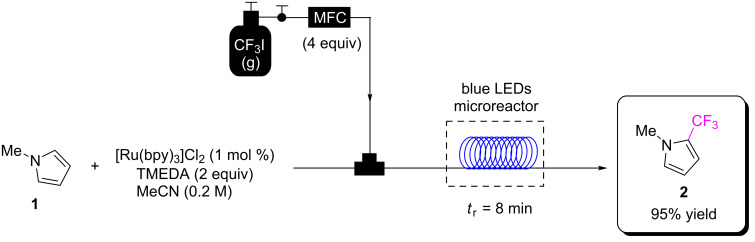
PRC trifluoromethylation of *N*-methylpyrrole (**1**) using hazardous gaseous CF_3_I safely in a flow reactor [62]. MFC = mass flow controller, *t*_r_ = reactor residence time, TMEDA = tetramethylethylenediamine.

This also applies to immobilising an HPCat in a flow reactor, which generally confines and concentrates the material within a transparent vessel with high surface-to-volume ratio, permitting a more efficient and targeted irradiation. In contrast to batch photochemistry, only the reaction medium in contact with the HPCat is irradiated, preventing overirradiation of the reaction mixture, which can lead to photodecomposition of the reactants and products [32,43]. Pumping the reaction mixture through the reactor forces the solution to flow around, or through the HPCats solid matrix, which greatly enhances mass transfer at the catalyst surface. The narrow, usually microscale, channels of flow reactors offer an unparalleled control over reaction conditions as the high surface-to-volume ratio results in an extremely efficient heat exchange. This maintains a narrow free energy profile in flow reactors and can enhance selectivity [43,53–54]. This also prevents potential hot spots forming due to a photothermal effect, which could potentially alter selectivity and lead to the loss of material [55–57]. Flow chemistry also enables chemists to safely use hazardous gaseous reagents that are otherwise avoided, whilst simultaneously increasing the gas/liquid–gas/solid interfacial surface area which enhances reactivity [58–61]. This was highlighted by Noël and co-workers, who demonstrated the safe use of gaseous CF_3_I as a reagent for the continuous flow PRC trifluoromethylation of 5-membered heterocycles, such as *N-*methylpyrrole (**1**, Scheme 1), with a high conversion and selectivity for the monofunctionalised product **2** [62]. The system achieved full conversion for a variety of aromatic heterocycle substrates within 8–20 min, transformations that would require days in batch protocols (Scheme 1) [62].

Flow systems can be pressurised easily with back pressure regulators, enhancing the solubility of gaseous reagents in the reaction solvents [36,63]. Flow processes are also commonly monitored with in-line and on-line spectroscopies, such as UV–vis, FTIR, mass spectrometry, and NMR [63–68]. These systems can be automated to ensure consistency and to remove the need for laborious manual sampling. In-line and on-line monitoring is well aligned with automated synthesis and high-throughput reaction screening, both of which are progressive areas of chemical research [69–73].

### 2 Heterogeneous photocatalysts

Probably the first report of HPC was published by Renz in 1921, who observed the partial reduction of titania (TiO_2_) in the presence of glycerol when irradiated with sunlight [74]. The use of titania as a pigment had been practiced for centuries, and the observation that TiO_2_-based surface coatings exposed to sunlight irradiation would “chalk” (the formation of a loose white powder on the paint surface) had been recognised as the decomposition of organic components in the coating, leaving the TiO_2_ powder exposed [75]. In 1938, Goodeve and Kitchener used this knowledge to perform the first study on the photodegradation of organic dyes with TiO_2_ powder [76]. A series of subsequent reports that were historically relevant to developing TiO_2_- and semiconductor-based photocatalysis followed, which can be explored in a review by Fujishima [75]. It was not until the landmark paper by Honda and Fujishima in 1972 that the field of HPC and photoelectochemistry flourished [27]. This paper reported what is now known as the Honda–Fujishima effect in which TiO_2_ was found to split water to oxygen and hydrogen gas under strong UV irradiation. This gained significant attention in the wider scientific community for the potential generation of solar fuels [27,75] and formed the foundation of modern heterogeneous photocatalysis.

With this brief historical perspective in mind, it is perhaps not surprising that TiO_2_ as a material for photocatalysis is incredibly well studied and characterised. TiO_2_ has excellent physicochemical properties, good biocompatibility, is abundant in nature, and subsequently inexpensive. For these reasons, the application of TiO_2_ in photocatalysis is still substantial and has featured in approximately 50% of all photocatalysis publications from 2015–2019, by analysis of the same data set presented in Figure 2A. In this section, we will cover the fundamentals and recent advances in material design of the main categories of HPCats, beginning with TiO_2_ as a model system for metal oxide and inorganic semiconductor HPCats.

#### 2.1 Inorganic semiconductor photocatalysis

**Figure 3:**
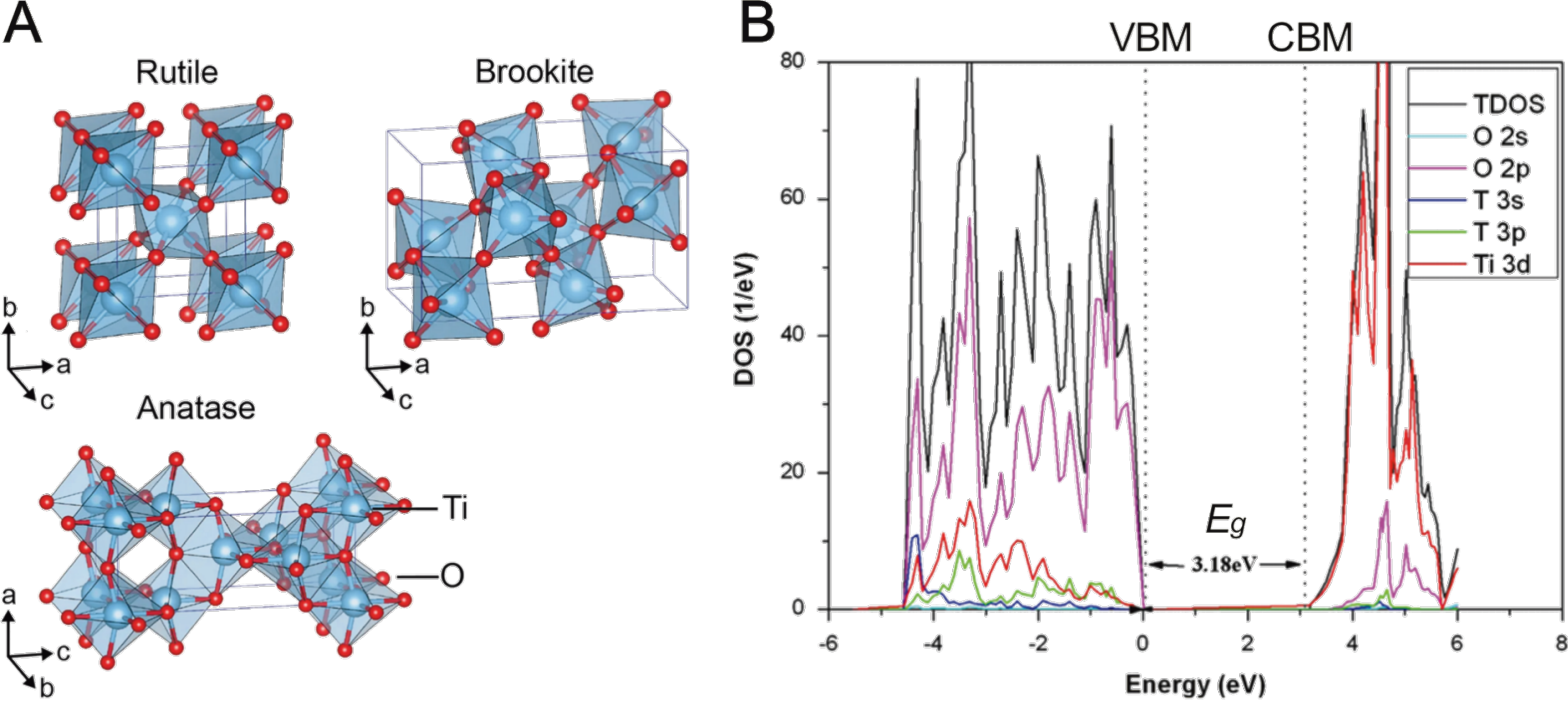
A) Unit cells of the three most common crystal structures of TiO_2_: rutile, brookite, and anatase. Reprinted from [83] (copyright held by the authors), published by Springer Nature under a Creative Commons Attribution 4.0 International License http://creativecommons.org/licenses/by/4.0/. B) Theoretical density of the states diagram for anatase TiO_2_, with dashed lines indicating the conduction band minimum (CBM) and the valence band maximum (VBM), which yields the band gap energy (*E*_g_) of 3.18 eV. Adapted from [78].

Semiconductors are defined as materials that have a conductivity between that of an insulator and a conductor [77]. The origin of conductivity is based on the quantum states of electrons in a solid, known as the band theory. Bands are groups of electronic quantum states that arise from the atomic or molecular orbitals of a solid material, leading to continuums of indiscrete energy states [77]. TiO_2_ is a crystalline material with three common lattice arrangements named anatase, brookite, and rutile (Figure 3A). The different lattice structures influence the atomic orbital environments and energy, which alters the photophysical and charge transport properties of the material, which has been studied in great detail [78–82]. The titanium and oxygen atomic orbitals generate a filled band, the valence band (VB), of mainly oxygen 2p electron density and an empty band of unoccupied Ti 3d states, the conduction band (CB), separated by a gap of energy potentials with no quantum states, the band gap (*E*_g_), corresponding to 3.18 eV for anatase TiO_2_ (Figure 3B) [78–79].

The semiconductor *E*_g_ defines the minimum frequency of photons required to excite an electron from the valence band to the conduction band, the critical primary event required for photocatalysis to occur. The band structure can be engineered by controlling the polymorphism, morphology, and size (e.g., TiO_2_ nanoparticles) or by introducing impurities into the crystal lattice, otherwise known as doping [44,84–86]. Semiconductor doping adds additional filled or vacant quantum states to the band structure that lie within the band gap, reducing the *E*_g_ and typically resulting in a bathochromic shift in absorption. Synthetic TiO_2_ commonly has oxygen atom vacancy defects, especially at its surface, resulting in Ti^3+^ ions that produce quantum states close to the conduction band minimum. These states lower the *E*_g_, enabling visible light absorption and producing reactive centres on the catalyst surface, which has been synthetically controlled to improve the photocatalytic CO_2_ reduction performance by Zhang and co-workers using plasma treatment [87].

**Figure 4:**
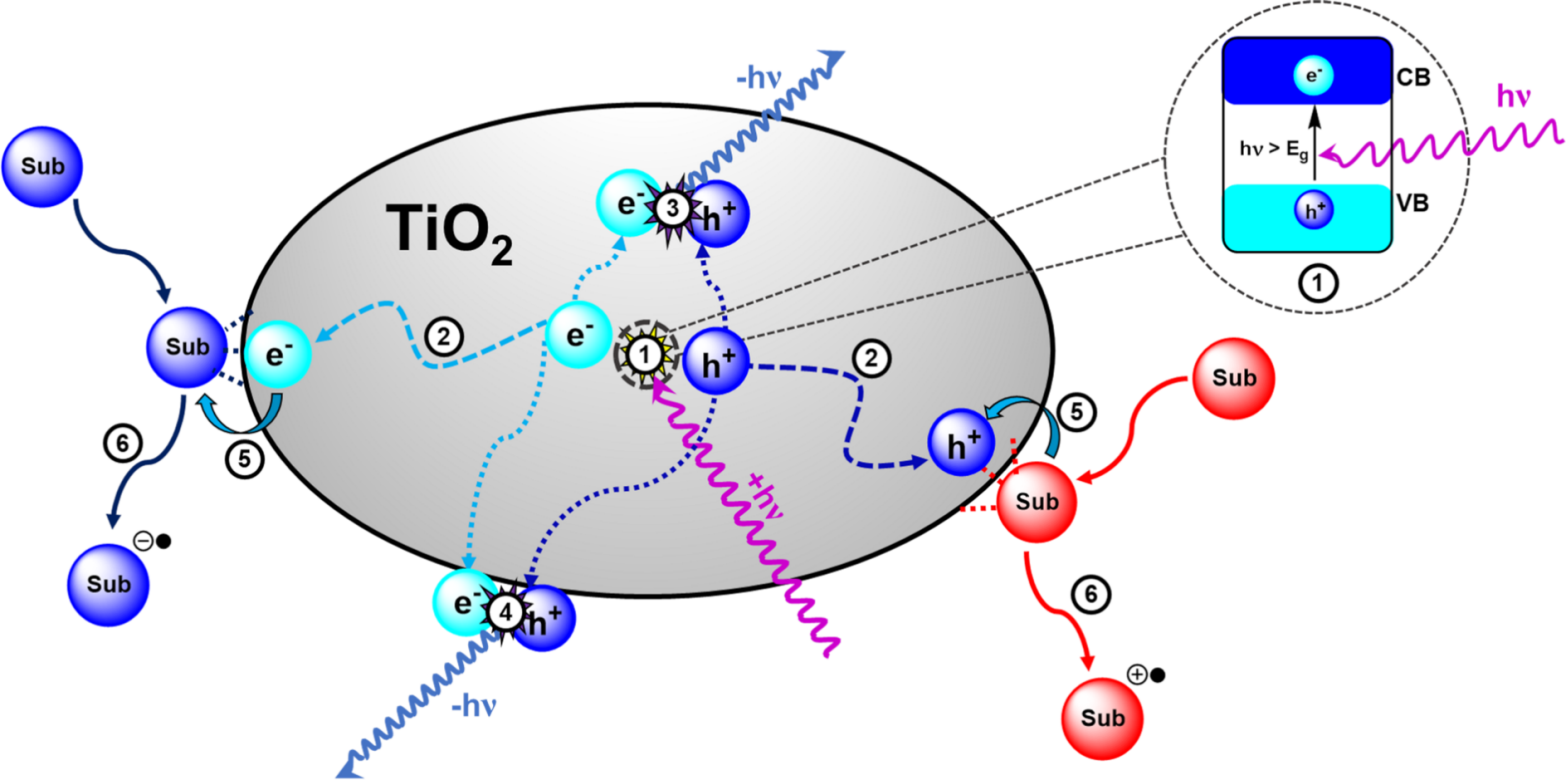
Illustration of the key semiconductor photocatalysis events: 1) A photon with a frequency exceeding the band gap energy (*E*_g_) excites an electron from the VB to the CB, producing an exciton (fs). 2) The exciton dissociates into free charge carriers (e^−^_CB_ and h^+^_VB_) and migrate to the semiconductor surface. 3) and 4) e^−^_CB_ and h^+^_VB_ are immobilised by trapping states within the bulk (3) or at the surface (4, 100 ps–250 ns) and recombine radiatively (−*h*ν, 1 ps–10 μs) or non-radiatively (10–100 ns). 5) The substrate (Sub) species transfer and adsorb to the surface where they are oxidised or reduced by the e^−^_CB_ and h^+^_VB_, respectively (1 ns–ms). 6) The oxidised/reduced substrates desorb from the surface and transfer into the bulk solution to undergo further chemical processes (>ms). Approximate timescales were reported by Bahnemann and co-workers (fs = 10^−15^, ps = 10^−12^, ns = 10^−9^, μs = 10^−6^, ms = 10^−3^ s) [88].

The general process of photocatalysis in metal oxide semiconductors is illustrated in Figure 4 and is as follows: the photoexcitation of an electron across the band gap generates an energetic electron in the CB (e^−^_CB_) and leaves behind a positively charged electron vacancy in the VB, referred to as a hole (h^+^_VB_). These electron/hole pairs are the charge carriers that allow the oxidation and reduction of substrates in photocatalysis. Initially, the electron/hole pair is in a bound state, held together by electrostatic attraction and treated as a single neutral quasiparticle called an exciton. A quasiparticle is a mathematical solution applied to microscopically complicated systems which account for experimentally observed phenomena, such as the reduced velocity of particles within a solid. By treating them as pseudo-particles that have the same charge but increased mass, this better reflects experimental observations and permits better system modelling [82].

In materials with high dielectric constants, the charges are shielded from each other, and the exciton readily dissociates into free charge carriers. Following the charge separation, the charge carries migrate to a reactive site on the surface of the material to oxidise or reduce a substrate, respective of their charge. A reactive site is generally a point where a substrate has adsorbed to the HPCat surface and is within the proximity required for an electron transfer or energy transfer process to occur. Substrate reduction and oxidation by an excited electron and hole, respectively, returns the semiconductor to its initial state and activates the substrate to further reactivity at the surface or in the bulk solution, completing the photocatalyst cycle.

Charge carriers must overcome competing processes that result in the immobilisation and recombination of charge carriers. The photogenerated electron/hole pair will spontaneously undergo bulk or surface recombination if they cannot efficiently separate, which is influenced by the dielectric properties of the material and delocalisation of the quantum states. Highly localised quantum states in the band gap, such as those introduced by dopants and defects in the bulk lattice, are known as trap states and can immobilise charge carriers, increasing the probability of charge recombinations [89].

Serpone et al. found that 85% of the photogeneration events resulted in a recombination after 10 ns of illuminating TiO_2_ particles [90]. This implies that higher irradiation intensity may hinder photocatalysis by increasing the charge carrier density and subsequently the rate of recombination [91].

**Figure 5:**
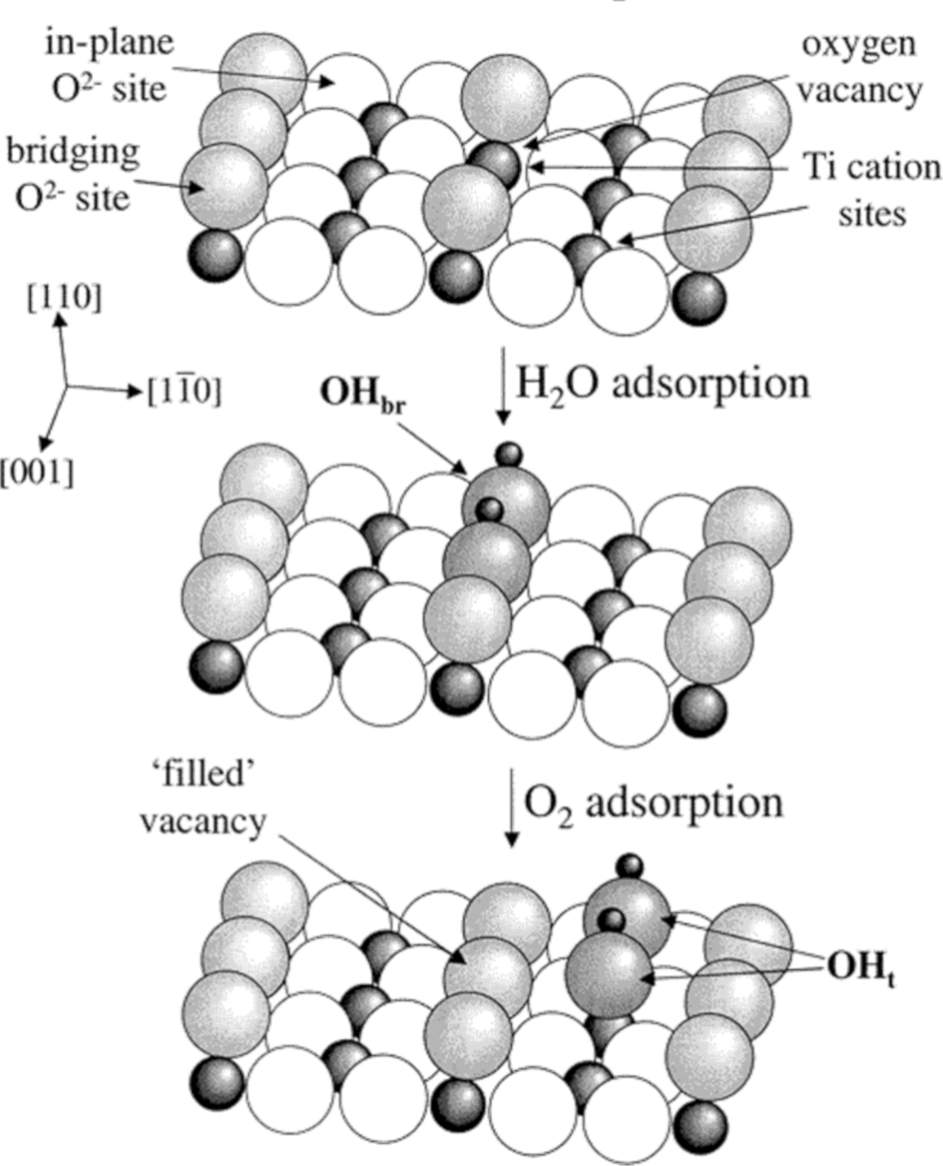
Photocatalytic splitting of water by oxygen vacancies on a TiO_2_(110) surface. Reprinted with permission from [92]. Copyright 2003 American Chemical Society.

The abrupt termination of an ordered lattice continuity at a surface leads to chemical and structural differences from the bulk material. TiO_2_, for example, has surface hydroxides, oxygen anions, and Ti^3+^ cations, which produce high energy states from incomplete bonding. This energy is alleviated though surface relaxation, a process in which the surface changes its structure to reduce its energy, or by the adsorption of substrates to fill and stabilise vacancies. Water molecules adsorb to oxygen vacancies on the TiO_2_ surface and undergo a 2-electron reduction and proton transfer to an adjacent oxide atom, forming two bridging-hydroxyls. Subsequently, an adsorbed O_2_ molecule is reduced by the bridging hydroxyls to form two terminal hydroxides and fill the initial oxygen surface vacancy (Figure 5) [92].

Organic molecules generally weakly associate with the surface via physisorption. However, functionalised molecules and ions can strongly bind through chemisorption by covalent bonds, hydrogen bonding, or electrostatic attraction and have been shown to influence the photophysical properties of TiO_2_, generally causing a bathochromic shift of the absorption spectrum through direct VB/adsorbate electron transfer transitions [93–94]. HPCats modified with coordinating transition metal complexes also usually display significant changes to their absorption spectrum through the introduction of metal-to-ligand, ligand-to-metal, ligand-to-ligand, and metal-to-metal charge–transfer transitions [95]. These transitions are commonly observed in metal–organic framework (MOF) HPCats, discussed more in Section 2.5. Dye-sensitised semiconductor photocatalysts have organic photosensitiser molecules immobilised to their surface. This strategy is typically used to activate a wide-band gap semiconductor towards visible light excitation [95]. The photosensitiser can inject electrons to the semiconductor conduction band via direct HOMO–CB transition (type II excitation) or indirectly by exciting the photosensitiser HOMO–LUMO transition, followed by a LUMO–CB charge transfer (type I excitation). The direct VB-to-LUMO or VB-to-ligand transition is also enabled and produces a hypsochromic shift in the HPCat absorption spectrum. An excellent review of coordination chemistry controlled design of heterogeneous photocatalysis has been published by Xiong and co-workers, and we recommend exploring the article for further details [95].

**Figure 6:**
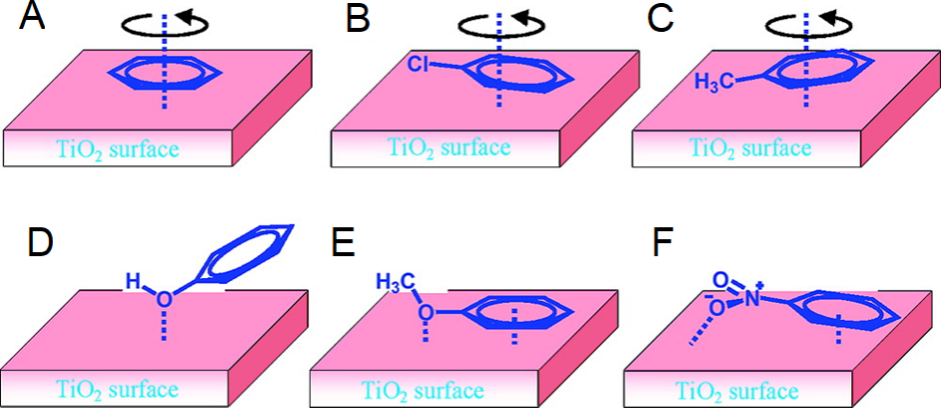
Proposed adsorption modes of A) benzene, B) chlorobenzene, C) toluene, D) phenol, E) anisole, and F) nitrobenzene on a TiO_2_ surface. Reprinted with permission from [96]. Copyright 2011 American Chemical Society.

Molecules with functional groups such as carboxylic acid present complex surface topologies from bridging and bidentate binding modes [91]. Yoshida and co-workers studied the adsorption of benzene derivatives on TiO_2_ surfaces by solid-state NMR and revealed the chemical shifts varied for different atoms, indicating the multiple orientations of related adsorbates to the surface (Figure 6) [96].

**Figure 7:**
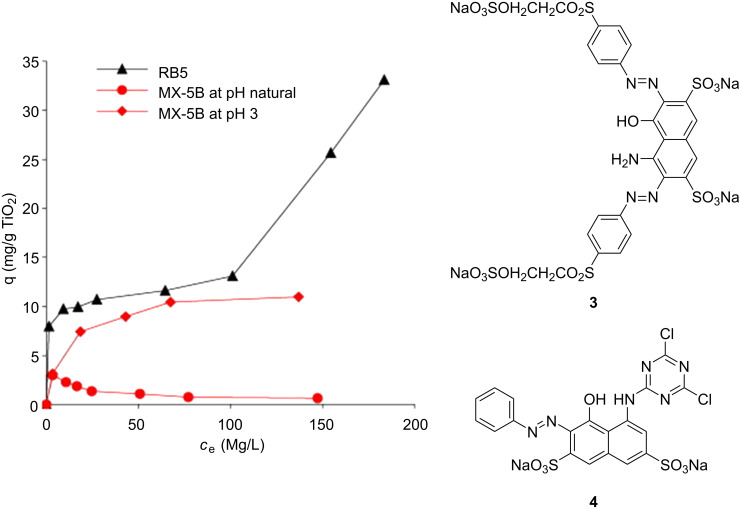
Structures of the sulfonate-containing organic dyes RB5 (**3**) and MX-5B (**4**) and the adsorption isotherms of the dyes on a TiO_2_ surface at varied pH. The additional adsorption of **3** is due to the formation of a bilayer. Reprinted from [98].

Due to the amphoteric nature of metal oxide surfaces, the pH value of the system has a significant influence on photocatalysis by altering the Coulombic forces between the surface and substrate. The pH value at which the concentration of surface charge centers is equal is termed the point of zero charge (PZC), which is 5.80 for TiO_2_, approximately 8.5 for most metal oxides, and approximately 2.0 for metal sulphides [97]. Guillard and co-workers studied the photocatalytic degradation of basic organic dyes containing sulfonate groups, remazol black 5 (**3**, Figure 7) and procion red MX-5B (**4**, Figure 7), and showed a significant enhancement of **4** adsorption when the pH value was reduced to 3 (Figure 7) [98]. The authors stated that this reflected a higher positive charge density on the TiO_2_ surface at a lower pH value, which favours the adsorption of the dye via its negatively charged sulfonate groups. However, at a natural pH value, the increasing dye concentration gradually raises the pH value of the system and negatively charges the TiO_2_ surface, leading to an electrostatic repulsion that prevents the dye adsorption.

#### 2.2 Organic semiconductor photocatalysis

Polyaniline was the first conductive polymer reported by Henry Letheby in 1862 [99–100], but the potential of organic electronics was not realised until the 1960s, and the seminal work of Heeger, MacDiarmid, and Shirakawa, leading to their shared award of the 2000 Chemistry Nobel Prize for *“*the discovery and development of conductive polymers*”* [101]. For the following 60 years, and still to this day, organic electronics is a vibrant field of research within materials chemistry and physics as a potential source of sustainable photovoltaics [102], flexible electronics [103], improved organic light emitting diodes (OLEDs) [104], organic field effect transistors (OFETs) [105–106], and more recently, photocatalysts [3,5,10,15]. These materials have semiconducting properties due to extended conjugation producing a continuum of bonding and antibonding molecular orbital states which form band structures, analogous with inorganic semiconductors. Hence, the mechanisms of organic semiconductor photocatalysis and the photogeneration of charge carriers is identical to those discussed in the previous section and illustrated in Figure 4. The differences between inorganic and organic semiconductors are primarily the transport of charge within the material, which arise from the drastically different properties and various degrees of crystallinity of organic semiconductors, which we will now briefly discuss.

In amorphous polymer chains, charge transport is limited to intraplanar transport along the conjugated network. In crystalline and graphitic organic materials, the close packing of two-dimensional sheets permits interplanar charge transport in the third dimension, allowing the delocalisation of charge over several molecular layers [107]. The complex models used to describe charge transport in organic materials with differing degrees of disorder is well described by Liu, Noh, and co-workers [108]. The relative permittivity or dielectric constant (ε_r_(ω)), which influences the screening of the charge carriers within the material is drastically different for inorganic and organic semiconductors. High ε_r_(ω) materials, such as silicon (ε_r_(ω) = 12) or GaAs (ε_r_(ω) = 13), effectively screen the Coulombic attraction between excitons to 10s of meV, allowing the charge carriers to easily dissociate at room temperature [109–110]. Organic polymer materials, such poly(*p*-phenylene vinylene, ε_r_(ω) = 2), typically have low ε_r_(ω) values, which prevents the screening of excitons and leads to much stronger binding energies of 0.1–1 eV, which greatly exceeds the available thermal energy at room temperature [111–113]. This is problematic for photovoltaics and photocatalysts but a useful property for OLEDs where the radiative decay of the charge recombination is desired [112,114].

**Figure 8:**
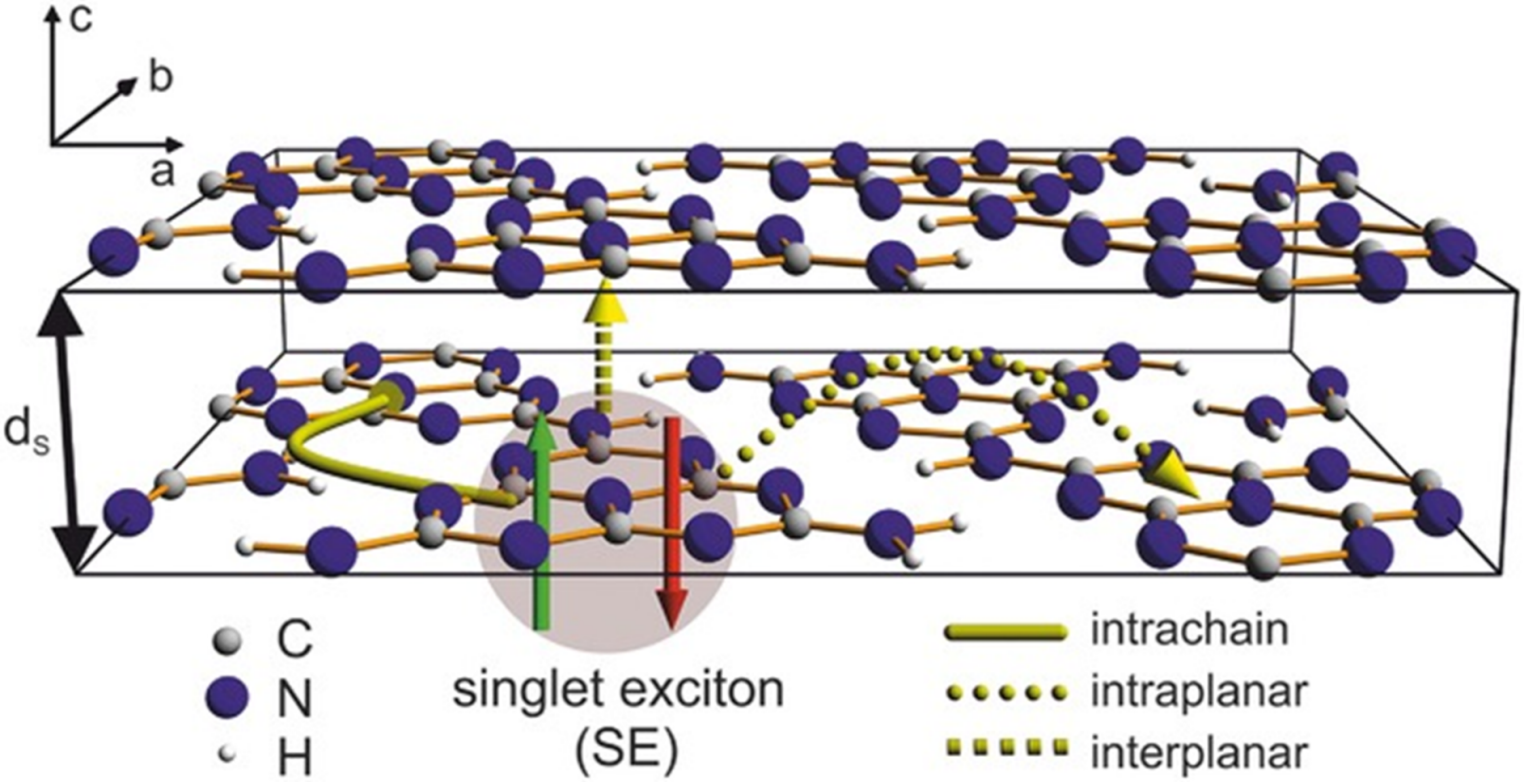
Idealised triclinic unit cell of a g-C_3_N_4_ type polymer, displaying possible hopping transport scenarios of an exciton via intrachain (solid), intraplanar (dotted) or interplanar (dashed). Reprinted with permission from [107], Copyright 2015 Wiley-VCH.

Charge carriers are normally localised to only a few atoms and cause large distortions in the local electronic structure. This small spatial confinement of the photogenerated excitons is normally only a few nm^3^ and accounts for the large binding energy, as opposed to in a Si crystal, which has an exciton diameter in the order of 10s of nm^3^ [115]. Organic molecules will typically have large geometric relaxations to cope with the localised loss of conjugation and polarisation of the surrounding medium, which is enhanced by low ε_r_(ω) values [115–118]. The charge is transported in organic materials by “hopping” between the quantum states. The amorphous nature of most polymers produces a low symmetry and anisotropy for the charge transport. Hence, the charge mobility in organic semiconductors is typically much lower than in crystalline inorganic materials [115]. Conjugated polymers that are rigid will generally have faster intrachain charge transport rather than interlayer hopping transport (Figure 8) [107,115]. The process is highly dependent on the temperature and electronic disorder as each hop requires the reorganisation of the molecules in the chain [115].

**Figure 9:**
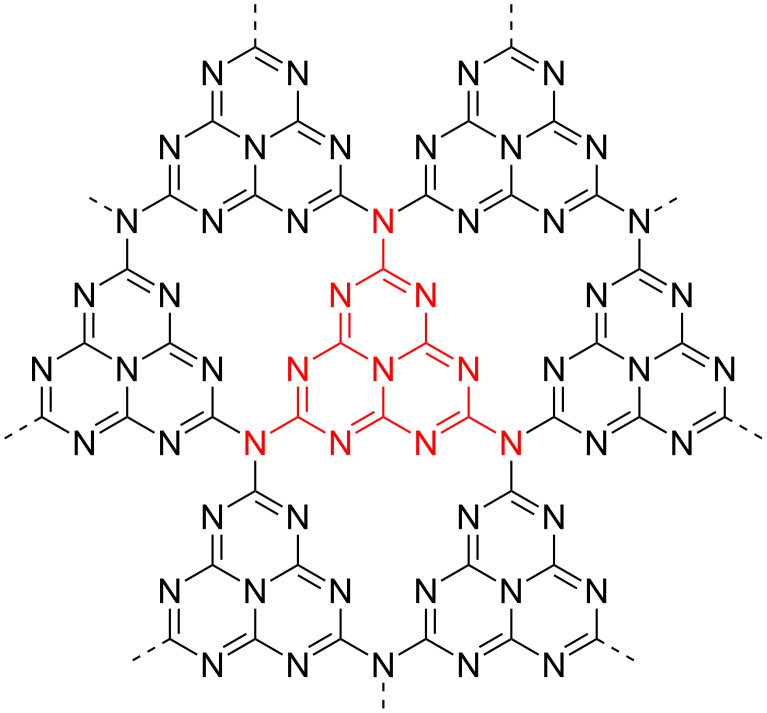
Idealised structure of a perfect g-C_3_N_4_ sheet. The central unit highlighted in red represents one tri-s-triazine (melem) unit [120].

A particularly popular organic semiconductor photocatalyst in the recent literature is graphitic carbon nitride (g-C_3_N_4_) [23]. g-C_3_N_4_ was one of the first synthetic polymers, first reported in 1834 by Liebig, which he named “melon” [119]. The material consists of two-dimensional sheets of hexatopic, hexagonal sp^2^-hybridised carbon and nitrogen atoms, linked by bridging tertiary amines (Figure 9) [120]. The material is crystalline as sheets are held together by strong π-stacking and Van der Waals interactions, rendering the material insoluble in most solvents [120–121]. g-C_3_N_4_ materials are typical organic semiconductors with band gaps of approximately 2–5 eV, usually showing a maximum absorbance at 420 nm, which yields its characteristic yellow colour [120].

**Figure 10:**
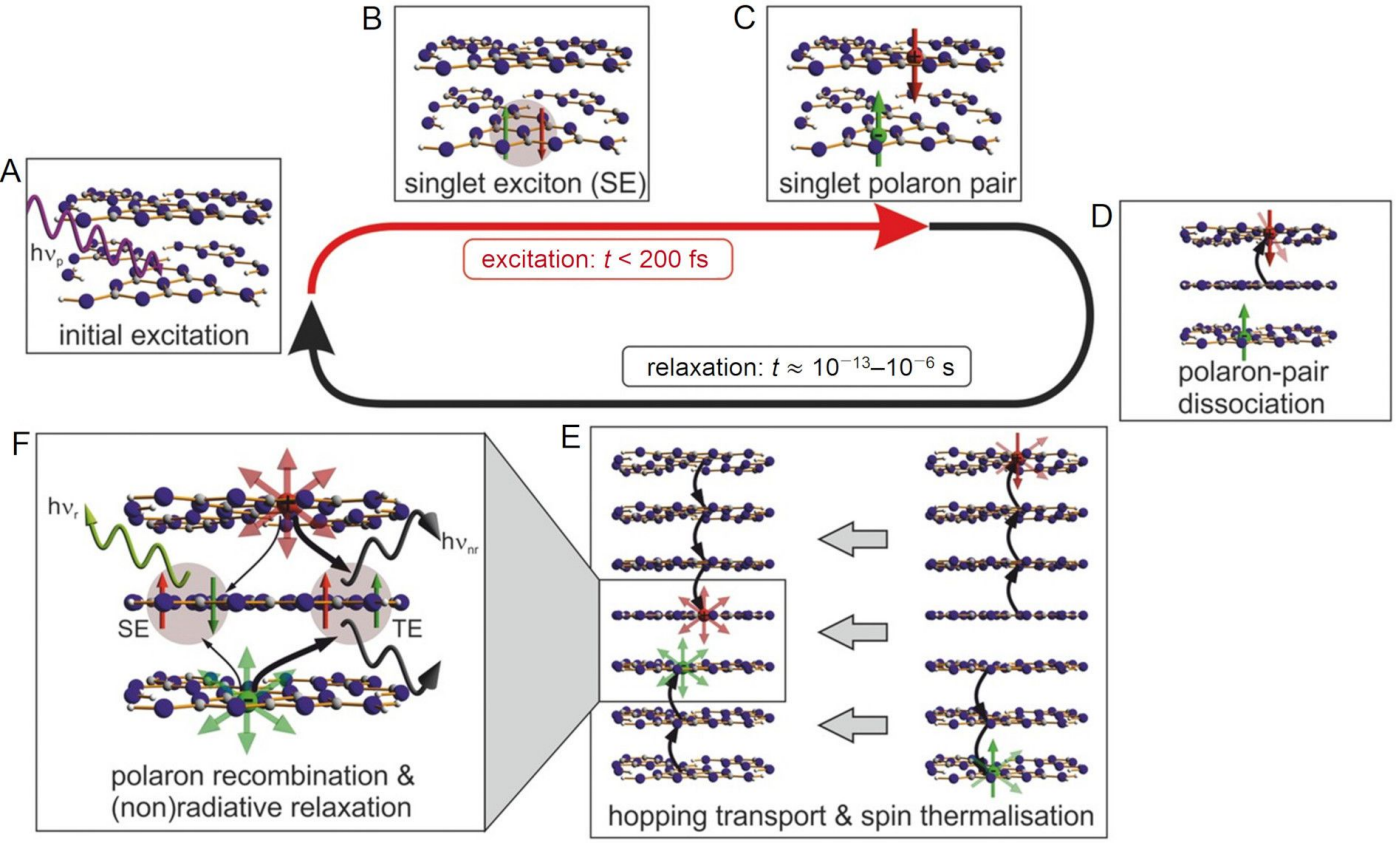
Timeline of the key processes of charge transport following the photoexcitation of g-C_3_N_4_, leading to the radiative recombination of a singlet exciton or the non-radiative recombination of a triplet exciton. A) and B) Photoexcitation and generation of singlet excitons (SE). C) Dissociation of the SE into singlet polaron pairs on adjacent sheets, and D) further dissociation into free polarons. E) Diffusive Brownian motion of the free polarons, essentially confined within channels along the stacking direction. The spin thermalization is depicted, leading to a loss of the spin coherence. F) Recombination of the free polarons. Depending on the spin states of the polarons, singlet (SE) or triplet (TE) excitons are recovered, which relax radiatively (SE) or non-radiatively (TE). Reprinted with permission from [107], Copyright 2015 Wiley-VCH.

Significant interest in g-C_3_N_4_ photocatalysts was generated in 2009 by Wang, Domen, and co-workers, who reported the metal-free photolysis of water was possible with the all-organic semiconductor material, a sacrificial electron donor, and visible light irradiation [120]. Prior to this report, poly(*p*-phenylene) had been utilised for hydrogen production but using high intensity, short-wave UV radiation, and only a modest efficiency was obtained [122]. As a result, research into g-C_3_N_4_ grew exponentially and paved the way for the development of many other organic materials for photocatalysis applications [120,123–127]. Despite its structural similarities to graphite, the charge carrier mobility in g-C_3_N_4_ is significantly different. Transient absorption and transient photoluminescence (TRPL) spectroscopy analysis of a set of g-C_3_N_4_ materials found that the charge transport was predominantly interplanar, perpendicular to the sheets [107]. The work of Merschjann and co-workers critically analysed the non-exponential TRPL spectral decay of g-C_3_N_4_ and found that the initial photon excitation and subsequent exciton dissociation into free polarons (quasiparticle equivalent to e^−^_CB_ and h^+^_VB_ charge carriers) occurs over 200 femtoseconds. The resulting polarons migrate by “hopping” between the sheets until they eventually recombine over a period of approximately 10^−13^–10^−6^ s (Figure 10) [107].

**Scheme 2:**
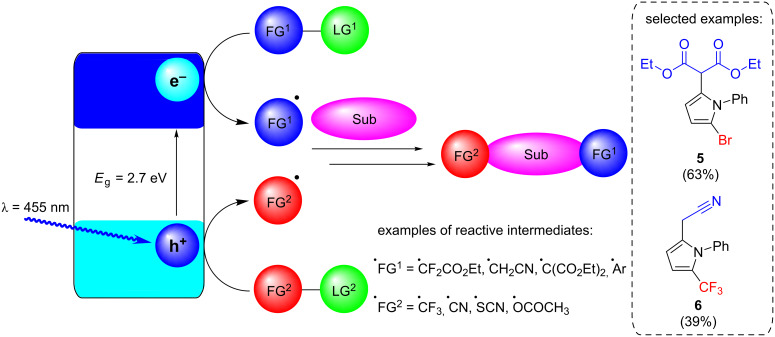
Photocatalytic bifunctionalisation of heteroarenes using mpg-C_3_N_4_, with the selected examples **5** and **6** [128]. Sub = heteroarene substrate (e.g., *N-*phenylpyrrole), FG = functional group, LG = leaving group (e.g., Br, SO_2_Na).

Mesoporous graphitic carbon nitride (mpg-C_3_N_4_) has recently been reported by Antonietti, König, and co-workers to be an effective, stable, and recyclable HPCat for a large scope of arene and heteroarene functionalisation reactions, traditionally performed with transition metal complexes (Scheme 2) [128]. The group were able to bifunctionalise *N-*phenylpyrrole with a variety of functional groups at room temperature to yield alkyl, halo, and trifluoromethyl products, such as the examples **5** and **6** (Scheme 2).

**Figure 11:**
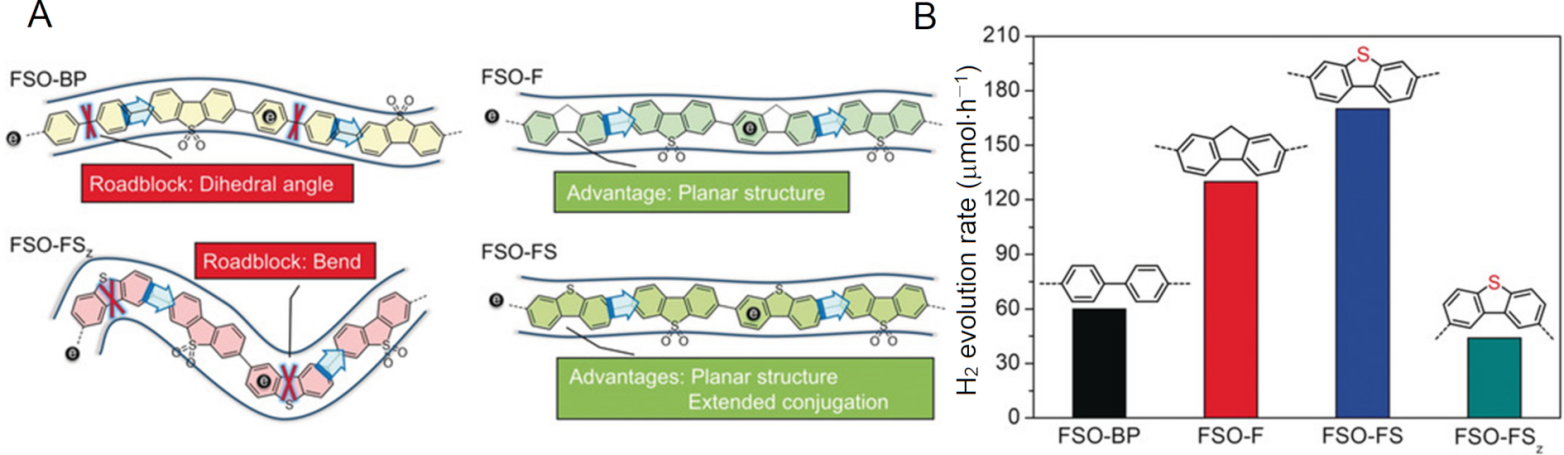
A) Structure of four linear conjugated polymer photocatalysts for hydrogen evolution, displaying the advantages and disadvantages obtained from each monomer and FSO combination with respect to exciton binding energy. B) H_2_ evolution rate of each polymer, reflecting the relative charge separation energy, rate of charge transport and excited state lifetimes being optimal in the FSO-FS polymer. Adapted with permission from [129], Copyright 2019 Wiley-VCH.

Chen, Wang and co-workers recently studied methods to reduce the exciton binding energy in linear conjugated polymers to enhance the charge separation and subsequent photocatalytic hydrogen evolution (Figure 11) [129]. Four conjugated polymers containing dibenzothiophene sulfone (FSO) monomers, linked by either biphenyl (FSO-BP), fluorene (FSO-F), 2,8-dibenzothiophene (FSO-FS_z_), or 3,7-dibenzothiophene (FSO-FS) monomers were synthesised and applied in the photocatalytic hydrogen evolution reaction (HER). The FSO-FS polymer displayed the highest rates of hydrogen evolution (170 μmol⋅h^−1^), significantly higher than its structural isomer FSO-FS_z_. The authors studied the charge transfer dynamics of the different polymers through temperature-dependent photoluminescence, photoelectrochemical measurements, TRPL spectroscopy, and density functional theory (DFT) calculations. They found that the HER efficiency correlated to the excited state lifetime and exciton binding energy. The FSO-BP and FSO-FS_z_ hindered the charge transfer and mobility due to the phenyl–phenyl dihedral angle or sharp bends in the polymer chain, whereas the FSO-F and FSO-FS polymers had ridged planar chains that facilitated the charge transfer [129]. The FSO-FS dibenzothiophene unit had an extended conjugation onto the sulphur atom in the excited state, which improved the charge delocalisation [129]. This demonstrated that novel material design can overcome typical limitations of organic semiconductors to produce more efficient photocatalysts with an enhanced charge transport. The authors did not discuss the formation of triplet excitons through intersystem crossing, which is facilitated by sulphur spin–orbit coupling, as shown by Ji, Zhao, Jacquemin, and co-workers [130], and may have contributed to extending the excited state lifetime and reducing the binding energy of excitons in FSO-FS relative to FSO-F.

#### 2.3 Immobilised molecular photocatalysts

A strategy that combines many of the benefits of homogenous and heterogeneous photocatalysis is immobilising a molecular photocatalyst to a solid support. Most materials have inherent surface functionalities or can be modified to act as supports through covalent or electrostatic interactions, presenting a plethora of strategies to immobilise any photocatalyst with a complimentary functional group. As the immobilised photocatalyst is generally non-conjugated to the support, its photophysical properties are generally unaltered from the homogeneous equivalent, and the complex semiconductor charge dynamics are avoided. Alternatively, semiconductor supports can be used that can be irradiated at different wavelengths and inject electrons to the photocatalyst or vice versa. The same methodology is commonly applied in dye-sensitised solar cells [102]. Additionally, as only the surface of the support is functionalised with photoactive units, they are more likely to be accessible to the reaction media and the excitation photons, preventing the photocatalyst from being wasted in the bulk material. The immobilisation will generally reduce the reaction kinetics of the homogeneous photocatalyst as it is no longer dispersed in solution, but this can be mitigated through flow chemistry as previously discussed. This combines the ease of separation and recyclability of a heterogeneous catalyst, with the detailed characterisation, accessibility and synthetic versatility of a homogeneous photocatalyst.

Some desirable properties of a solid support are hence; (i) a strong, irreversible affinity for the catalyst to the support surface, (ii) stability towards the reaction conditions and reactive photogenerated intermediates, (iii) transparency to the wavelengths of radiation used to excite the catalyst, and preferably elastic scattering of the incident light, (iv) a high surface area and porosity, and (v) a strong affinity for the association of reactants to the surface and rapid dissociation of photochemical products.

**Figure 12:**
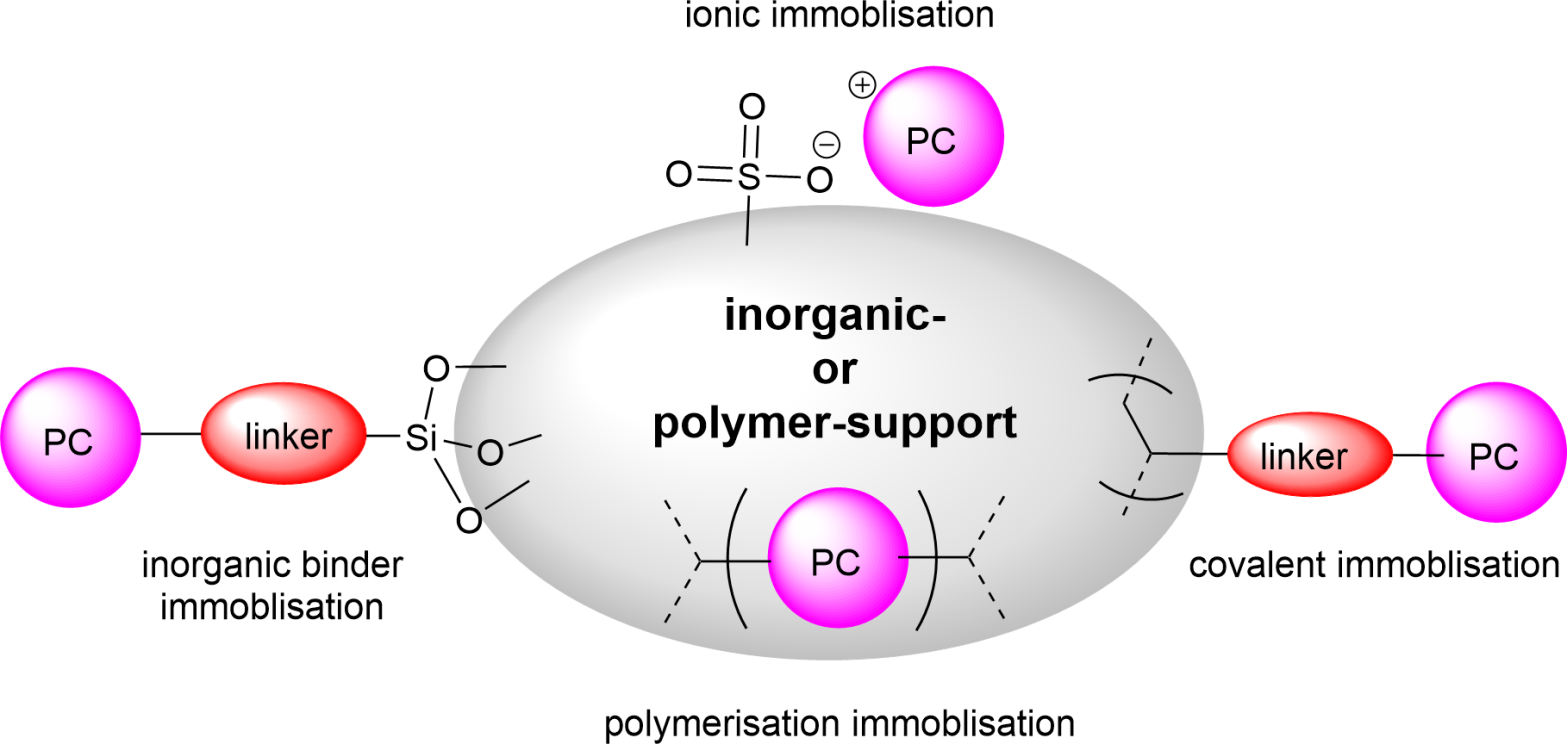
Graphical representation of the common methods used to immobilise molecular photocatalysts (PC) onto solid supports.

A few of these strategies are represented in Figure 12 and discussed in more detail within this section. Specific examples and applications are also given later in the review (see Section 4).

Some example methods of immobilising catalysts include covalently bonding the catalyst to a polymer support either by incorporating a monomer with a photocatalytic moiety or post synthetically coupling a photocatalyst to a polymer via direct synthesis. Examples of this from our own group include the synthesis of a polystyrene gel with a photocatalytic cross-linking monomer, 4,7-bis(4-vinylphenyl)benzo[*c*][1,2,5]thiadiazole (St-BTZ) [47]. Additionally, we showed the direct synthesis of a BODIPY photocatalyst as a postsynthetic modification to an aldehyde-functionalised conjugated polymer and applied the material for singlet oxygen photosensitisation [131]. Both of these were applied as HPCats in a commercial flow reactor for photocatalysis. Whilst flow chemistry is generally used to enhance the efficiency of HPC processes, we recently demonstrated that it can also enhance the efficiency of synthesising and purifying immobilised photosensitisers [63]. A BODIPY photosensitiser was immobilised to an aldehyde-functionalised Merrifield resin (poly(styrene-*co*-divinylbenzene-*co*-4-formylstyrene)) in continuous flow and concurrently in an analogous batch procedure. The coupling efficiency was initially found to be almost double in flow versus batch [63]. The materials were also purified in flow and Soxhlet extractions, respectively, with the same solvent mixtures and time periods. The Soxhlet-extracted batch HPCats were found to have significant quantities of the homogeneous BODIPY still trapped in the resins after the extraction, which led to a dramatic loss in photosensitisation efficiency over multiple recycles as it was progressively washed out. The flow-purified resins displayed no loss of efficiency over the recycles, indicating the flow purification had effectively removed the homogeneous photocatalyst. The flow synthesis also prevented the formation of a chlorinated byproduct identified on the batch resins as an intermediate, and purification could be applied to wash out reactants from the first step of the one-pot synthesis, a potentially useful synthetic advantage of postsynthetic modifications in flow [63].

Poliakoff, George, and co-workers reported a porphyrin photosensitiser ionically immobilised to sulphonate-cross-linked ion exchange polystyrene resins (amberlyst-15) for the synthesis of artemisinin (**49**). This resulted in a bifunctional material, which acts as a HPCat and heterogeneous Brønsted acid catalyst, which are both required for independent steps in the synthesis [132]. They suggested that the porphyrin was protonated by the amberlyst-15 sulphonate groups and immobilised to the solid surface by electrostatic forces, rationalising the observed change in porphyrin colour from a purple powder to green when immobilised. The immobilisation of porphyrins onto polystyrene supports has been reported prior to this through covalent and electrostatic attractions but often suffered from poor coupling yields and low loadings [133–136]. Similarly, ionic immobilisation is common with transition metal complexes as they are often inherently cationic species. Amara and co-workers recently showed that [Ru(bpy)_3_]^2+^ could be immobilised on silica particles with a dramatic increase in the efficiency of the photooxidation of terpenes [137]. Polymer networks containing bipyridyl units have been designed to immobilise transition metal complexes through coordinate bonding for the oxidative coupling of amines [138]. [Ru(bpy)_3_]^2+^ units were synthesised with ethynyl groups *para*-substituted to the nitrogen heteroatom on four of the six pyridine rings. The ethynyl groups were subsequently cross-linked by oxidative homocoupling to form a highly cross-linked polymer [138].

Zhang and co-workers recently demonstrated that the dispersibility of polymer nanoparticle-supported benzothiadiazole photocatalysts could be improved in a range of different solvents by copolymerising classical solubilising monomers with the same St-BTZ photocatalyst monomer previously reported by Vilela and co-workers [47,139]. They showed that the supported photocatalyst material was an effective HPCat for pharmaceutically relevant transformations in the optimal solvent for the reaction, despite the native solubility of the photocatalyst being poor in those solvents [139].

#### 2.4 Solid-supported semiconductors and metal nanoparticles

For the same reasons outlined in the previous section, the immobilisation of thin films of semiconductors and the deposition of metal nanoparticle photocatalysts can improve the irradiation, increase surface area, and reduce the redundancy of the bulk material.

**Figure 13:**
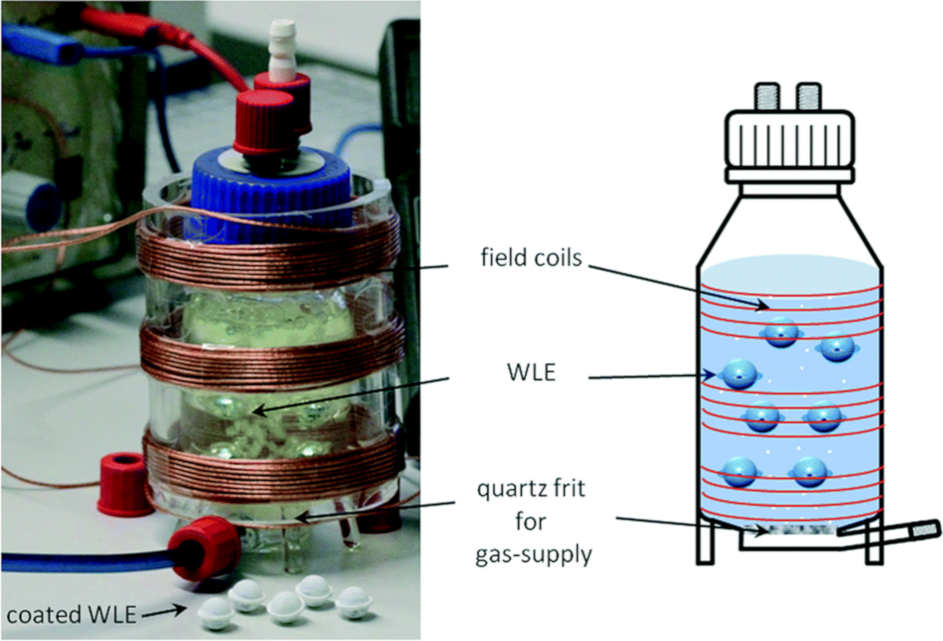
Wireless light emitter-supported TiO_2_ (TiO_2_@WLE) HPCat spheres powered by resonant inductive coupling via field coils wrapped around the reactor. Reprinted with permission from [140], Copyright 2017 The Royal Society of Chemistry.

One particularly interesting approach to a supported TiO_2_ catalyst system was reported by Bloh and co-workers in which a thin layer of TiO_2_ was immobilised onto a spherical SiO_2_ coated polymer support, which encased a wireless LED light source (TiO_2_@WLE, Figure 13) [140]. This allowed the efficient irradiation of the TiO_2_ HPCat from within the reaction mixture and has the benefit of not requiring external LED sources or transparent reactor vessels. The LEDs were powered wirelessly using resonant inductive coupling, and were used for the photocatalytic generation of H_2_O_2_, the degradation of methylene blue dye, and the reduction of nitrobenzene to aniline. Although it was an interesting and novel approach to efficiently irradiating HPCats, the report did not address the potential concerns with the heat dissipation from the LEDs, which could be problematic when scaling up. Additionally, the attrition of the free floating TiO_2_@WLE spheres, leading to potential damage or leaching of the TiO_2_ layer, was not investigated.

The excellent chemical and thermal stability of metal alloys makes them ideal supports for applications in high-pressure and -temperature systems, such as stainless steel (FeCrAl) substrates used in car exhaust catalytic converters [141–142]. TiO_2_ has been deposited on stainless steel previously by thermal atomic layer deposition [143], dip-coating [144], chemical vapour deposition [145], and plasma treatment [146]. Dip-coating has also been commonly applied to coat other inorganic substrates, such as glass spheres, carbon nanotubes, and diatomite with TiO_2_ thin films for photocatalysis applications [147–150]. Scaiano and co-workers deposited platinum nanoparticles on TiO_2_(P25) powder as a stable HPCat for reductive dehalogenations and cyclisations, and reported its enhanced photocatalytic activity relative to unmodified TiO_2_ [151].

Electrochemical deposition techniques, such as electrospray and electrospinning, can also be applied to form photocatalytic composite materials. This has been shown for MnO_2_ deposited on titanium sheets and nanotubes for photocatalysis [152]. Ray and Lalman deposited nanofiber TiO_2_ to a metal support via electrospinning a solution of titanium tetraisopropoxide and polyvinyl acetate (PVAc) to form a TiO_2_-PVAc composite material. TiO_2_-PVAc was subsequently calcinated at 400–500 °C to remove the organic material and form pure TiO_2_ nanofibers, which were applied for the photocatalytic degradation of phenol [153].

Photodeposition is commonly used to deposit metal atoms or nanoparticles on semiconductor surfaces, which can greatly influence the photophysical properties of the semiconductor substrate through their localised surface plasmon resonances (see Section 2.5). Alternatively, they can receive charge carriers from the HPCat support to drive a cocatalyst cycle and simultaneously facilitate the charge separation across a heterojunction, hindering recombination [154–155]. Suárez and co-workers used the photodeposition of platinum onto tungsten oxide (WO_3_) powder, before immobilising within a zeolite framework to produce visible light-active HPCats for the degradation of pollutants in air [156]. Scaiano and co-workers have also utilised photodeposition to fuse samarium oxide nanoparticles to TiO_2_ and ceria (CeO_2_) as a bifunctional heterogeneous photoredox Lewis acid catalyst for reductive cyclisation reactions, previously reported with ruthenium transition metal complex photocatalysts [157].

Both electrochemical and photochemical deposition techniques have recently been shown to have interesting enhancements when coupled with ultrasonic irradiation [158]. Ultrasonic irradiation, from 20 kHz to 1 GHz, does not directly interact with molecular energy levels. However, the extreme conditions generated from an ultrasound wave expanding within a liquid medium are capable of breaking chemical bonds, including the sonolysis of water to hydrogen and hydroxyl radicals [159]. These conditions arise from a process known as acoustic cavitation in which the expanding phase of the acoustic wave creates microscopic voids within the solution, which collapse by implosion, producing conditions equivalent to approximately 5000 °C and 1800 atm at the implosion centre [160].

The resulting sonoelectrodeposition and sonophotodeposition methodologies have been shown to enhance mass transfer during synthesis and provide more control over the morphology and size distribution of the deposited nanoparticles [158]. Colmenares and co-workers have reported these methodologies for the synthesis of TiO_2_ HPCats doped with various transition metals, such as Fe, Pd, Pt, and Au [159–161]. For more information, we recommend a recent review published by Magdziarz and Colmenares [158].

#### 2.5 Other heterogeneous photocatalyst materials

The section above broadly categorised HPCats as either inorganic semiconductors, organic semiconductors, immobilised molecular photocatalysts, or solid-supported semiconductor HPCats. There are of course a vast number of different examples for each of these categories, with subtle differences between them; inorganic semiconductors other than TiO_2_, such as ceria (CeO_2_) [157], CdS [162], polyoxometalates [163–164], quantum dots [165–168], and many other examples [18,169–170]. Organic semiconductors could include conjugated porous polymers (CPPs) [3,10,171], covalent organic frameworks (COFs) [172–175], and hybrid materials, such as metal–organic frameworks (MOFs) [1,176–177]. MOFs, for example, have three distinct origins of photocatalysis; (i) semiconducting metal cluster nodes, (ii) photocatalytic organic ligands (struts) that connect the nodes, and (iii) as a solid support for other HPCat materials immobilised in the void spaces of the porous material [1,176–180]. Each material has its own subtle differences but generally adheres to the fundamental principles discussed in Sections 2.1 and 2.2, and can be explored in detail in independent reviews [1,6,176,181–182].

Some examples are more unique, such as metal nanoparticle (NPs) photocatalysis. Despite having a conductor band structure,and therefore no band gap, the nanoscale of these materials produces visible light absorptions. This is due to the oscillating electron density within metallic NPs, which can couple with the electric field of photons with wavelengths similar to the dimensions of the NPs [181]. This is called the localised surface plasmon resonance (LSPR), and is responsible for the brilliant colours observed in colloidal solutions of noble metal NPs [6,181–187]. The photon absorption polarises the electron density of the nanoparticle and produces energetically excited electrons above, and holes below the Fermi level that can undergo photoredox processes with substrates [185,188–190].

**Figure 14:**
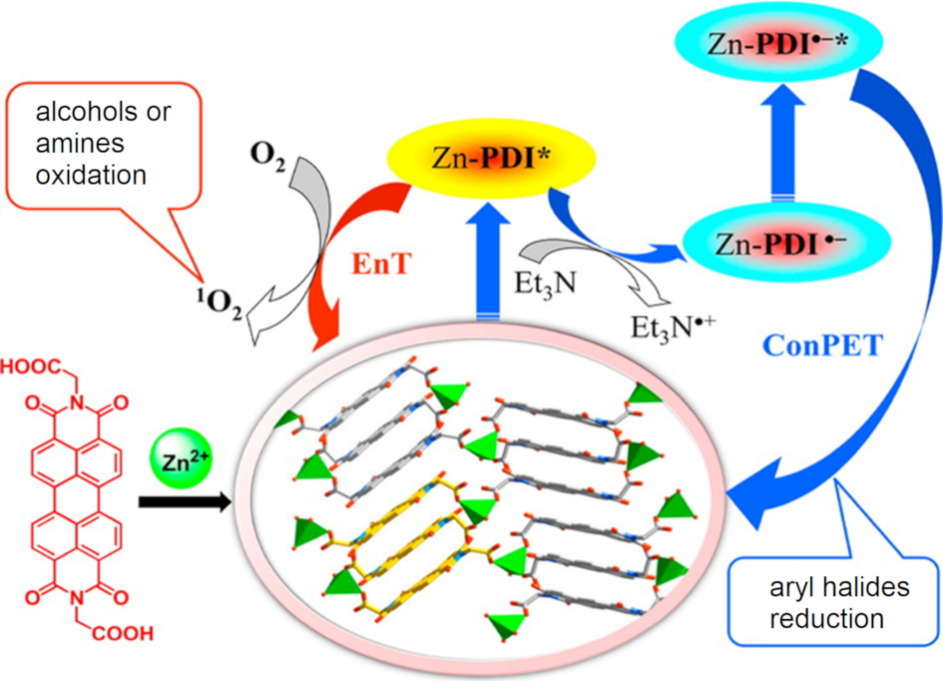
Graphical representation of zinc–perylene diimide (Zn-PDI) supramolecular assembly photocatalysis via consecutive photoinduced electron transfer (ConPET). EnT = energy transfer, ^1^O_2_ = singlet oxygen (see Section 4.2). Reprinted with permission from [193], Copyright 2016 American Chemical Society.

Supramolecular photocatalysts are an interesting class of materials which utilise non-bonding interactions to control photochemical processes, a biomimetic strategy that imitates enzyme catalysis [191]. Supramolecular self-assemblies and coordination polymers based on perylene diimide (PDI) aggregates have become a popular choice of heterogeneous photocatalyst. As PDI is a large, planar, polyaromatic hydrocarbon molecule, it spontaneously forms ordered 1D supramolecular assemblies through efficient π–π stacking and side chain interactions [192]. Despite the PDI units having only non-bonding interactions, the narrow π–π stacking provides a sufficient π orbital overlap to produce semiconductor-type electronic band structures and an efficient interplanar charge transport similar to g-C_3_N_4_ [192]. Duan and co-workers reported the synthesis of a zinc PDI assembly as a heterogeneous photocatalyst for the reduction of aryl halides [193]. The material could undergo consecutive photoinduced electron transfers (ConPET) in which the material enters an excited state and is reduced by a sacrificial electron donor (NEt_3_). The resulting Zn-PDI radical anion then undergoes a second photon absorption to achieve a superreducing electronic excited state capable of reducing stable aryl halides (Figure 14) [193]. More recently, PDI self-assemblies have been reported as efficient HPCats for the hydrogen evolution reaction [194] and degradation of phenol [192].

Another important material design applied in heterogeneous photocatalysis that we have so far not mentioned are Z-schemes and heterojunction photocatalysts [7,195–196]. Z-scheme systems comprise two or more independent semiconductors with similar *E*_g_ values but differing valence band and conduction band potentials. By fusing the two materials to form a heterojunction and concurrently photoexciting both semiconductors, the least reducing e^−^_CB_ and oxidising h^+^_VB_ of the two semiconductors will recombine at the heterojunction. This leaves the more reducing e^−^_CB_ and oxidising h^+^_VB_ of each semiconductor with no opposing charge carrier, preventing recombination and expanding the redox potentials of the fused material [7]. The history and recent developments of these materials was reviewed by Anusuyadevi, Marre, Yu, and co-workers [7,196].

As briefly mentioned in Section 1.3, upconversion photocatalysis is a developing field of research that permits using long-wavelength irradiation to deeply penetrate through a reaction medium and material surface and activate the bulk of the material. Congreve, Rovis, Campos, and co-workers utilised triplet fusion upconversion to drive a series of homogeneous photoredox transformations with >700 nm photons [50]. They reported a photocatalytic atom transfer radical polymerisation for the synthesis of cross-linked PMMA using the upconversion photocatalyst system and either a blue laser or a NIR (730 nm) laser. The blue laser would excite the photocatalyst component directly, whilst the NIR laser activated the sensitiser component, which sequentially transfers the energy to the photocatalyst to achieve the same excited state indirectly. The polymerisations were performed with a variety of light-blocking media placed between the laser source and the reaction medium. This included bacon strips to imitate light transmission through flesh, as this is relevant for photocatalyst applications in photodynamic therapy [197–199]. They reported that the NIR photons penetrated 293 times deeper into the polymerisation medium and enabled the reaction to be applicable on a multigram scale through silicon moulds, which could not be achieved with the blue laser [50].

**Scheme 3:**
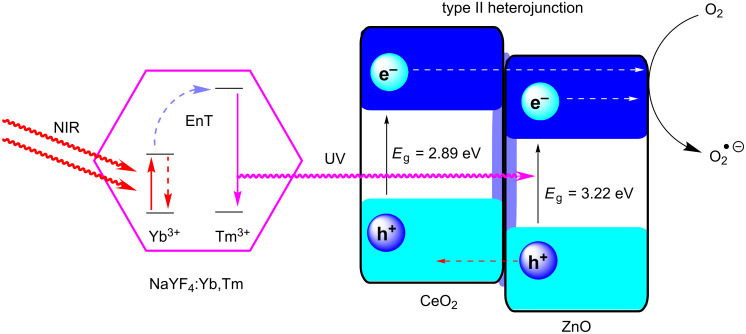
Upconversion of NIR photons to the UV frequency by NaYF_4_:Yb,Tm nanocrystals sequentially coated with CeO_2_ and ZnO HPCat layers. Two or more NIR photons are sequentially absorbed by Yb^3+^ ions (sensitisers), and the energy is transferred to Tm^3+^ ions (activators), which emit a single higher-energy photon. The upconverted photons drive the charge separation in the HPCat layers for the photocatalysis. NIR = near infrared irradiation, UV = ultraviolet photons, EnT = energy transfer. The CeO_2_/ZnO type II heterojunction enhances the charge carrier separation and supresses the recombination [200].

Heterogeneous upconversion systems are generally crystalline nanomaterials comprising a host matrix of typically NaYF_4_, doped with a mixture of lanthanide ions, which act as light-absorbing sensitisers (e.g., Yb^3+^) and energy-combining activators (e.g., Er^3+^, Tm^3+^) [51,200–201]. Li and co-workers reported the synthesis of NaYF_4_:Yb^3+^(20%), Tm^3+^(0.5%) upconversion nanocrystals coated with a thin layer of CeO_2_ and sequentially with a layer of ZnO [200]. This formed a core–shell nanocomposite of the upconversion nanocrystals, coated in a type II heterojunction photocatalyst system (Scheme 3) [7,200]. The material was applied in the photocatalytic degradation of an organic dye, methyl orange, and was shown to have direct photocatalytic activity when irradiated with visible or NIR radiation. Additionally, the activity of the coated upconversion nanocrystals under simulated solar irradiation was greatly enhanced relative to pure CeO_2_, as the composite material could utilise shorter wavelength irradiation directly, as well as longer wavelengths via the upconversion system [200].

Hao, Jin, Du, and co-workers reported a similar strategy of coating octahedral YF_3_:Yb,Tm microparticles with a thin layer of epitaxially grown BiOCl HPCat [201]. The epitaxial interface was found to be important for the efficient non-radiative energy transfer and upconversion emission reabsorption. The group reported similar observations of a direct NIR irradiation-driven photocatalytic ability for the degradation of methyl orange and for water photolysis. Consistent with the report of Li and co-workers, the BiOCl-coated YF_3_:Yb,Tm also showed an improved activity over pure BiOCl under simultaneous visible light (>420 nm) and NIR irradiation, as the composite material could utilise more wavelengths [201]. A few other examples of lanthanide-doped upconversion materials for photocatalysis exist in the literature and were reviewed by Ma and co-workers, who also provided more details on the complex mechanisms upconversion by lanthanide ions [51].

Now that we have established the fundamental processes and recent developments of heterogeneous photocatalysts, we will discuss the development of flow reactor systems designed to apply HPCats before reviewing their application in organic synthesis as well as water and air purification.

### 3 Flow reactors for heterogeneous photocatalysts

**Figure 15:**
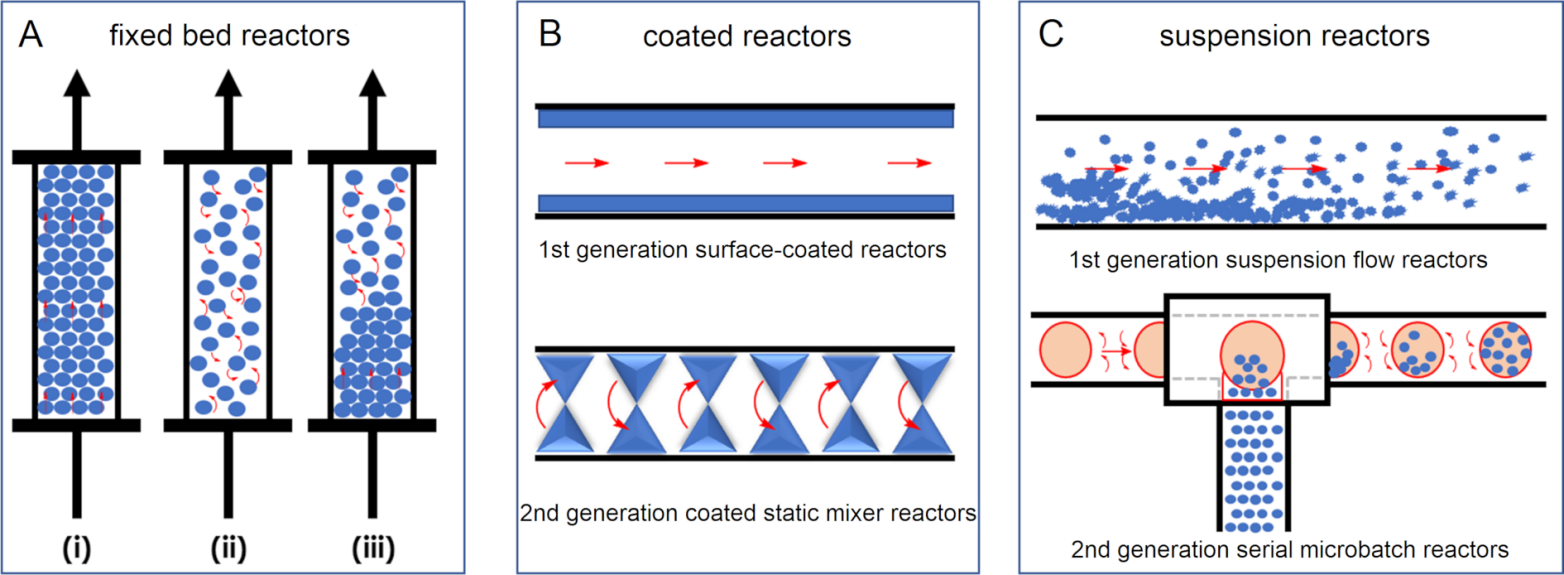
Types of reactors employed in heterogeneous photocatalysis in flow. A) Fixed bed reactors and the subcategories of (i) packed bed, (ii) fluidised bed, and (iii) hybrid/mixed bed. B) Coated reactors in which the HPCat is immobilised onto a surface. C) Suspension reactors that have the HPCat freely flowing through reactor channels.

The consideration of different reactors that can be employed for irradiating heterogeneous photocatalysts is important for maximising the efficiency of the intended system. Homogeneous photocatalysis is normally a simple choice between a three-dimensional transparent coil or a two-dimensional microfluidic device, with the common objective of generating a short path length for the efficient and uniform irradiation of a solution flowing through a narrow channel. HPCats have three main reactor categories for implementation in flow; (i) immobilised within a fixed bed, (ii) immobilised through coating to a reactor’s surface, or (iii) a free-flowing suspension (Figure 15). Within these categories are further subdivisions or evolutions that each carry unique advantages and disadvantages, which we will now discuss in more detail.

Fixed bed reactors are typically transparent columns made from inert materials, such as borosilicate glass, sealed at each end by porous frits that allow liquids to flow through the reactor, but not solids. External light sources irradiate from the sides, as liquid reagents flow through the bed and into contact with the immobilised HPCat. Annular fixed beds may also have a light source placed within the central cavity for enhanced irradiance. As the HPCat is confined to the reactor, no filtration of the outflow is required to recover the HPCat, and the reactor can be reused continuously.

As depicted in Figure 15A, depending on the free volume, the direction of the flow, and the relative densities of the solvent and HPCat, the fixed bed can be utilised in three different modes of operation: (i) a packed bed in which the HPCat fills the volume of the reactor, forcing the solution to flow through the material’s pores or voids between the compact material surfaces, (ii*)* a fluidised bed in which the bed has the free volume for the HPCat to disperse and move freely in the flowing solution whilst remaining confined to the reactor, and (iii) a mixed bed, a hybrid between the two aforementioned regimes in which the reactor has a free volume, but portions of the HPCat are compacted and the rest is mobile. The three regimes have different advantages and disadvantages, which we have outlined in Table 1. Mixed/hybrid bed reactors have a combination of advantages and disadvantages of both the packed and the fluidised bed reactors and therefore have been omitted from the table. The more intricate details of the fluid dynamics and reactor engineering are beyond the scope of this review but can be explored in engineering text books and review articles [43,202–206].

**Table 1:** Advantages and disadvantages of fixed bed reactor modes of operation.

packed bed		fluidised bed	
advantages	disadvantages	advantages	disadvantages

* The solution is forced into contact with the immobilised catalyst.	* The flow of immiscible liquid/liquid or liquid/gas mixtures through the bed is difficult.	* Improved mixing removes hotspots, which can enhance the selectivity in thermal processes [202–203].	* The free volume allows some reagents to pass through the reactor without necessarily contacting the HPC.
* The catalyst is stationary, reducing material damage through attrition.	* Poorer mixing can reduce the selectivity and produce hotspots within the reactor.		
* The confinement of the HPCat to the reactor creates a high relative concentration of the catalyst compared to the flowing reagents.			

Fixed beds are therefore a versatile choice of reactor for heterogeneous photocatalysis in flow. However, fixed bed reactors suffer from a relatively low surface-to-volume ratio compared with the dimensions of a microchannel, shielding the HPCat packed within the centre of the reactor from irradiation [207]. This is often overcome by copacking an inert, transparent material, such as glass beads, with the HPCat to disperse the photoactive material [208–209]. Fixed beds are generally quite expensive and limited to column or annular designs, which makes controlling the mixing and the sequential addition of the reagents generally not possible.

Coated reactors immobilise the HPCat as a thin film on the surface of channels within a microfluidic device [210–211]. Coated reactors overcome the disadvantages of fixed beds by permitting much greater freedom of design with respect to the channel dimensions and the flow paths. This enabled custom flow channel designs that can precisely control the addition of reagents, residence time, and mixing, optimised for the specific reaction [32,58,212]. As the active HPCat is only present as a thin layer on the channel surface, it can be efficiently irradiated.

In first generation coated reactors, the HPCat is confined to the surface of linear flow channels. This presents mass transport limitations as reagents must diffuse to the channel surface to react, making the channel surface-to-volume ratio a limiting factor for the productivity [213]. Second-generation coated reactors combat this issue by employing static mixers as the HPCat substrate to generate a turbulent flow and to enhance the mass transport within the channels [214–217]. There are limitations as the HPCat and reactor device must have compatible functional groups to be deposited or immobilised and must be compatible with the intended reaction system’s solvents and reagents. The freedom of design advantage also carries the disadvantage that these reactors have a limited commercial availability and often require manufacture by the intended user.

The final type of the reactor provides solutions to the limitations of immobilising HPCats by not immobilising the HPCat. Suspension or slurry reactors operate by dispersing the HPCat as small particles within the reaction solution and pumping them together through a flow reactor system. Most homogeneous photocatalysis reactor setups will be suitable for an HPC suspension flow so that there is no need to invest in a reactor exclusively for a HPCat. In order to pump suspensions, either a syringe pump or a peristaltic pump will be required as HPLC piston pumps are not compatible with solids [43]. Care must be taken to prevent the suspended HPCat from sedimenting in the syringe and creating a non-uniform suspension [218]. The HPCat employed must have small dimensions to remain in suspension throughout the flow system, typically a powder with particles <100 μm for channels with 1 mm internal diameter. This may require grinding the HPCat with a mortar and pestle or a ball mill, which may be undesirable for materials with intricate and sensitive nanostructures.

Whilst the HPCat is well dispersed throughout the flowing medium, the local environment around the flowing particles can be relatively static, hence the mass transport of reagents to the suspended catalyst remains suboptimal [207]. Second generation suspension reactors function by dosing HPCats suspended in a viscous ionic liquid into reaction media droplets, which are separated by segments of a carrier gas. The segmented slug flow generates Taylor flow cycles, which efficiently mix the individual suspensions to prevent the sedimentation and enhance the mass transport within the local environment [207]. An issue remains for industrial applications as both generations require filtration for the HPCat recovery and purification, although this is less cumbersome than recovering a homogeneous photocatalyst.

In summary, we have generalised the advantages and disadvantages of each reactor system in Table 2 to guide the reader in choosing the best option for their intended HPCat and reaction system. In the following sections of the review, we will denote the type of reactor used in the reaction schemes by the graphics displayed in the first column of Table 2, for ease of reference.

**Table 2:** Advantages and disadvantages of different HPCat flow reactor systems.

system	advantages	disadvantages

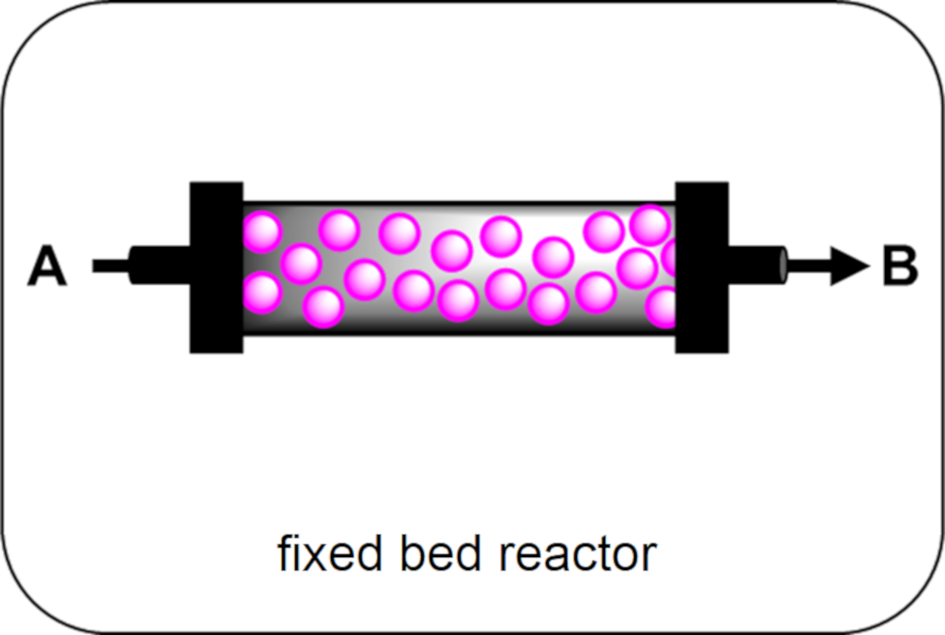	• Multiple modes of operation	• Borosilicate columns are expensive and fragile
	• Compatible with any HPCat & solvents	
	• Deactivated catalyst is easily removed and replaced	• Reactor dimensions are limited
		• Low surface-to-volume ratio prevents efficient irradiation
	• Immobilised catalyst is easily recycled & purified	
		• Pressure drop across the bed can be problematic
	• Commercially available	

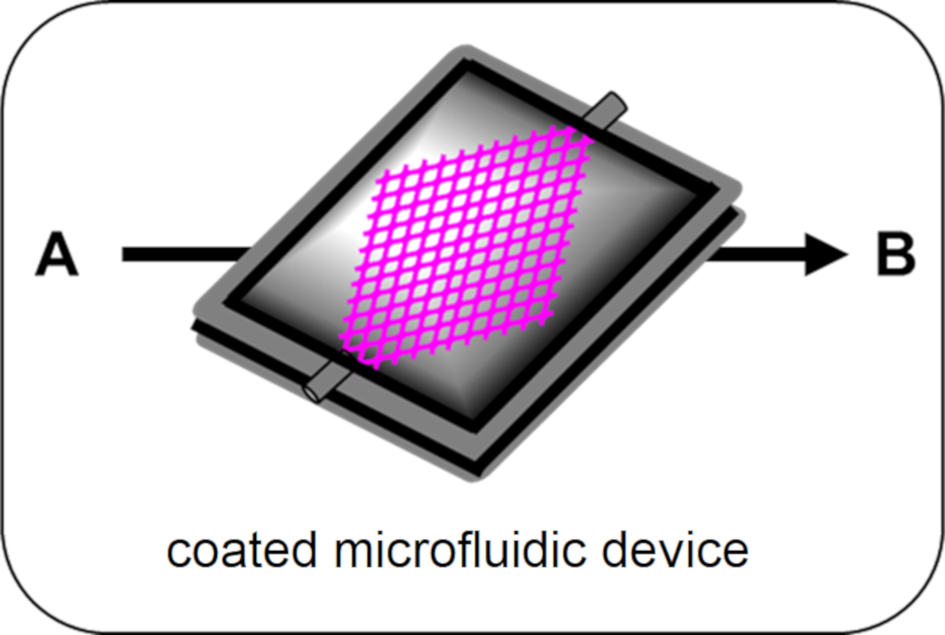	• Freedom of reactor design	• Lack of commercial availability
	• Thin film HPCat efficiently irradiated	• Limited by immobilisation compatibility of reactor material and HPCat
	• High HPCat surface area	
	• Static mixers can be coated to enhance mass-transport	• Reactor material and solvent system compatibility needs consideration
		• Commercial static mixers are expensive
		• Difficult or impossible to regenerate or replace deactivated catalyst

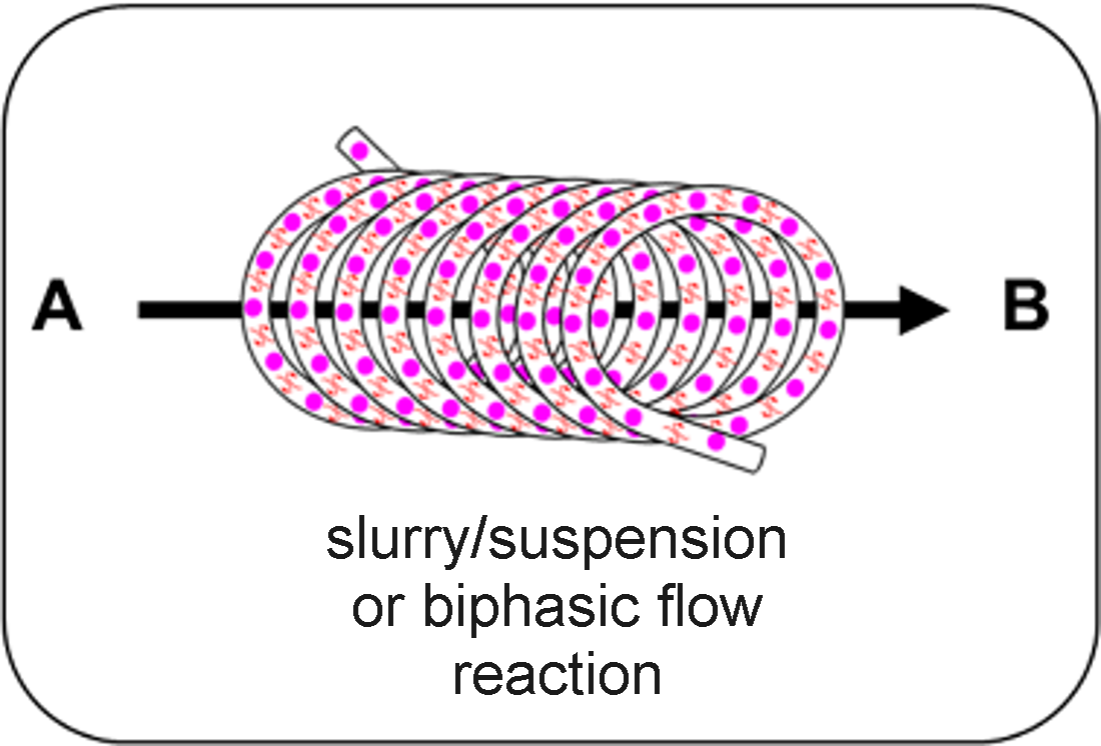	• Easily assembled from cheap materials	• Prone to blockages
	• Efficient mixing and irradiation	• Requires very small HPCat particles
		• Some HPCats and solvents may be incompatible
		• Filtration will be required to reclaim and recycle HPCat

### 4 Applications of heterogeneous photocatalysts in continuous flow reactors

With the fundamental photocatalysis processes and recent design developments of HPCats and their compatible reactor types now in perspective, we will proceed to review the recent reports of heterogeneous photocatalysis in flow. This section is divided into four parts: HPC in flow for organic synthesis through photoredox (Section 4.1) and energy transfer photocatalysis (Section 4.2) and two final sections on water (Section 4.3) and air purification (Section 4.4).

#### 4.1 Heterogeneous photoredox catalysis in flow

Photoredox catalysis was first reported in the 1970s by Kellogg and co-workers [219–220], followed shortly by other reports from early pioneers [219–229], but the field failed to gain momentum due to a lack of cheap and powerful LED technology. Today’s LED technology is becoming increasingly powerful and less expensive, as predicted by Haitz’s law (the LED equivalent of Moore’s law) [230–231]. As discussed previously, this changed in 2008 after three seminal papers from the groups of MacMillan, Yoon, and Stephenson, who used ruthenium complexes and visible light to catalyse single-electron transfer reactions [24–26]. These reports demonstrated that photochemical synthesis could be achieved with visible light, initiating the realisation of Ciamician’s vision of harnessing the power of solar-irradiation to power artificial photosynthesis, stated in “The photochemistry of the future” as [29]:

“[…] inside of these will take place the photochemical processes that hitherto have been the guarded secret of the plants […]”.

**Figure 16:**
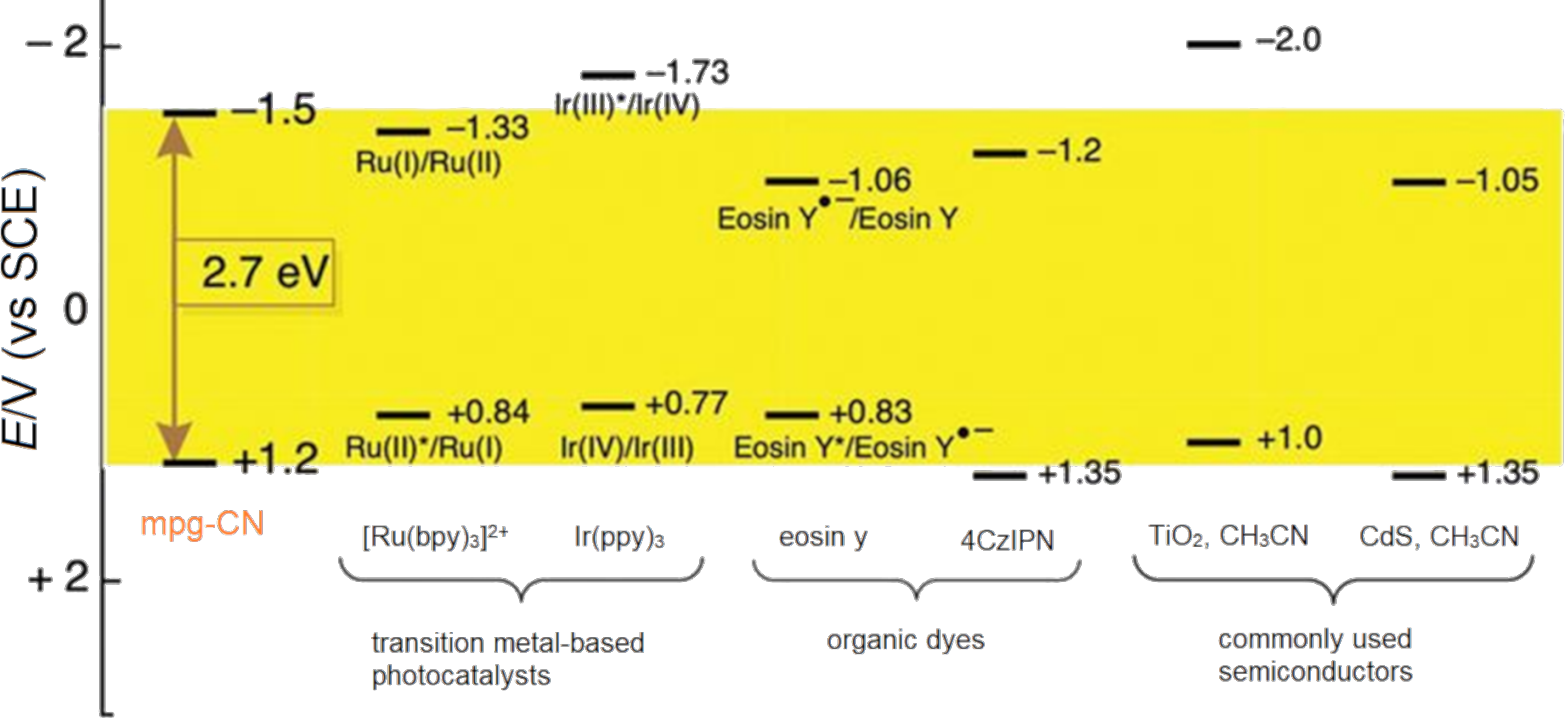
Electrochemical potential of common semiconductor, transition metal, and organic dye-based photocatalysts [128]. Reprinted with permission from AAAS.

Following a photoinduced charge separation event in a semiconductor HPCat or immobilised molecular photocatalyst, the materials’ reduction and oxidation potentials are simultaneously increased by the formation of an excited electron and electron vacancy. The electrochemical potentials of the reaction substrates should be compatible with the HPCats’ excited state potentials, as this largely dictates whether a photoredox process will be successful. This has made electrochemical analysis, such as cyclic voltammetry, a core tool of modern photocatalyst characterisation [232–233]. The electrochemical potentials of some common semiconductor and molecular photocatalysts are displayed in Figure 16 [128].

**Scheme 4:**
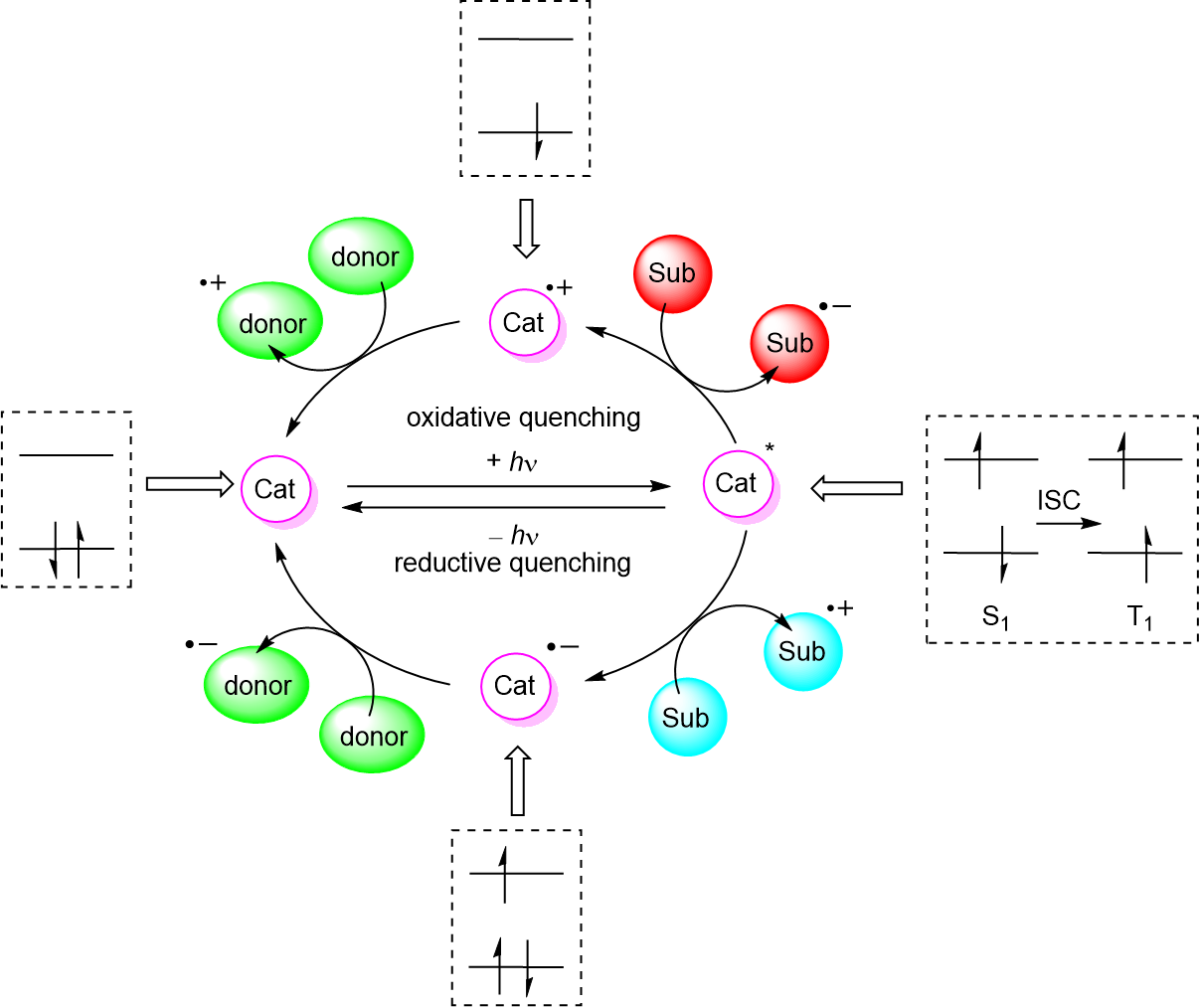
Possible mechanisms of an immobilised molecular photoredox catalyst by oxidative or reductive quenching. ISC = intersystem crossing, Sub = substrate, Cat = photocatalyst, Cat* = electronic excited state photocatalyst, +/- *h*ν = absorption/emission of photon, S_1_ = first singlet electronic excited state, T_1_ = first triplet electronic excited state.

The photoredox cycle then proceeds as illustrated in Scheme 4 for an immobilised molecular photocatalyst, and as previously illustrated in Figure 4 for a semiconductor HPCat. The overall photoredox process can be defined as net oxidative or net reductive, depending on the quenching cycle. The intermediate reduced or oxidised HPCat is returned to its initial state by a sacrificial electron donor or acceptor, respectively. If the initial substrate reacts and forms an intermediate, which subsequently reduces or oxidises the intermediate HPCat to complete the photocatalytic cycle, the process is termed net-neutral photoredox catalysis.

**Scheme 5:**
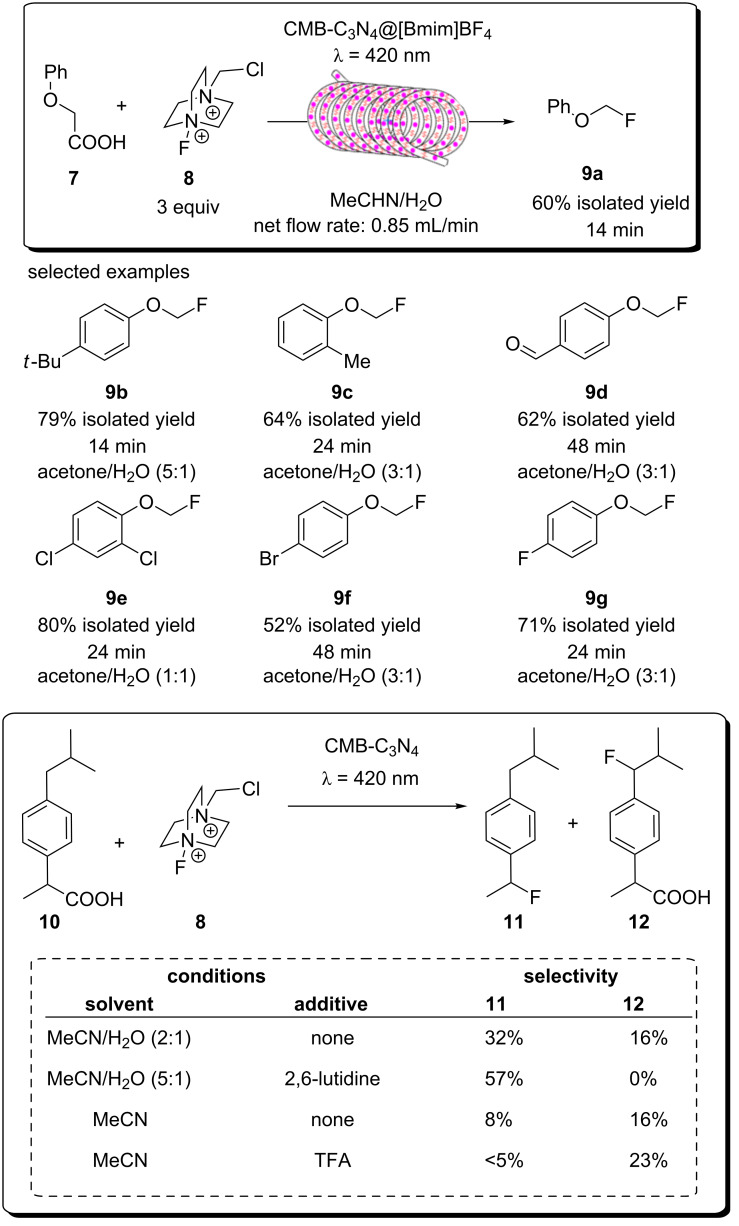
Scheme of the CMB-C_3_N_4_ photocatalytic decarboxylative fluorination of aryloxyacetic acids, with the selected examples **9a**–**g** and the selectivity control in the fluorination of ibuprofen (**10**) being shown [207].

A recent report from Seeberger, Gilmore, and co-workers looked to overcome the inherent disadvantage of light penetration when performing heterogeneous photocatalysis in a fixed bed reactor, as discussed in the previous section [207]. To overcome this, the group optimised a multiphasic system of serial microbatch reactors (SMBRs) in which a modified g-C_3_N_4_ previously reported by Antonietti and co-workers [126] was suspended in an ionic liquid and periodically dosed into a segmented flow of a aryloxyacetic acid (**7**) and SelectFluor (**8**) solution separated by nitrogen pockets (Scheme 5). The liquid/gas slug flow regime generates Taylor flow cycles that efficiently mix the individual liquid slugs and keep the HPCat suspended.

The CMB-C_3_N_4_ is formed of cyanuric acid (C), melamine (M), and 5 mol % barbituric acid (B), which is calcined to yield the modified g-C_3_N_4_ with heterojunctions formed in situ. CMB-C_3_N_4_ has been shown to be superior to g-C_3_N_4_ and mesoporous g-C_3_N_4_ (mpg-C_3_N_4_) in the photocatalytic hydrogen evolution reaction due to a better charge separation across the heterojunction of the material [126]. The suspensions were optimised by manipulating the flow rates of the three component phases until uniform suspensions were produced and maintained by the internal vortices created by the Taylor flow cycles. They reported a broad scope of aryloxyacetic acids with various substitutions on the benzene ring, **9a**–**g** (Scheme 5), with >60% isolated yield under optimised conditions. Interestingly, this transformation has been performed with strongly oxidising homogeneous iridium photocatalysts and a sacrificial base, but no additives were necessary with the modified CMB-C_3_N_4_ HPCat [234]. It is important to note that while the system provided efficient irradiation, the reaction required a three-step extraction process to recover the catalyst, product, and ionic liquid. Fortunately, the HPCat, ionic liquid, and product could all be recovered and purified through sequential filtrations and extractions, without the need for a laborious column chromatography purification. The substrate scope included ibuprofen (**10**), which could be selectively fluorinated to give **11** or **12** by the addition of lutidine or trifluoroacetic acid additives, respectively [207]. The reaction has been well studied with transition metal photoredox photocatalysts and TiO_2_ in batch, and was proposed to occur via the same mechanism [126,207,235–236].

**Scheme 6:**
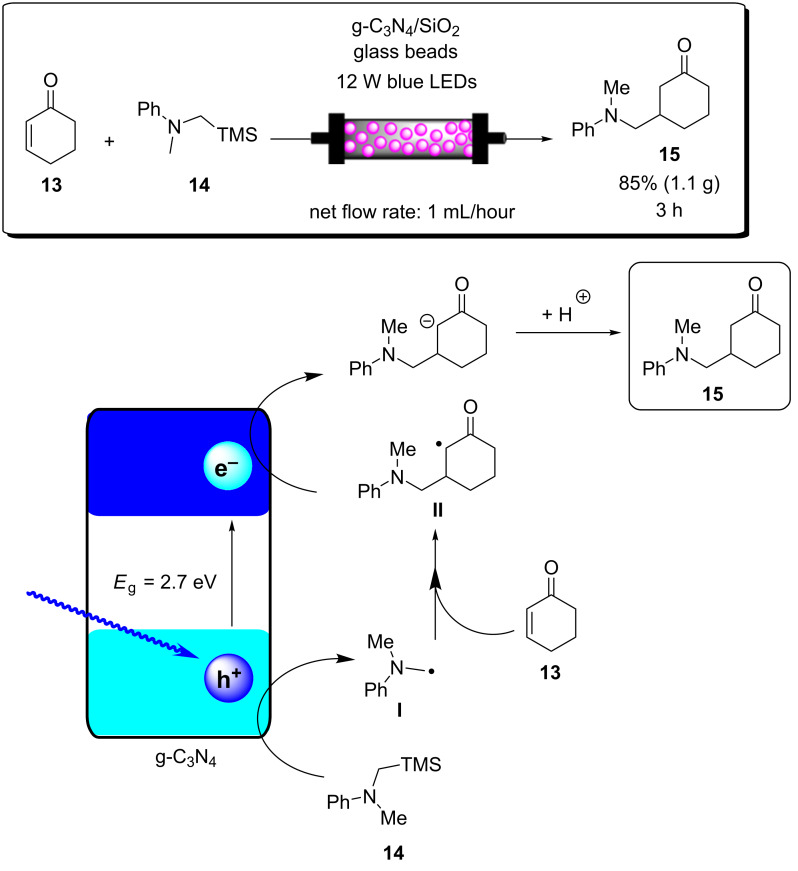
Scheme of the g-C_3_N_4_ photocatalytic desilylative coupling reaction in flow and proposed mechanism [208].

A recent report from Rueping and co-workers utilised g-C_3_N_4_ as a heterogeneous photocatalyst to develop desilylative and decarboxylative couplings to generate secondary and tertiary amine derivatives (Scheme 6) [208]. Following the photoinduced charge separation and migration to the g-C_3_N_4_ surface, α-amino acids and α-silylamines are oxidised by the h^+^_VB_, leading to the subsequent decarboxylation or desylilation, respectively, to yield the common α-amino alkyl radical **I** (Scheme 6), which undergoes addition, allylation, and heteroarylation reactions. The resulting radical intermediate **II** is reduced by the e^−^_CB_ and subsequently protonated to yield the product. The reactions proceeded with good yields of generally >70% across a broad range of substrates in a batch photochemical reactor. The g-C_3_N_4_ HPCat was recycled 8 times in the desilylative coupling of an α-silylamine and acrylonitrile derivative, maintaining >90% conversion after a total of 16 hours of irradiation. The group resourcefully utilised a glass chromatography column as a fixed bed reactor to perform the continuous flow synthesis of 3-((methyl(phenyl)amino)methyl)cyclohexanone (**15**, Scheme 6). The column was packed with glass beads to reduce the internal reactor volume before a mixture of silica gel and g-C_3_N_4_ was added and filled the voids between the glass beads. The column was wrapped in a blue LED array ribbon to complete the reactor. 2-Cyclohexenone (**13**) and 1.3 equivalents of the α-silylamine **14** were passed through the column at a flow rate of 1 mL⋅h^−1^, controlled by a plunger, resulting in a total residence time of 3 hours before the solution containing product **15** eluted to a collection flask. The setup was very effective and allowed for a gram-scale synthesis of the desired product with 85% yield, only a slight reduction of the batch-optimised yield of 95%. However, the 85% flow yield was achieved in only 3 hours, a significant reduction from 17 hours in batch. This report elegantly demonstrates the significant enhancement of heterogeneous photocatalysis that can be achieved when continuous flow is employed, and this demonstrates a simple and resourceful method to implement heterogeneous flow chemistry that is accessible without investing in expensive flow reactors and pumps.

**Scheme 7:**
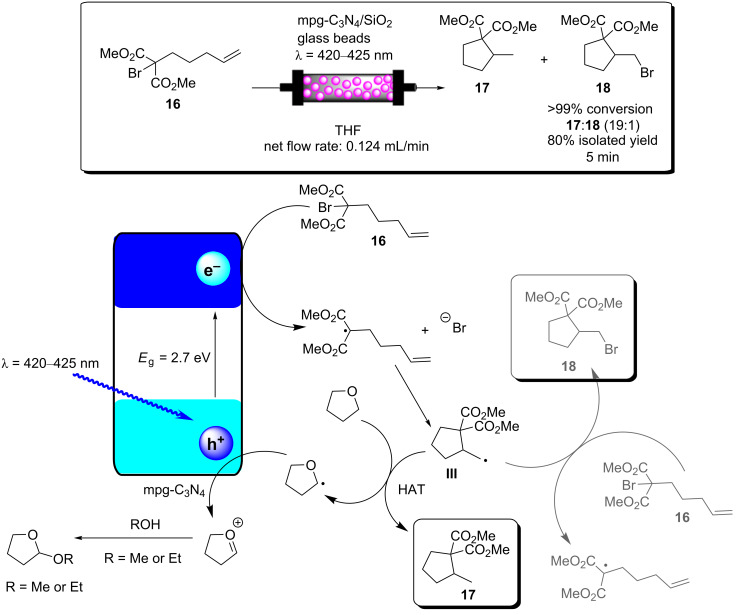
Proposed mechanism of the radical cyclisation of unsaturated alkyl 2-bromo-1,3-dicarbonyl compounds using mpg-C_3_N_4_ as a heterogeneous photocatalyst [237].

Blechert and co-workers reported a similar reactor setup for the radical cyclisation of the linear unsaturated alkyl 2-bromo-1,3-dicarbonyl compound **16** (Scheme 7) [237]. mpg-C_3_N_4_ was combined with silica gel and glass beads and immobilised in a length of transparent FEP tubing, which was arranged in a spiral, and fixed between two microscope slides to form a fixed bed-type reactor with a high surface-to-volume ratio. The substrate **16** in THF was supplied to the reactor by a syringe pump at 124 μL/min for an optimised residence time of 5 minutes. The reaction was previously reported using a [Ru(bpy)_3_]Cl_2_ photocatalyst in DMF with TEA as a sacrificial electron donor, which required 12 hours of irradiation for a high conversion on average [238]. During the optimisation, Blechert and co-workers found that by using THF as a solvent, they could achieve a high conversion at a reduced reaction time of 4 hours, and with no need for the TEA additive. They found that THF was able to undergo hydrogen atom transfer with the radical product precursor **III**, yielding the cyclic product **17**. The THF radical then reduces the h^+^_VB_ to complete the cycle, in place of TEA (Scheme 7) [237]. The brominated minor product, **18**, was suggested to result from the intermediate **III** abstracting a bromine atom from a second equivalent of **16**, or alternatively by **III** being oxidised by the h^+^_VB_ to form a cationic species that reacts with the liberated bromides from the initial reduction of **16**.

**Scheme 8:**
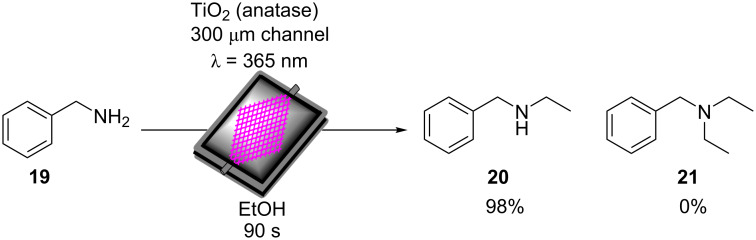
N*-*alkylation of benzylamine and schematic of the TiO_2_-coated microfluidic device [213].

The alkylation of benzylamine with various alcohols using a photocatalytic microfluidic device was reported by Matsushita and co-workers in 2007 and demonstrated the importance of the reactor design for the photocatalysis efficiency (Scheme 8) [213]. The microreactor comprised a quartz substrate with microchannels 40 mm long, 500 μm wide, and either 300, 500, or 1000 μm in depth. The bottom and side walls of the channels were coated in TiO_2_ via sol–gel spin coating, before being calcined at 770 °C for 2 hours in air. Platinum particles were deposited on the TiO_2_ layer via photodeposition from chloroplatinic acid using a mercury lamp irradiation source. The device was then covered with a transparent quartz window and sealed under pressure to yield a HPCat-coated microfluidic device. The photocatalysis experiments were performed using a syringe pump to flow a 1.0 mM solution of the amine substrate **19** (Scheme 8) in methanol, ethanol, or isopropanol under an inert nitrogen atmosphere. The flowing reaction solution was irradiated at 365 nm by UV LEDs in the microfluidic device for 30–150 seconds. The reaction was found to proceed rapidly and achieved 85% yield of *N*-ethylbenzylamine (**20**, Scheme 8) after 150 seconds in the 500 μm channel coated with Pt@TiO_2_. The reaction was found to proceed more efficiently with platinum-free TiO_2_-coated channels, achieving the same conversion after only 90 seconds. However, the authors did not suggest why the platinum particles were reducing the efficiency. The yield was intimately linked with the channel depth as after 90 seconds of irradiation, the platinum-free TiO_2_ 300 μm channel provided a 14% increase in yield over the 500 μm channel, and a 28% increase over the 1000 μm channel. This was attributed to the higher surface-to-volume ratio of the 300 μm channels. An analogous catalytic system, reported by Ohtani and co-workers, was performed in batch and required significantly longer reaction times of 5–10 hours to achieve a comparable conversion [239]. Interestingly, the batch system, over extended periods of irradiation, led to the production of the *N*,*N*-dialkylated benzylamine **21** (Scheme 8) with Pt@TiO_2_ particles suspended in an alcohol solution. No formation of the dialkylated product was observed with the microfluidic flow reactor, which the authors proposed was due to the much shorter irradiation times, providing an enhanced control over the product selectivity. The reaction was proposed to occur by the photoreduction of the alcohol solvent adsorbed at the TiO_2_ surface to generate H_2_ gas and a corresponding carbonyl compound via an alkoxy intermediate. The carbonyl compound subsequently condensed with the amine substrate to generate an imine, which was reduced to the secondary amine by the H_2_ produced in situ.

**Scheme 9:**
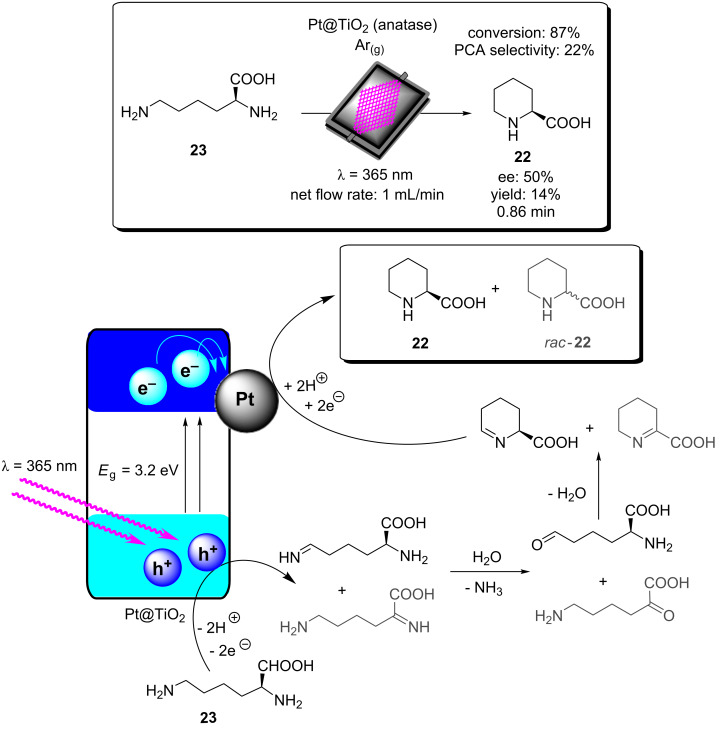
Proposed mechanism of the Pt@TiO_2_ photocatalytic deaminitive cyclisation of ʟ-lysine (**23**) to ʟ-pipecolinic acid (**22**) [240–241].

ʟ-Pipecolinic acid (**22**) was synthesised using a similar TiO_2_-modified microfluidic device (TMFD) by Kitamori, Kim, and co-workers (Scheme 9) [240]. The TMFD was fabricated from two pyrex glass substrates, with 700 μm wide and 3.5 μm deep channels. A 300 nm film of anatase TiO_2_ nanoparticles was immobilised to the channels using a sol–gel method and characterised by cross-sectional scanning electron microscopy. Photocatalytic activity of the device was initially assessed by the successful photodegradation of methylene blue dye. The photodeposition was used to deposit platinum nanoparticles as a reductive centre for the photoredox deaminative cyclisation of ʟ*-*lysine (**23**) to **22**. The TMFD could achieve near-full conversion of **23** (87%) after 52 seconds residence time, but with only 22% selectivity for both **22** and ᴅ*-*pipecolinic acid. This reduced the enantiopurity of the starting material to 50% ee, resulting in only 14% yield of **22**. Near-identical results were obtained by the group using commercial Pt@TiO_2_(P25) particles in a batch photoreactor after 1 hour, showing that the TMFD flow conversion rate was ≈70 times higher.

**Scheme 10:**
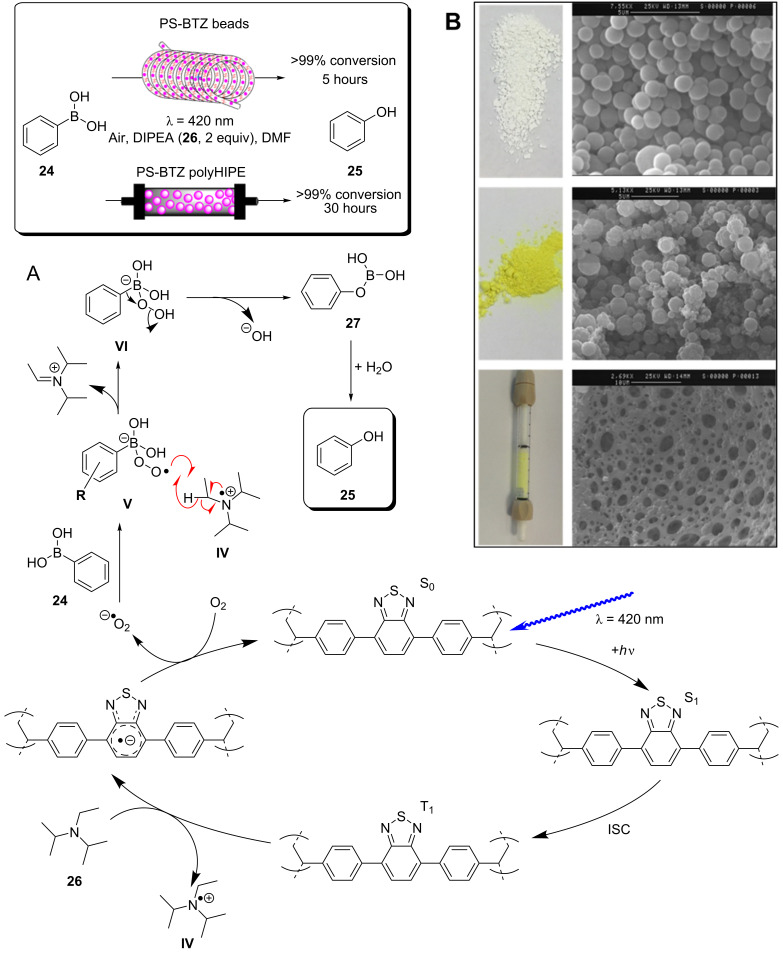
A) Proposed mechanism for the photocatalytic oxidation of phenylboronic acid (**24**). B) Photos and SEM images of (top) white polystyrene beads with no St-BTZ, (middle) yellow PS-BTZ beads, and (bottom) PS-BTZ polyHIPE monolith (photographed within a fixed bed reactor) [47,242]. Figure 10B was adapted from [47], Copyright 2017 American Chemical Society.

Vilela and co-workers reported an efficient continuous flow process for the oxidation of the aryl boronic acid **24** and pinacol esters to the corresponding phenol **25** using different polymer architectures with a common 2,1,3-benzothiadiazole (BTZ) photocatalyst core immobilised as a cross-linker in the polymer backbones. Polystyrene beads (PS-BTZ beads), gels, and a high-internal-phase emulsion-templated polymer monolith (PS-BTZ polyHIPE), all containing the same BTZ core, were synthesised to assess the effects of the morphology on the photocatalytic efficiency (Scheme 10) [47]. Phenylboronic acid (**24**), two equivalents of Hünig’s base (DIPEA, **26**) and 5 mol % of the HPCat (with respect to the immobilised BTZ unit) were irradiated at 420 nm under aerobic conditions in continuous flow. The mechanism was proposed to be the same as that previously reported by Xiao and co-workers, who used a homogeneous [Ru(bpy)_3_]Cl_2_ photocatalyst [242]. A single-electron transfer between the HPCat and **26** forms the radical cation **IV** (Scheme 10). The reduced HPCat is oxidatively quenched by O_2_, which generates superoxide radicals and returns the HPCat to the ground state. The superoxide radical proceeds to oxidise **24** to the corresponding radical intermediate **V**, followed by a hydrogen atom transfer with **IV** to form the peroxide adduct **VI**. This undergoes a rearrangement to eliminate a hydroxy group and yields the boric acid **27**, which is hydrolysed to yield the final product **25**.

The 5% poly(styrene-*co*-(4,7-distyrene-2,1,3-benzothiadiazole)) copolymer gel achieved a full conversion in a batch reactor within 18–72 hours for a variety of phenylboronic acid derivatives, substituted with electron-withdrawing or -donating groups. The PS-BTZ beads were also applied in the batch oxidation of **25** and showed a remarkable rate enhancement, reducing the reaction time to 10 hours. The PS-BTZ beads were then applied in a continuous flow slurry reactor, pumped through a Vapourtec E-series photochemical reactor coil, equipped with a 60 W 420 nm LED module. This further reduced the reaction time by 50%, achieving full conversion to **25** in just 5 hours. The PS-BTZ polyHIPE was applied as a HPCat for the oxidation of **24** under continuous flow conditions using a fixed bed reactor, but required 30 hours of irradiation to achieve a full conversion, less efficient than the 5% gel HPCat batch protocol. The authors found this poor efficiency disappointing, but noted that the material had been exposed to over 80 hours of intense irradiation prior to that reaction, which likely reduced the material’s performance.

As well as the significant enhancements of performing heterogeneous photocatalysis in a continuous flow, this work illustrates the importance of the morphological control in the design of photocatalytic materials and its effect on the photocatalysis efficiency. This is a significant advantage of organic polymer materials over metal oxide-based photocatalysts, which are far more restricted with respect to controlling the nanostructure and porosity.

**Scheme 11:**
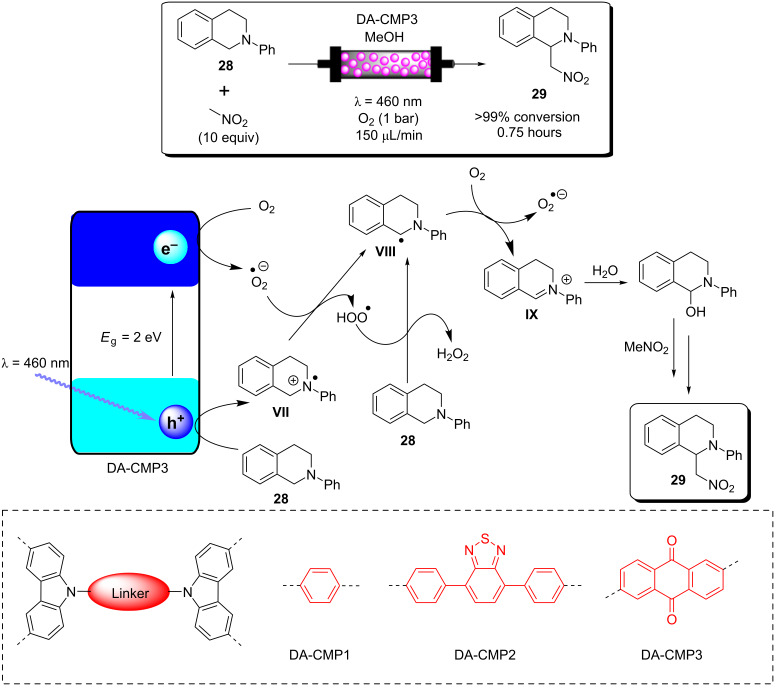
Proposed mechanism for the DA-CMP3 photocatalytic aza-Henry reaction performed in a continuous flow. The repeating unit structures of the three DA-CMPs are shown in the dashed box [243].

Liu and co-workers recently reported the synthesis of a series of carbazole-derived conjugated microporous polymers (CMPs) as HPCats for the aza-Henry reaction in continuous flow [243]. Biscarbazole monomers linked by either a benzene (DA-CMP1), 4,7-diphenylbenzo[1,2,5]thiadiazole (DA-CMP2), or anthracene-9,10-dione (DA-CMP3), were synthesised and then polymerised by an FeCl_3_-catalysed oxidative coupling to generate three donor–acceptor-type CMPs (Scheme 11). The CMPs were proposed to oxidise *N-*phenyltetrahydroisoquinoline (**28**) to an amine radical cation intermediate **VII**. Then, superoxide radicals generated by the e^−^_CB_ abstract a proton from **VII** to form the radical **VIII**. **VIII** is then further oxidised by molecular oxygen or an h^+^_VB_ to generate the iminium cation **IX**. **IX** can then react with a range of nucleophiles, such as nitromethane, to yield the C–H functionalised products in good yields (>70%). DA-CMP3 was found to be the most efficient HPCat for the aza-Henry reactions studied. DA-CMP3 was recycled 10 times without a loss of efficiency or selectivity in the conversion of **28** to **29**. The group applied DA-CMP3 in continuous flow using a fixed bed reactor and syringe pumps to concurrently pump the reaction mixture and oxygen through the catalyst bed, which was externally irradiated by a 460 nm LED module. Full conversion of **28** to the product **29** was obtained, and the reaction time was reduced by 25% through application of the flow reactor.

**Scheme 12:**
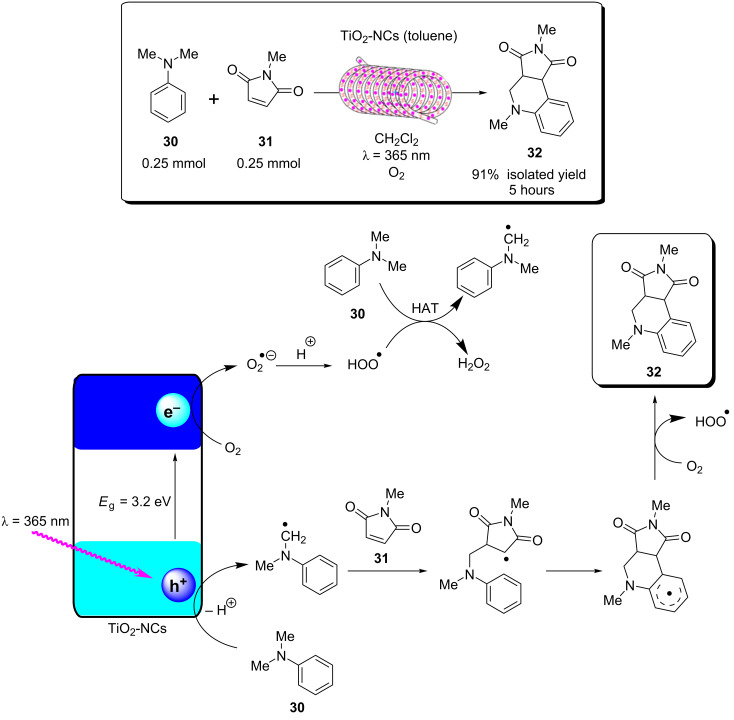
Proposed mechanism for the formation of the cyclic product **32** by TiO_2_-NC HPCats in a slurry flow reactor [244].

Kappe and co-workers reported a continuous flow synthesis and application of spherical and rod morphology TiO_2_ nanocrystals (TiO_2_-NCs) as HPCats. The TiO_2_-NCs were applied as HPCats for the reductive cyclisation of *N*,*N-*dimethylaniline (**30**) and methylmaleimide (**31**) to form the fused tricyclic tetrahydroquinoline **32** (Scheme 12) [244]. The morphology of the prepared TiO_2_-NCs could be controlled by varying the temperature of the stainless steel reactor coil placed in a GC oven, yielding rod-NCs at 250 °C and spherical-NCs at 300 °C. This was attributed to a transition between a kinetic and thermodynamic growth regime at the lower and higher tempratures, respectivley, facilitated by the extremely efficient heating of the coil flow reactor due to its high surface area. The large-scale production of monodispersed spherical TiO_2_-NCs HPCats was investigated and estimated at 57.6 g/day, with only a ca. 5-minute residence time required in the flow reactor. The authors suggested that the smaller spherical TiO_2_-NCs (5 ± 1 nm) with their high surface area would be efficient photocatalysts, and demonstrated this by flowing a suspension of the TiO_2_-NCs through a transparent PFA coil, irradiated by a UVA torch (≈365 nm) at 0.5 mL/min for 5 hours. As proposed, the spherical TiO_2_-NCs achieved a 91% isolated yield of **32**, whilst the rod TiO_2_-NCs only produced ≈54% under identical conditions [244]. The authors proposed the mechanism occurred as reported by Bian and co-workers, who used a [Ru(bpy)_3_]^2+^ photocatalyst for the same transformation [245]. The mechanism is shown in Scheme 12 and is overall very similar to the aza-Henry reaction mechanism discussed previously (Scheme 11).

**Scheme 13:**
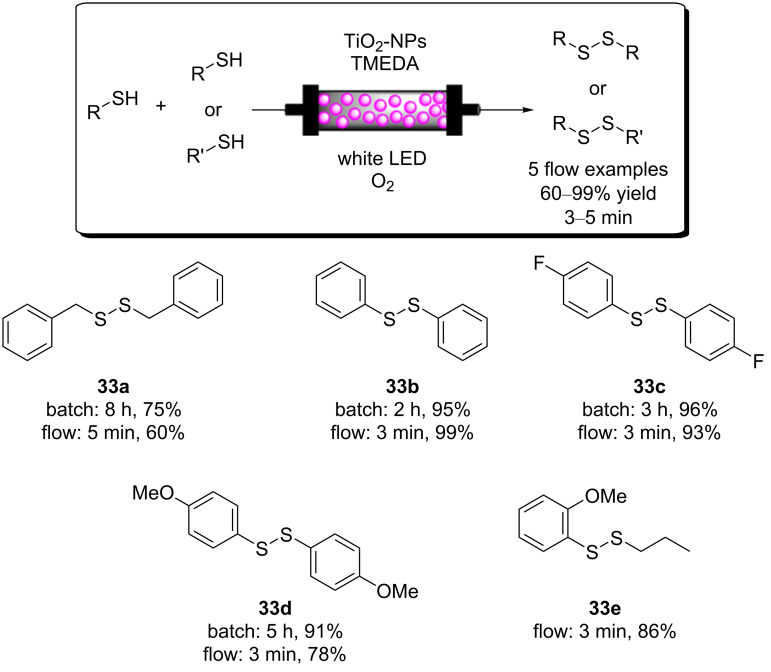
Reaction scheme for the photocatalytic synthesis of homo and hetero disulfides in flow and scope of the flow disulfide products **33a**–**e**, with yields and reactions times in batch and flow reactors given [209].

Noël and co-workers reported a procedure for the batch and flow heterogeneous photocatalysis synthesis of disulfides, a key functionality that controls the protein folding in nature and is used industrially in vulcanizing reagents [209]. The group utilised a fixed bed reactor packed with glass beads and TiO_2_ nanoparticles with 200 μL void space for the combined gas/liquid reactant phases (Scheme 13). The group had previously reported similar transformations using eosin Y as a homogeneous photocatalyst, and the TiO_2_ mechanism was proposed to be similar [246]. In the presence of a diamine base (TMEDA), TiO_2_ was activated towards visible light absorption and facilitated a proton-coupled electron transfer between the HPCat and the thiol substrates [246]. The resulting thiol radical couples with a thiolate to yield a radical anion, which is subsequently oxidised to form the desired disulfide products **33a**–**e**. The group demonstrated that the system could efficiently perform homothiol as well as the more elusive heterothiol couplings with a remarkable rate enhancement, reducing the reaction time required for high conversion from multiple hours in batch to 5 minutes or less in flow (Scheme 13) [209].

**Scheme 14:**
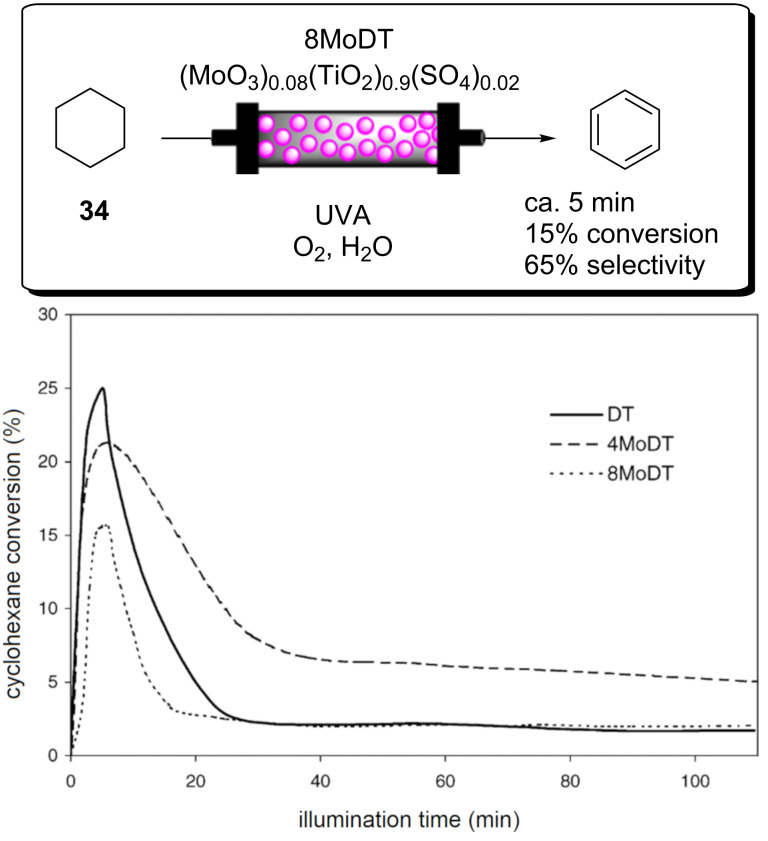
Reaction scheme for the MoO_x_/TiO_2_ HPCat oxidation of cyclohexane (**34**) to benzene. The graph shows the cyclohexane conversion over time using DT (anatase TiO_2_ with 2 wt % of sulphate), 4MoDT and 8MoDT (4.7 and 8.0 wt % MoO_3_ impregnated DT TiO_2_, respectively). Reprinted from [247].

Ciambelli and co-workers showed that a MoO*_x_*/TiO_2_ heterogeneous photocatalyst could oxidise cyclohexane (**34**) to benzene with high selectivity under UVA irradiation (Scheme 14) [247]. The gas phase photocatalytic oxidation was performed within an annular fixed bed photoreactor, surrounded by six 40 W UVA lamps, with an additional lamp in the central void space. The reactor was prepacked with quartz flakes, and the MoO*_x_*/TiO_2_ was deposited in situ by evaporating an aqueous slurry of the HPCat inside the reactor. The nitrogen gas flow was controlled by an MFC and bubbled through a solution of **34** to achieve a vapour concentration of 1000 ppm. This mixed with a flow of oxygen in N_2_ (1500 ppm) and H_2_O in N_2_ (1600 ppm) before entering the irradiated packed bed reactor. 15% conversion of **34** and 65% selectivity for benzene were achieved with an 8 wt % MoO*_x_*/TiO_2_ catalyst (8MoDT) after 5 minutes of irradiation. However, this rapidly declined to a steady state conversion of 2.3% after 15 minutes. Interestingly, when TiO_2_ or molybdenum supported on γ-alumina (Al_2_O_3_) were used as the HPCat independently, the activity was significantly lower, and the selectivity for CO_2_ was 100%, showing that the MoO*_x_*/TiO_2_ pairing was critical for the formation of the oxidative dehydrogenation products [247].

**Scheme 15:**
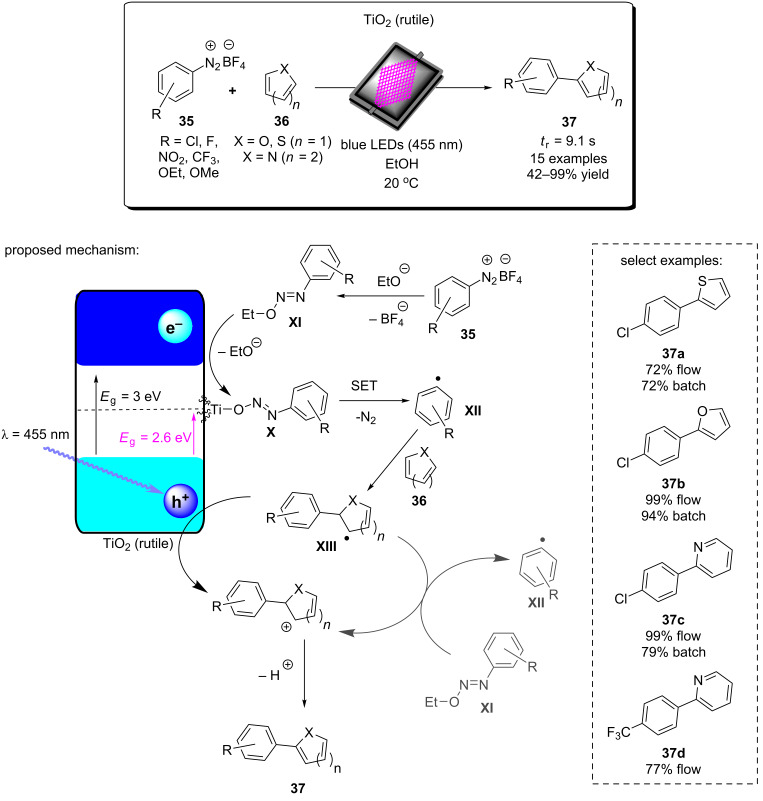
Proposed mechanism of the TiO_2_ HPC heteroarene C–H functionalisation via aryl radicals generated from aryl diazonium salts [94,248]. The select examples **37a**–**c**, prepared by using 4-chlorobenzenediazonium tetrafluoroborate with various heteroarenes, are shown with isolated yields for the flow and the batch reactor. The product **37d** was obtained from the long-term (180 min) continuous operation experiment, with a constant yield of 77%, assessed by ^19^F NMR spectroscopy using an internal standard.

Rueping and co-workers reported a continuous flow system for the C–H functionalisation of heteroarenes with aryl diazonium salts, utilising a TiO_2_-coated microfluidic falling-film microreactor (FFMR, Scheme 15) [248]. The group had previously studied the same reaction in batch and identified an interesting mechanism as the diazonium salt forms an adsorbed azoether species **X** on the TiO_2_ surface (Scheme 15), which sensitises the TiO_2_ to visible light absorption (λ_max_ = 480 nm) [94]. The aryl diazonium tetrafluoroborate salt **35** is in equilibrium with the azoether species **XI**, formed with the ethanol solvent. **XI** is then displaced by TiO2 surface oxides to form the adsorbed species **X**. The chemisorbed **X** lowers the band gap to approximately 2.6 eV, and excitation by 455 nm LEDs leads to a direct single-electron transfer from the valence band to **X**, resulting in an aryl radical **XII** and the loss of N_2_. The **XII** radical undergoes an addition with a heteroarene **36** (furan, thiophene, or pyridine) to give the radical **XIII**, which is oxidised by the TiO_2_ h^+^_VB_ to give the functionalised heteroarene product **37** [94]. The group produced the first example of a falling-film microstructured reactor (FFMR) with TiO_2_ coated to the stainless steel microchannels. The reactants were pumped via an HPLC pump to the FFMR at 0.5 mL⋅min^–1^ where the solution flow meets a countercurrent flow of N_2_ gas to facilitate mixing and self-draining [248]. The FFMR showed an impressive 6000-times enhancement in the specific reactor performance over the group’s previous batch procedure (batch: 5.4 × 10^−5^ mol⋅L^−1^⋅min^−1^, flow: 0.32 mol⋅L^−1^⋅min^−1^) [94,248]. The percentage yield for many of the compounds was also enhanced in the FFMR (see the selected examples **37a**–**c**, Scheme 15). The FFMR was used in a large-scale continuous operation experiment in which the reactor was irradiated for 180 minutes, and the conversion to the product **37d** was assessed by ^19^F NMR. The results showed a constant conversion of 77% across the 180-minute run, consistent with the scope evaluation and demonstrating the photostability of the reactor.

**Scheme 16:**
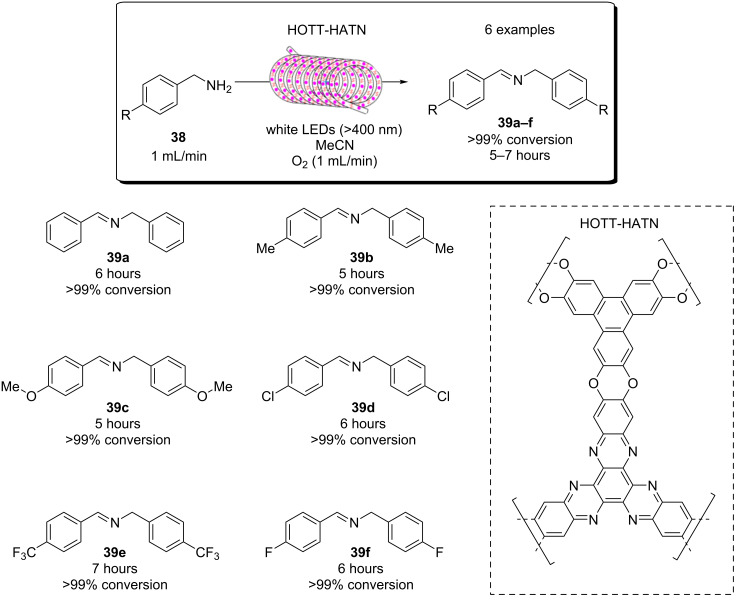
Scheme of the oxidative coupling of benzylamines with the HOTT-HATN HPCat and selected examples of the benzyl benzaldimine products **39a**–**f**. The structure of HOTT-HATN is displayed in the dashed box [249].

Xu, Vilela, and co-workers reported a trifunctional, nanoporous graphene-analogue material for heterogeneous visible light photoredox catalysis, Lewis base organocatalysis, and the removal of lead pollution from water [249]. The material was formed through an efficient metal-free synthesis using hexahydroxytriphenylene (HOTT) and hexachlorohexaazatrinaphthylene (HATN-Cl_6_) monomers that form a tritopic conjugated network via six base-catalysed S_N_Ar substitutions, yielding the desired HOTT-HATN polymer with 89% recovery. The material was found to be extremely efficient at removing lead from aqueous waste, reducing a 10 ppm/50 mL solution of Pb(NO_3_)_2_ to <15–20 ppb in 5 minutes, a level deemed acceptable by many global health organisations [249]. The material also functioned as an efficient organocatalyst for the Knoevenagel condensation of a range of benzaldehyde derivatives with malononitrile, achieving a full conversion in 3 hours on average and no loss in efficiency over 4 cycles. Finally, the group demonstrated the material’s efficiency as a HPCat in the aerobic oxidative coupling of the benzylamines **38** in flow (Scheme 16). The material was applied in a suspension reactor, pumped through a transparent coil, concurrently with a flow of O_2_ gas, and irradiated by a white or blue LED. The flow system achieved full conversion in approximately 6 hours with various benzaldehyde derivatives to the corresponding benzyl benzaldimines **39a**–**f** (Scheme 16). Electron-donating *para*-functional groups generally reduced the reaction time from 6 to 5 hours, and electron-withdrawing substituents conversely increased the reaction time to 7 hours. The reaction can plausibly occur via a photoredox or photosensitised singlet oxygen mediated mechanism. The authors tested the HPCat for the singlet oxygen production using α*-*terpinene (**44**) as a singlet oxygen trap (discussed in the next section), which proved successful. However, we would argue that this is not conclusive evidence that the mechanism occurs via singlet oxygen and not photoredox, as other reports tend to favour the photoredox mechanism for TiO_2_ and transition metal-based photocatalysts [250–253]. Additionally, photoredox and photosensitisation ability are not mutually exclusive, so the positive result for singlet oxygen production does not eliminate the possibility of a photoredox mechanism in this reaction, or the two pathways could occur in tandem. We suggest more conclusive evidence is required to elucidate the true mechanism.

**Scheme 17:**
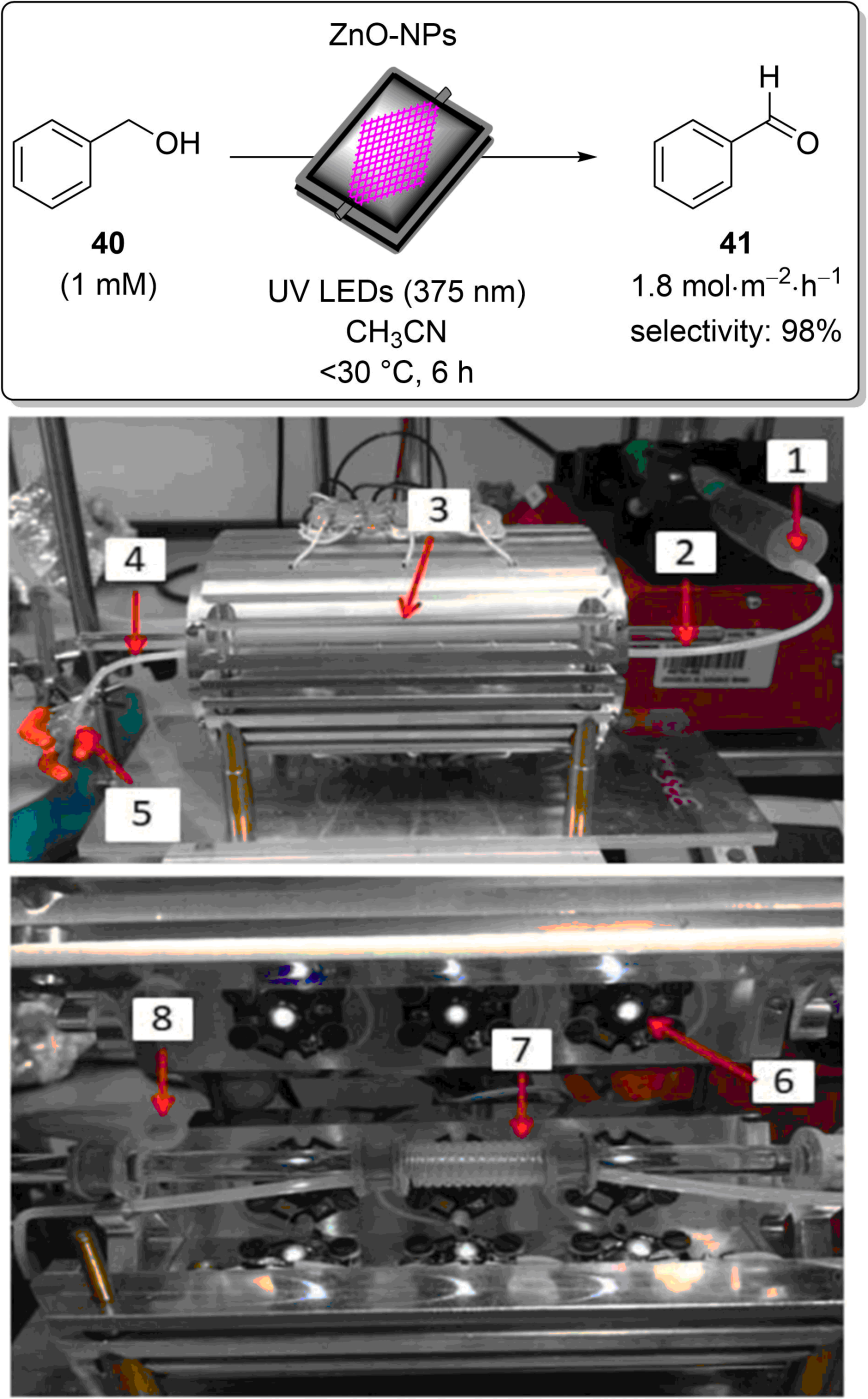
Photocatalysis oxidation of benzyl alcohol (**40**) to benzaldehyde (**41**) in a microflow reactor coated with ZnO nanoparticles through ultrasonic-assisted deposition. Images of the microflow photoreactor set-up: (1) syringe infusion pump, (2) teflon tube, (3) metal covering containing the LED and the photocatalytic microreactor, (4) teflon tube, (5) sample collector, (6) UV LED, (7) ZnO-US-FEP microtube, and (8) air cooling tube. Reprinted with permission from [254], Copyright 2019 The Royal Society of Chemistry.

Colmenares, Nair, and co-workers recently reported the design of a microflow reactor coated with commercial ZnO nanoparticles (ZnO-NPs) as HPCats for the selective oxidation of benzyl alcohol (**40**) to benzaldehyde (**41**, Scheme 17) [254]. The microflow reactor was composed of a transparent fluorinated ethylene propylene (FEP) tubing coil (0.8 mm internal diameter), which had ZnO-NPs deposited in a flow channel surface through an ultrasound deposition treatment method [254]. The ZnO-NP-coated reactor coil was housed in a cylindrical metal casing with an array of UV-LEDs irradiating at 375 nm with a power density of 16.6 W⋅m^−2^. Solutions of **40** (1 mM) in either acetonitrile or water were pumped through the reactor at 0.053 mL⋅min^−1^ for a total of 6 hours. The acetonitrile solution in the flow reactor system achieved a peak selectivity of 98% for **41** after 15 minutes of initiating the experiment, and a specific conversion rate of 1.8 mol⋅m^−2^⋅h^−1^. The specific conversion rate of **40** was more than 2 orders of magnitude higher using water as solvent, peaking at 288 mol⋅m^−2^⋅h^−1^. However, the selectivity for **41** was reduced significantly to <15%. For both solvents, the microflow reactor had significantly higher specific conversion rates than the analogous batch photochemical reactions, which only achieved 0.07 mol⋅m^−2^⋅h^−1^ and 0.6 mol⋅m^−2^⋅h^−1^ in acetonitrile and water, respectively. The coated microflow reactor system was recycled over 5 runs to test the stability of the ZnO-NPs deposited in the FEP tubing. They reported a negligible decrease in the specific conversion rate for both solvent systems over 30 hours of testing, although selectivity for **41** decreased over the 5 cycles by approximately 10%. The group suggested that this was due to byproducts and unreacted starting material from previous runs adsorbing to the ZnO-NP surface. The photocatalysis reaction samples were analysed by energy-dispersive X-ray fluorescence (EDXRF) spectroscopy, which found no traces of Zn^2+^ ions in the solutions. This, in combination with the recycling experiments led the group to conclude that the ultrasonic deposition was an effective and stable method for the deposition of HPCat nanoparticles [254].

Earlier this year, Colmenares, Pradhan, and co-workers subsequently published a similar report utilising commercial TiO_2_(P25) and their own sol–gel-synthesised TiO_2_ nanoparticles (TiO_2_-SG) as HPCats for the oxidation of **40** to **41** [255]. The TiO_2_ materials were immobilised by the ultrasonic deposition on the surface of perfluoroalkoxy alkane (PFA) tubing using the same method developed for the ZnO-NPs under theoretically optimised conditions obtained from Design Experiment software [255]. Coated microflow reactors were produced both with and without an ultrasound treatment for comparison, and they reported that the ultrasound-assisted deposition reactors had approximately 50% higher quantities of immobilised TiO_2_. The TiO_2_(P25) and TiO_2_-SG nanoparticles were first tested and compared for the oxidation of **40** in a batch photoreactor for 60 minutes. TiO_2_(P25) displayed much higher conversion rate relative to TiO_2_-SG, which became inactive within the first 20 minutes. However, the selectivity of TiO_2_-SG for **41** was >80% for the duration of the experiment, significantly higher than TiO_2_(P25), which displayed a steady state selectivity of approximately 28%. Interestingly, when immobilised in microflow reactors, the HPCat efficiencies were reversed with TiO_2_-SG achieving approximately double the conversion of TiO_2_(P25). The selectivity of TiO_2_-SG remained consistent with the batch reactor, however, the selectivity of immobilised TiO_2_(P25) was dramatically enhanced to >75%. However, both microflow reactors displayed a fixed conversion across the 60 minutes of the reaction, which was achieved within the first 10 minutes. Overall, the TiO_2_-SG microtube flow reactor achieved the highest yields of **41** with a 6 μmol⋅m^−2^⋅min^−1^ specific conversion rate and 87% selectivity. This was an inferior result to their previously published ZnO-NPs system. However, the study showed the versatility of the ultrasonic-assisted deposition methodology and provides an interesting example of selectivity enhancement through flow chemistry [255].

#### 4.2 Heterogeneous energy transfer catalysis in flow

**Figure 17:**
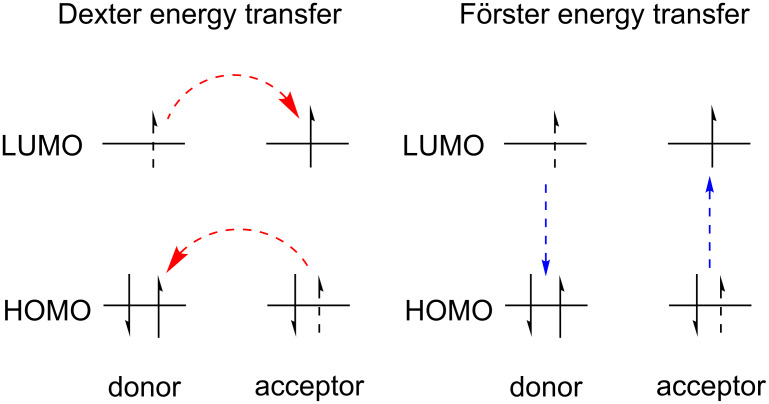
Mechanisms of Dexter and Forster energy transfer.

Photosensitisation, or energy transfer catalysis, is a process by which the photon energy absorbed by a HPCat is transferred to a substrate through electronic transitions and without changing the redox states of either species. This can occur via two main mechanisms; Dexter and Förster energy transfer (Figure 17). Dexter energy transfer is a simultaneous transfer of two electrons between an excited photocatalyst and a ground state acceptor. The photocatalyst-excited electron transfers to the LUMO of the ground state acceptor, whilst an acceptor HOMO electron simultaneously transfers to the photocatalyst HOMO vacancy. This produces no net change in the redox states but causes the substrate to enter an electronic excites state and returns the HPCat to its ground state, effectively transferring energy between the two species. The Dexter mechanism is restricted to short distances as the donor and acceptor must have an orbital overlap for this to occur [256].

Förster resonance energy transfer (FRET) involves no intermolecular electron exchange and occurs over very large separations of 30 to 100 nm [256–257]. In this process, an excited state energy donor and a ground state acceptor transfer energy through resonance of their electronic emission and absorption frequencies and is largely dictated by Frank–Condon parameters (Figure 17) [256]. FRET is therefore limited by the requirement that the energy donor’s emission spectrum has an overlap with the energy acceptor’s absorption spectrum.

Once an acceptor has been photosensitised to an electronic excited state, changes in the molecular polarisation, bond strengths, or spin multiplicity enables unique chemical reactivity and has been utilised for pericyclic reactions, isomerisations, and atom abstraction reactions [258]. Photosensitisation was the basis of most photochemical organic syntheses prior to the advent of photoredox catalysis [259]. A very detailed tutorial review on energy transfer photocatalysis by Glorius and co-workers is recommended for further reading [258].

**Scheme 18:**
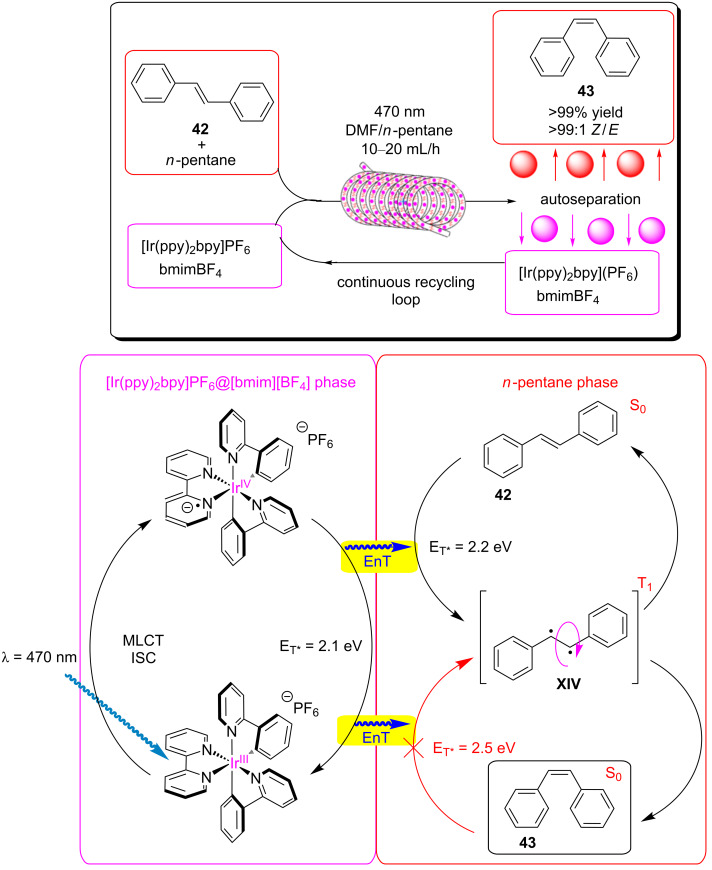
Continuous flow process for the isomerisation of alkenes with an ionic liquid-immobilised photocatalyst [260].

An interesting example of heterogeneous energy transfer photocatalysis was reported by Rueping and co-workers in which an iridium complex photocatalyst was immobilised in an ionic liquid phase that was immiscible with the organic substrate phase. The system was applied in flow for the isomerisation of various *trans*-aromatic alkenes to the corresponding *cis-*alkene (Scheme 18) [260]. The novelty of this study was that the molecular photocatalyst was immobilised in an ionic liquid acting as a liquid-phase HPCat due to its immiscibility with the reaction-substrate organic phase. As the biphasic flow system has a large interfacial surface area relative to a batch reaction, the heterogeneous energy transfer across the interface was efficient, and the isomerisation occurred in almost quantitative yield and >99:1 *Z*:*E* ratio for most of the *trans*-alkenes studied. *E*-stilbene (**42**) is triplet-sensitised by the [Ir(ppy)_2_(bpy)]PF_6_ photocatalyst, as shown in Scheme 18. The triplet state of the stilbene **XIV** is a diradical and has a lower bond order, permitting the free rotation of the alkene bond before relaxing back to the singlet state in either the *E*- or *Z*-isomeric form. The group proposed that the high efficiency of the reaction was due to the differing triplet state energies (*E*_T*_) of *E*- and *Z*-stilbene (**42** and **43**), which permits the triplet sensitisation of *E-*stilbene (**42**, *E*_T*_ = 2.2 eV) by the [Ir(ppy)_2_(bpy)](PF_6_) photocatalyst (E_T*_ = 2.1 eV) as the triplet state energies are similar. However, the *Z-*stilbene (**43**, *E*_T*_ = 2.5 eV) triplet state energy is too high for an energy transfer to occur and makes the isomerisation irreversible, leading to high yields and selectivity of the desired *Z*-isomer. The differing density of the two phases provided an automatic separation of the catalyst and product in the collection vessel, permitting a continuous recycling loop of the photocatalyst ionic liquid phase [260].

**Scheme 19:**
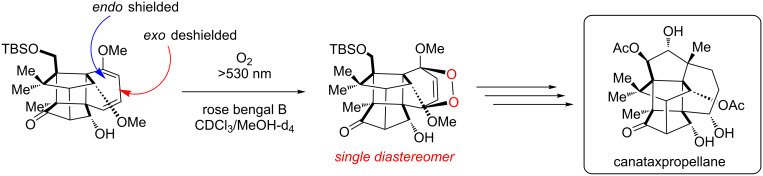
Singlet oxygen synthetic step in the total synthesis of canataxpropellane [265].

Singlet oxygen is a reactive oxygen species formed by a photosensitised electronic transition in which the unpaired π-electrons of triplet ground state molecular oxygen (^3^Σ_g_^−^) are paired with the inversion of one electron’s angular momentum to yield a singlet excited state (^1^Δ_g_). There is a low energy barrier of 94 kJ/mol to achieve the transition, but the direct transition is forbidden by quantum mechanical spin selection rules, and hence requires triplet photosensitisation to occur [261]. Singlet oxygen is a unique reactant for the oxidation of organic compounds and has been frequently applied in the total synthesis of natural products and pharmaceutical agents [262–263], including the Nobel Prize-winning antimalarial drug artemisinin (**49**) [132,264]. A photosensitised singlet oxygen Alder-ene [4 + 2] cycloaddition was a key step in the total synthesis of canataxpropellane (Scheme 19), reported recently by Gaich and co-workers, described as one of the most complex natural products ever isolated [265–266].

**Scheme 20:**
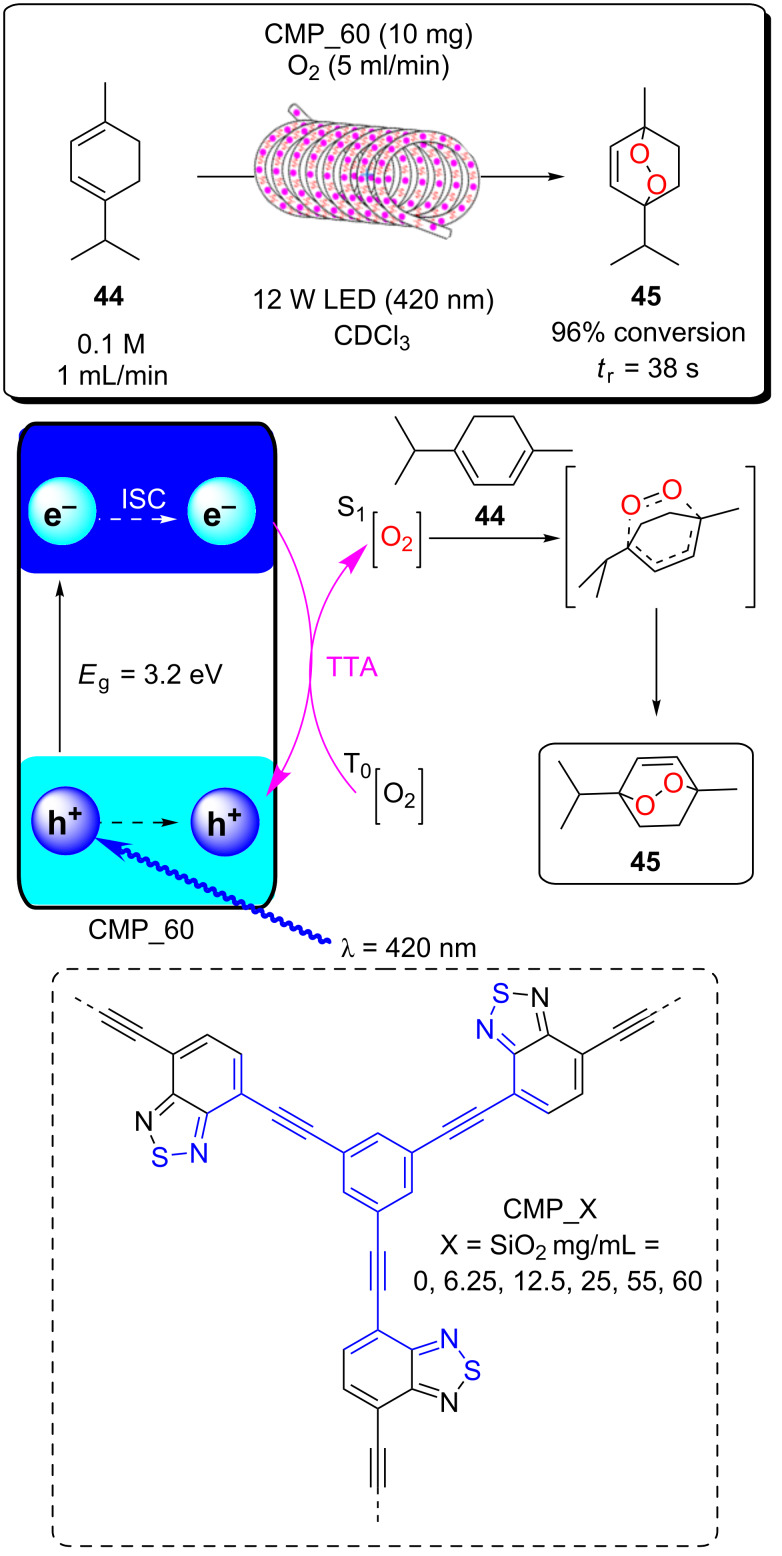
Scheme and proposed mechanism of the singlet oxygen photosensitisation by CMP_X HPCats, with the structure of CMP_X CPP displayed in the inset. The section of the polymer structure in blue represents one repeating unit. ISC = intersystem crossing [267].

Vilela and co-workers reported a continuous flow process to generate photosensitised singlet oxygen for the oxidation of α*-*terpinene (**44**) to ascaridole (**45**) using a conjugated microporous polymer (CMP) templated with silica nanoparticles (SiO_2_NPs) as a porogen (Scheme 20) [267]. The CMP was synthesised via a Sonogashira palladium-catalysed cross-coupling of 4,7-dibromobenzo[*c*]-2,1,5-thiadiazole and 1,4*-*diethynylbenzene using various concentrations of SiO_2_NPs (6.25, 12.5, 25, 55 and 60 mg⋅mL^−1^) as a templating agent to control the porosity and surface area of the resulting CMP_X (X = SiO_2_NPs mg⋅mL^−1^). The SiO_2_NPs could be chemically removed from the polymer matrix through treatment with aqueous NH_4_HF_2_, without damaging the polymer material. The surface area and total pore volume of the native, non-templated CMP_0 could be more than doubled using a SiO_2_NPs concentration of 60 mg⋅mL^−1^. The polymers were suspended in a 0.1 M solution of **44** in CDCl_3_ and irradiated with 420 nm photons in a slurry flow reactor, comprised of a syringe pump and FEP tubing within an enclosed reactor lined with tin foil. The templating strategy had a significant influence on the photosensitisation efficiency, increasing the conversion of **44** from 26% with CMP_0 to 96% with CMP_60 in a single pass of the reactor. Interestingly the increase in SiO_2_NP concentration from 0–60 mg⋅mL^−1^ led to an exponential increase in the surface area and a linear increase in the pore volume. However, the photosensitisation enhancement was non-linear. CMP_6.25 raised the conversion from 26% to 81%, but concentrations of 12.5, 25, and 55 mg⋅mL^−1^ had little effect and provided conversions slightly lower than what CMP_6.25 achieved. The authors postulated the possible influence of the dispersibility and effective surface area on the efficiency. The CMPs generate singlet oxygen through triplet–triplet annihilation energy transfer, involving a CMP triplet exciton and triplet ground state molecular oxygen transfer energy, returning the CMP to its ground state and producing singlet oxygen. Singlet oxygen then undergoes a concerted but asynchronous Alder-ene [4 + 2] cycloaddition with **44** to yield the endoperoxide product **45** [268].

**Scheme 21:**
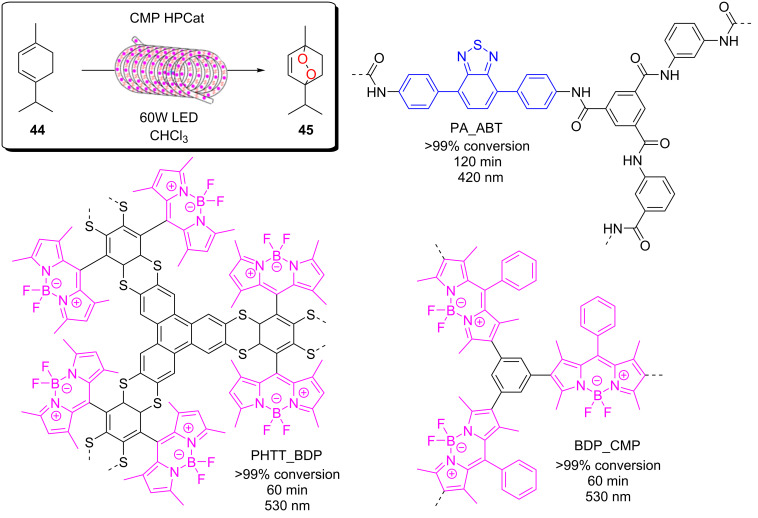
Structures of CMP HPCat materials applied by Vilela and co-workers for the singlet oxygen photosensitisation in continuous flow [131,269].

Following this, Vilela and co-workers have reported a number of different CMP materials for the photosensitisation of singlet oxygen in continuous flow, including BODIPY CMPs (BDP_CMP, PHTT_BDP) [131] and a water-dispersible polyamide-based BTZ CMP (PA_ABT, Scheme 21) [269]. All of these were applied for the photosensitisation of singlet oxygen and the subsequent oxidation of **44** to **45** using a slurry reactor system and a commercial flow reactor (Vapourtec Ltd. E-Series Photochem) equipped with a reactor coil, irradiated by a 60 W LED module of various monochromatic wavelengths.

**Scheme 22:**
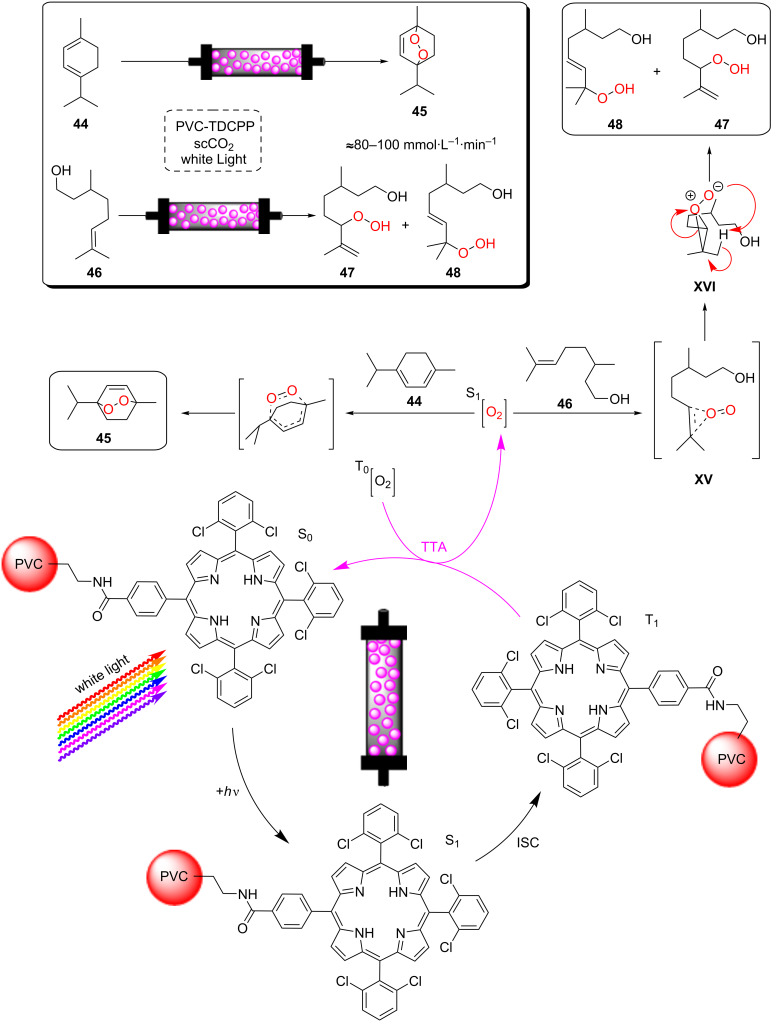
Polyvinylchloride resin-supported TDCPP photosensitisers applied for singlet oxygen photosensitisation in scCO_2_. The mechanism of the photosensitisation of singlet oxygen and the subsequent oxidation of citronellol (**46**) and α-terpinene (**44**) [271].

Poliakoff, George, and co-workers have pioneered the use of supercritical CO_2_ (scCO_2_) as a reaction medium for photosensitised singlet oxygen production in flow, including using immobilised molecular photosensitisers (Scheme 22) [132,270–272]. Oxygen is completely miscible with scCO_2_, which eliminates the mass transport limitations of oxygen to the photosensitiser but does require specialised equipment and operation at high pressure (10–18 MPa) [272]. They first reported a series of porphyrin, xanthene, and [Ru(bpy)_3_]^2+^ photosensitisers immobilised to polymer resins, polyvinyl chloride films, and SiO_2_ aerogels as support materials [271]. Many of the materials produced were efficient singlet oxygen photosensitisers in the scCO_2_ flow system, driving the oxidation of **44** and citronellol (**46**, Scheme 22) but suffered from rapid photobleaching or leaching of the immobilised photosensitiser. They identified a 5-(4-carboxyphenyl)-10,15,20-tris(2,6-dichlorophenyl)porphyrin (TDCPP-COOH) photosensitiser covalently immobilised to aminopoly(vinyl chloride) resins via an amide bond as the optimal HPCat for the system, maintaining high space–time yields (≈80–100 mmol⋅L^−1^⋅min^−1^) for both reactions over 320 minutes of continuous operation. The HPCat was immobilised in a transparent, fixed bed sapphire tube reactor for high-pressure operation (18 MPa) and irradiated by two arrays containing four 1000 lumen white LEDs mounted on aluminium heat sinks. The conversion of **44** was monitored by on-line GLC analysis, but the conversion of **46** to the hydroperoxides **47** and **48** was monitored manually by ^1^H NMR spectroscopy due to the thermal instability of the products. The oxidation of **46** occurs by a Schenk-ene group transfer pericyclic reaction (Scheme 22). The mechanism is proposed to occur stepwise, first forming the exciplex **XV**, which leads to the perepoxide intermediate **XVI**. The **XVI** species then undergoes the ene pericyclic rearrangement by abstracting a proton to yield the hydroperoxide products **47** and **48**. The true depth of study required for the early elucidation of this mechanism is surprising, and was reviewed in detail by Clennan [273].

**Scheme 23:**
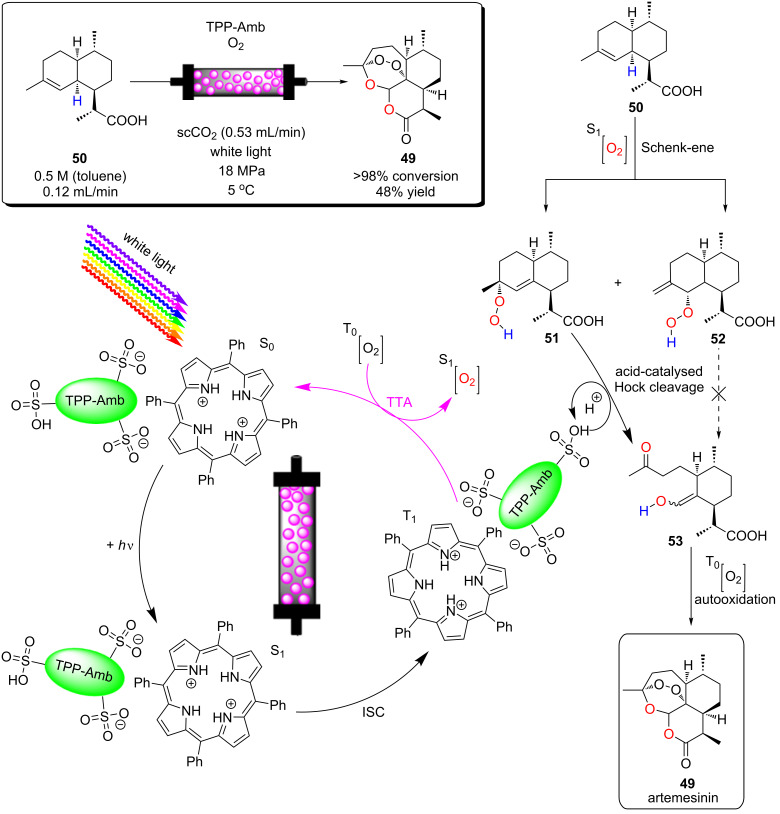
Structure of the ionically immobilised TPP photosensitiser on amberlyst-15 ion exchange resins (TPP-Amb) and proposed mechanim of the tandem singlet oxygen photosensitisation and Brønsted acid catalysis in the flow synthesis of artemesinin (**49**). Note that the acid-catalysed Hock cleavage step is independent from the TPP electronic state, as this could be misinterpreted from the mechanism shown [132].

The same group later reported a bifunctional heterogeneous photosensitiser and Brønsted acid catalyst for the continuous flow synthesis of artemisinin (**49**, Scheme 23) [132]. They had intended to use an amberlyst-15 ion exchange resin (sulfonated cross-linked-polystyrene resins) as a heterogeneous Brønsted acid catalyst within the same sapphire tube reactor described above, and a homogeneous tetraphenylporphyrin (TPP) photosensitiser within the scCO_2_ phase to perform the final steps of the artemisinin (**49**) semisynthesis, simultaneously within the same reactor [274]. The authors were surprised to find that the TPP had become immobilised to the resins, indicated by a colour change from the grey resins to dark green. They reported that the TPP photosensitiser had been protonation by the amberlyst-15 resins, resulting in a strong electrostatic immobilisation and formation of the bifunctional TPP-Amb heterogeneous catalyst. TPP-Amb could efficiently generate singlet oxygen for the Schenk-ene oxidation of dihydroartemisinic acid (**50**) to the hydroperoxides **51** and **52**. The TPP-Amb then utilises its Brønsted acid catalyst ability to drive the Hock cleavage of **51** to form the intermediate **53**. This undergoes an autooxidation by molecular oxygen and a subsequent cyclisation cascade to form **49**, which is obtained directly from the flow reactor outlet in 50% yield (Scheme 23) [132].

**Scheme 24:**
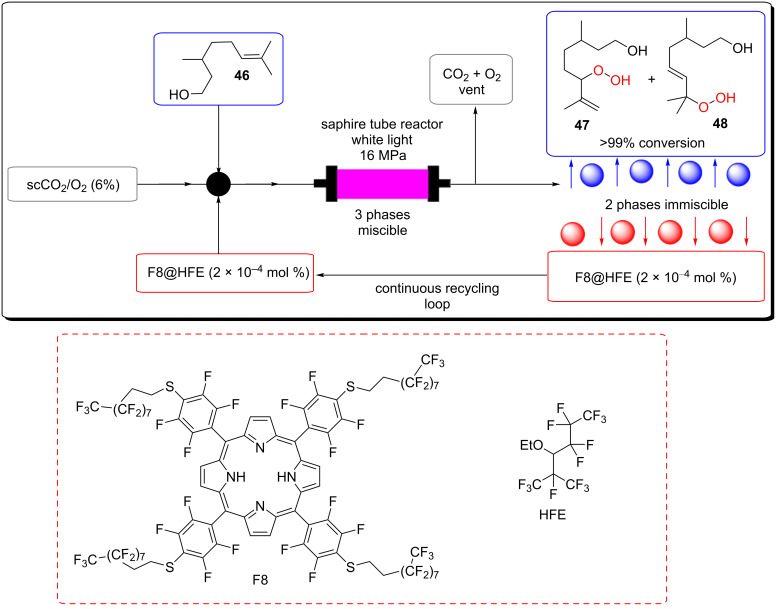
Photosensitised singlet oxygen oxidation of citronellol (**46**) in scCO_2_, with automatic phase separation of the fluorous solvent and photosensitiser for a continuous operation [272].

In a similar strategy to that reported by Rueping and co-workers discussed at the beginning of this section [260], Bourne, George, and co-workers had previously reported singlet oxygen photosensitisation in a scCO_2_ flow reactor system, using what we will describe as a “pseudo-HPCat” [272]. The group produced a highly fluorophilic porphyrin photosensitiser derived from 5,10,15,20-tetrakis(pentafluorophenyl)porphyrin (TPFPP) with perfluoroalkyl chains (F8) to enhance the solubility in scCO_2_ and a HFE-7500 fluorous solvent (Scheme 24). The organic substrate was pumped neat to a three-way mixer where it met a flow of scCO_2_/O_2_ (6%) and a flow of the F8/HFE fluorous phase (Scheme 24). In the presence of scCO_2_ at 18 MPa, the three phases became a miscible single phase, which passed through the irradiated sapphire tube reactor before being depressurised and collecting the liquid outflow. As the mixture is depressurised, excess CO_2_ and oxygen returns to a gaseous phase and is vented into the atmosphere. The remaining product and fluorous phases become immiscible and separate, aided by an ultrasonic bath and some MeOH additive to reduce the viscosity of the organic products. This allowed the F8@HFE to be continuously recycled by pumping the fluorous phase directly from the base of the collection vessel, achieving a homogeneous reaction system free of mass transport limitations whilst retaining the separation benefits associated with a HPCat [272]. They found the turnover number of this system in a single pass was 27 times greater than their previous report using a TPFPP photocatalyst with dimethyl carbonate as a solubilising additive [270]. 12 mL of the F8@HFE fluorous phase could be recycled ten times to produce 240 mL of the product before photocatalyst leaching to the organic phase became problematic and reduced efficiency. The volatility of HFE-7500 led to only 55% recovery per cycle, and HFE had to be continuously replenished during the recycling process to account for this. However, the authors suggested that a more sophisticated depressurising set-up could have prevented this issue.

**Scheme 25:**
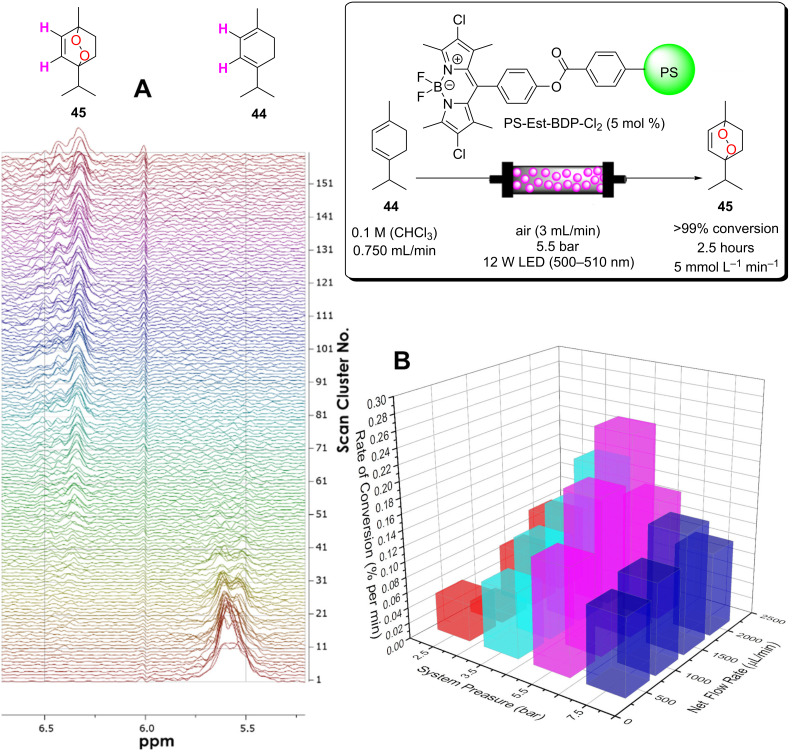
Schematic of PS-Est-BDP-Cl_2_ being applied for singlet oxygen photosensitisation in flow. A) Pseudo-2D ^1^H NMR trace from the in-line monitoring of the **44** and **45** alkene proton signals during the reaction. B) Graph of the conversion per minute at varied pressure and flow rate during the process optimisation. Adapted from [63], published by Springer Nature under a Creative Commons Attribution 4.0 International License http://creativecommons.org/licenses/by/4.0/.

As mentioned in Section 2.3, our group recently reported a polymer-supported BODIPY photosensitiser, synthesised and applied in continuous flow for the generation of photosensitised singlet oxygen [63]. The BODIPY was immobilised through a non-conjugated position of the molecule so that the photophysical properties would be comparable to the homogeneous analogues of the immobilised species. Interestingly, we found that despite being non-conjugated to the chromophore, the linker strategy enhanced the photosensitisation ability of the molecule by reducing a photoinduced electron transfer (PET) between the BODIPY core and the meso*-*substituted linker. Chlorinated BODIPY derivatives were serendipitously isolated from an unexpected side reaction and provided an even greater reduction in PET and a suspected enhancement in the triplet state quantum yield due to the chlorines providing a mild heavy atom effect. The oxidation of α*-*terpinene (**44**) was used as a model reaction to assess the photosensitisation efficiency of the material. An in-line ^1^H NMR benchtop spectrometer was employed to optimise the pressure and flow rate through the irradiated fixed bed reactor containing the HPCat (Scheme 25). It was found that an optimal pressure of 5.5 bar made the biphasic flow of air and the liquid reagents in CHCl_3_ miscible, which greatly enhanced the reactivity by increasing the concentration of dissolved O_2_ and improving mass transport. The polystyrene-supported, ester-linked BODIPY (PS-Est-BDP) material was irradiated for 24 hours while continuously cycling the reaction mixture through the fixed bed. The same material was recycled four times without loss of photosensitisation ability, albeit with low yields. The material was postsynthetically modified in flow for a second time in an attempt to increase the photosensitisation efficiency. *N-*chlorosuccinimide in hexafluoroisopropanol solvent was pumped through the immobilised PS-Est-BDP material to chlorinate the chromophore and form PS-Est-BDP-Cl_2_ (Scheme 25), the immobilised equivalent of the optimal homogeneous photosensitser. This, in combination with the optimised conditions established by the in-line ^1^H NMR, resulted in a 24-fold enhancement in efficiency compared to the initial material and conditions and produced a HPCat flow reactor system that was more efficient than the equivalent homogeneous batch reaction (>99% conversion, 2.5 hours in flow, 3 hours in batch). The chlorinated PS-Est-BDP-Cl_2_ resin’s efficiency rapidly declined to ≈65% after 5 cycles (12.5 hours), implicating a higher singlet oxygen photosensitisation efficiency with a faster deactivation or cleavage of the photocatalyst from the resin.

3D-printed reaction vessels that incorporate an active catalyst within the surface of the vessel have been pioneered by the group of Cronin to produce the bespoke reaction vessels for organic synthesis [275–277]. This provides an interesting approach for the fabrication of flow reactors with a complete freedom of design and the potential to print intricate static mixing architectures and flow channels in all three dimensions. 3D printing flow reactors embedded with a photocatalyst is a source of challenge and opportunity, as printing a photocatalytic monomer via stereolithographic 3D printing (light-initiated polymerisation) has some intuitive compatibility concerns.

**Scheme 26:**
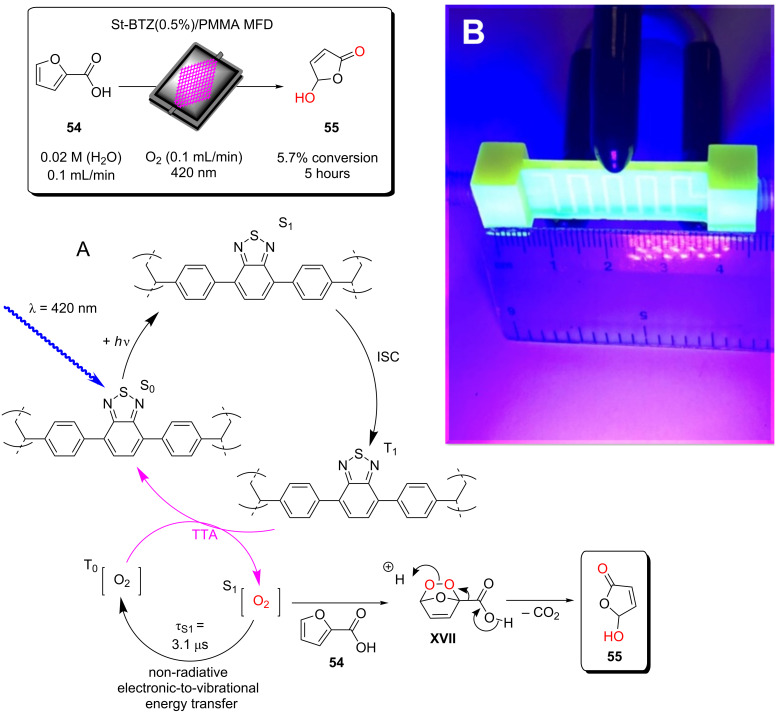
Reaction scheme of the singlet oxygen oxidation of furoic acid (**54**) using a 3D-printed microfluidic device containing a photocatalyst monomer additive [278]. A) Proposed oxidation mechanism [280]. B) Image of the MFD in operation, reprinted from http://www.VilelaLAB.co.uk with permission.

Xuan, Vilela, and co-workers recently published the first example of this through the additive manufacturing of a HPCat microfluidic device. The previously published St-BTZ monomer was incorporated into a commercial PMMA resin (Clear FLGPCL02, Formlabs Inc.) to obtain a St-BTZ (0.5 wt %)/PMMA resin mixture [47,278]. The additive manufactured resin mixture was applied in a commercial stereolithograph apparatus (SLA) 3D printer (Form 1+, Formlabs Inc.), and the printing process was optimised to account for the photocatalyst’s absorption of the SLA 3D printer’s 405 nm laser, which initiates the free-radical polymerisation of the resin. The microfluidic device was designed using computer-aided design software and fabricated with a 1 mm channel diameter, 500 micron wall thickness, and a total reactor volume of 0.1 mL. The device was applied for the photosensitisation of singlet oxygen, using the oxidation of 2-furoic acid (**54**) to 5-hydroxy-5*H*-furan-2-one (**55**) in water as a model reaction (Scheme 26). The oxidation mechanism is similar to **44** and begins with an Alder-ene [4 + 2] cycloaddition. However, the intermediate endoperoxide **XVII** then decarboxylates to liberate CO_2_. The subsequent protonation yields the product **55**.

The device was tested for 5 hours with continuous cycling of the reaction mixture and showed full selectivity of the lactone product but only obtained ≈6% conversion. The low conversion can be attributed to a few factors: (i) Water was used as a solvent for a compatibility with the printed polymer, which provides a very short lifetime of singlet oxygen (ca. 3.1 μs) before non-radiatively decaying to the ground state [279]. (ii) The dispersion of the photocatalyst throughout the polymer material may have resulted in significant unproductive excitation in the bulk and external surfaces of the microfluidic device, reducing the productive excitation of photocatalyst in the flow channel surfaces. The authors stated that work is ongoing to develop solvent-resistant resins and intricate reactor designs that will help to overcome these issues [278].

#### 4.3 Photocatalytic water purification in flow

Approximately 4 billion people globally experience severe water scarcity for at least one month of the year [281]. Global water consumption has been increasing by 1% per year since the 1980s, which is projected to continue as the agriculture industry expands to meet global food demands [281]. On average, 780,000 people die each year from diseases associated with unsanitary water, an order of magnitude higher than conflict-related deaths. The removal of hazardous chemical species and biological pathogens from water is therefore a critical global issue to sustain development and provide humanity with the basic human right to clean drinking water. Powerful oxidising heterogeneous photoredox catalysts can degrade pollutants and kill bacteria without introducing chemical agents and require only sunlight for their operation, which is conveniently an abundant resource for many countries that are affected by poor water sanitation. The implementation of flow reactors facilitates purification process and provides easy manipulation of conditions to emulate the environment or to demonstrate scalability for industrial wastewater treatment.

**Figure 18:**
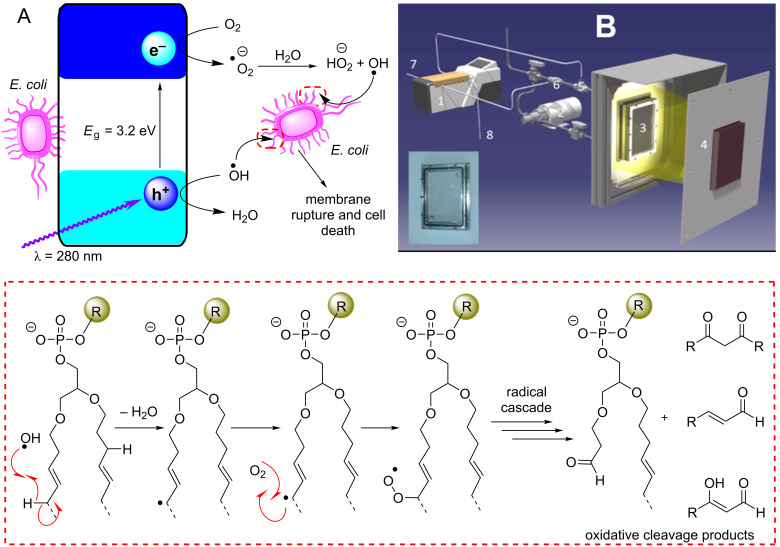
A) Photocatalytic bactericidal mechanism by ROS oxidative cleavage of membrane lipids (R = H, amino acid, membrane proteins) [282–283]. B) Graphical flow scheme of the cellulose fibre-supported TiO_2_ HPCat for the bactericidal photocatalytic removal of *E*. *coli* from water. (1) Peristaltic pump, (2) centrifugal pump, (3) photoreactor, (4) UV panel, (5) withdrawal valve, (6) feed valve, (7) effluent to be treated, and (8) treated effluent. Adapted with permission from [282]. Copyright 2017 American Chemical Society.

Goetz and co-workers studied the use of TiO_2_ bound by SiO_2_ to non-woven cellulosic fibres as a HPCat for the removal of *E. coli* from water under UV irradiation in continuous flow [282]. The use of continuous flow was critical for providing realistic comparisons to industrial wastewater treatment plant scales. The flow reactor system employed used a centrifugal pump to drive a closed reactor loop and ensure homogeneous mixing of the solution throughout the entire system. The input and output of the *E. coli* solution, before and after treatment, was driven by a multichannel peristaltic pump. The 2-dimensional immobilised TiO_2_ HPCat was placed in a flat-plate reactor, covered with a transparent PMMA sheet, and placed in front of an array of UVA LEDs. The effects of varying the photon flux and flow rate was studied, and both parameters were found to influence the number of the deactivated bacterial cells. At 35 W⋅m^−2^ of UVA light irradiation, an intensity consistent with solar irradiation, the HPCat system was calculated to have a treatment capacity of 15–30 × 10^5^ MPN⋅h^−1^⋅L^−1^ (MPN = most probable number of bacteria, calculated by the optical density at 600 nm). Due to the complex biomolecular structure of living cells, photocatalytic disinfection has many mechanisms and has been well studied and modelled elsewhere [283]. In general, the HPCat produces reactive oxygen species (ROS), such as singlet oxygen, hydroxyl radicals, and superoxide radicals. ROS react with unsaturated phospholipids within the cell membrane to generate peroxides and radical cascades that ultimately rupture the membrane structure and kill the cell (Figure 18). Vulnerable surface proteins and polysaccharides can also be deactivated by ROS, preventing cell function and leading to apoptosis or necrosis (programmed or involuntary cell death). The complimentary surface charges of the TiO_2_ HPCat and bacterial cells can favour surface adsorption, permitting direct oxidation and reduction processes with the membrane that may also be important processes during disinfection [283].

**Figure 19:**
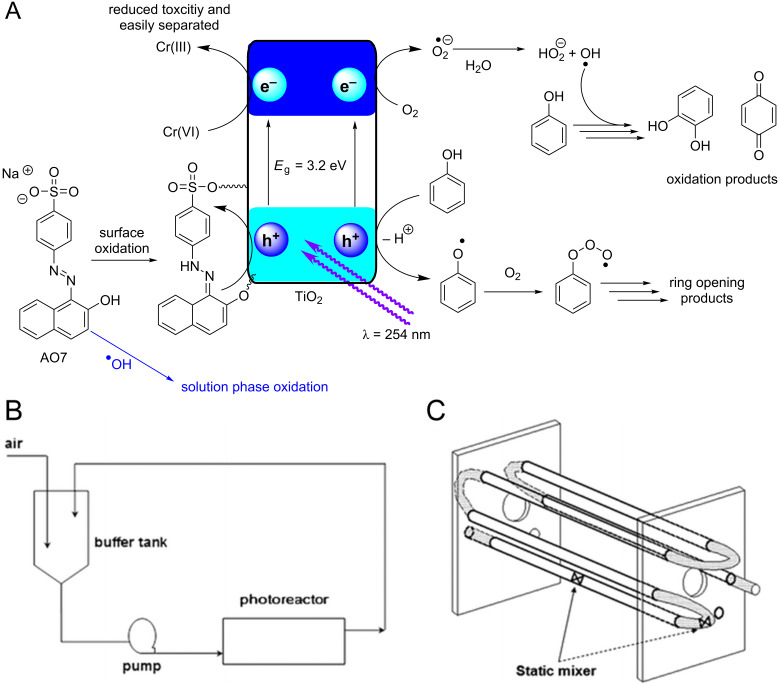
A) Suggested mechanisms for the aqueous pollutant degradation by TiO_2_ in a slurry flow reactor [284–287]. B) Flow schematic of the slurry reactor and C) more detailed schematic of the irradiated reactor with a static mixer in the dark silicone tubing (dashed arrow) or an irradiated quartz tube (solid arrow). Reprinted with permission from [214], Copyright 2011 The Royal Society of Chemistry.

The treatment of aqueous dyes from textile waste streams is problematic due to their large-scale production, solubility, and inefficient bioremediation [18]. The HPCat generation of ROS and direct photoredox processes can efficiently degrade many dyes to species that are easily removed or completely mineralised to CO_2_, water, and harmless anionic species. Lu and co-workers reported a continuous flow slurry reactor using commercial TiO_2_ (Degussa P25) for the remediation of a variety of aqueous pollutants, such as phenol, Cr(VI), and an organic dye (acid orange 7, AO7) as well as for the photoredox-catalysed reduction of *p*-nitrophenol to *p*-aminophenol [214]. The aim of the study was to see the effects of positioning a static mixer unit within the illuminated photoreactor or the dark silicon pipes, which connected the photoreactor chambers. This was to assess the contributions of the surface and bulk solution processes to the photocatalysis efficiency, inspired by the light and dark reactions of natural photosynthesis. Interestingly, the group found that in the cases where photocatalysis occurred by direct electron transfer between the HPCat and a strongly adsorbed substrate (e.g., Cr(VI) and AO7), the static mixer had no effect on the reaction. For reactions that occur primarily through the generation of ROS, which desorb and react primarily in the bulk solution, the static mixer gave significant rate enhancements of 20–90%. Of the systems that were enhanced, the position of the mixer had no effect on the rate, showing that the homogeneous dark reaction occurring in the bulk solution is limiting the efficiency of the reactor more so than the radical generation at the HPCat surface. They suggested that as the placement in the light or dark had no influence, the static mixers and photochemical reactors could be separated into individual components of a flow system, providing process intensification without complicated reactor designs, trying to incorporate large irradiation surface areas and static mixing in a single unit. The mechanism for select examples of photocatalytic pollutant degradation in water have been studied in detail and are represented in Figure 19, along with a schematic of the flow system used in the study [284–287].

Later in the same year, the group reported the same setup for the degradation of model water pollutants, but featuring five larger Kenics static mixer units in each of the irradiated quartz tubes for process intensification [216]. Terephthalic acid was used as a fluorescence probe sensor for hydroxyl radicals, revealing that the concentration of hydroxyl radicals in the bulk solution was enhanced by the static mixer units. They again observed that the photocatalytic degradation localised at the HPCat surface was not enhanced by the static mixing units, but the bulk solution phase degradation by the ROS was significantly enhanced, especially at lower flow rates [216].

**Figure 20:**
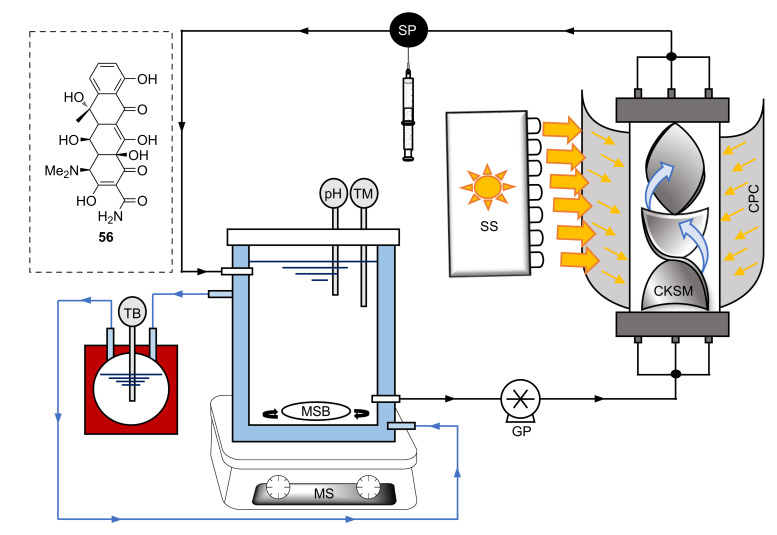
Schematic of the flow system used for the degradation of aqueous oxytetracycline (**56**) solutions [215]. MS = magnetic stirring plate, MSB = magnetic stir bar, GP = gear pump, CKSM = coated Kenics static mixer, CPC = compound parabolic collector, SS = Suntest XLS+ solar simulator, TB = thermostatic bath, pH = pH meter, TM = temperature meter, SP = sampling point.

Following this report, Vilar and co-workers recently reported the use of Kenics static mixers as a support material for TiO_2_ and Fe_2_O_3_ HPCats, applied by dip-coating and spray coating [215]. The group placed the coated static mixer units in a borosilicate column surrounded by a compound parabolic collector (CPC) solar concentrator and applied the reactor for the degradation of aqueous oxytetracycline (**56**, Figure 20). Antibiotics pollution in waste water is a serious issue due to concerns of bacteria developing resistance, but many waste streams and water treatment facilities are not effective in their removal through conventional bioremediation [288]. The group’s system was able to completely degrade **56** to dissolved organic carbons in under an hour with the Fe_2_O_3_ spray-coated static mixer, and in just over two hours with the TiO_2_-coated mixer in the presence of H_2_O_2_. The byproducts were further degraded to low-molecular-weight carboxylic acids and gaseous byproducts, removing approximately 60% of the initially dissolved organic carbon after 6 hours with both supported catalysts. The superior activity of the Fe_2_O_3_-coated static mixer was found to be partially due to a significant leaching of Fe ions into the solution, resulting in a significant contribution from homogeneous Fenton and photo-Fenton processes. The Fenton reaction is the production of hydroxyl radicals from H_2_O_2_ by Fe ions and can be catalytic under light irradiation or an applied voltage (vide infra) [289]. The amount of iron leached from the coating decreased over three cycles, and the group suggested that a fraction of the spray-coated HPCat was unstable and easily detached. Once removed by sequential reaction cycles, the Fe leaching dropped to undetectable amounts. They identified no leaching of TiO_2_ from the spray-coated static mixer. The static mixer support provided an efficient mixing of a laminar flow input, and its geometry enhanced the illumination of the immobilised HPCat by providing front and side irradiation from the CPC. The mixing was sufficient with only two coated static mixer units, and increasing to six did not significantly improve the rate of **56** degradation. The flow scheme and structure of **56** are displayed in Figure 20 [215].

**Scheme 27:**
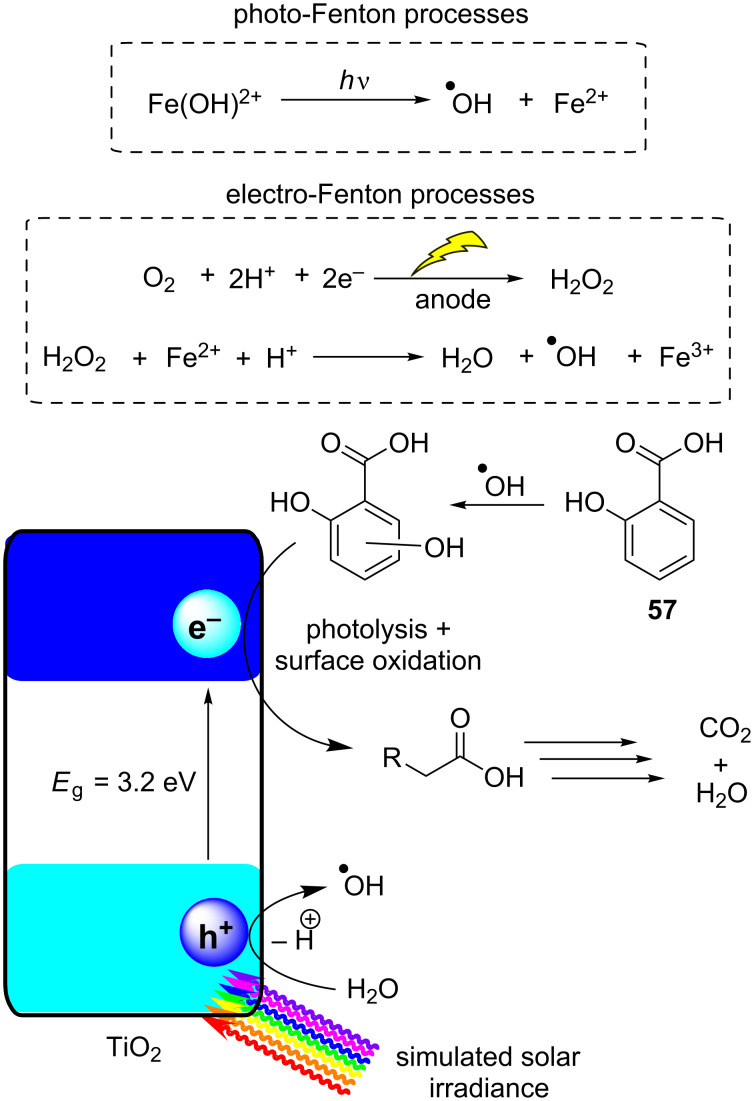
Degradation of a salicylic acid (**57**) solution by a coupled solar photoelectro-Fenton (SPEF) process and solar photocatalysis (SPC) [290].

Ruiz-Ruiz and co-workers studied the complete mineralisation of aqueous salicylic acid (**57**) by combing solar photoelectro-Fenton and solar heterogeneous photocatalysis in a single continuous flow process (Scheme 27) [290]. Salicylic acid (**57**) is commonly employed in cosmetic, pharmaceutical, and food formulations and has been detected in the wastewater of urban areas in many countries at concentrations of up to 50 μg⋅L^−1^ [291–293]. The flow system had a 3-litre capacity and featured a Pt/air diffusion (anode/cathode) electrochemical cell, a solar photoreactor, and a HPCat reactor filled with TiO_2_ immobilised on glass spheres. The individual reactors generated hydroxyl radicals through water electrolysis at the Pt anode, photocatalysis at the TiO_2_ surface, and via Fenton’s reaction between the added Fe^2+^ catalyst and H_2_O_2_, which was generated in situ by the cathode. The efficiency of the solar and electrochemical reactors individually and in combination, without the addition of Fe^2+^, led to the poor removal and mineralisation of **57**, which the authors proposed to be due to the lower oxidation power of the surface-generated hydroxyl radicals.

The electro-Fenton reaction in the absence of the solar reactors did efficiently degrade salicylic acid (**57**) to low-molecular-weight carboxylic acid byproducts. However, the carboxylic acid byproducts formed were resistant to further degradation. The optimal system was a combination of all three processes, as the carboxylic acid byproducts were susceptible to photolysis and further oxidation on the TiO_2_ HPCat surface. The system was able to achieve 87% mineralisation of a 165 mg⋅L^−1^ solution of salicylic acid (**57**) after 360 minutes of simultaneous water electrolysis, photolysis, and Fenton’s reactions.

#### 4.4 Photocatalytic air purification in flow

Air pollution contributed to approximately 5 million deaths globally in 2017 [294]. The anthropogenic emission of volatile organic compounds (VOCs), nitrous oxides (NO_x_), and other hazardous chemical species is the leading cause of acid rain, airborne particulate concentration, smog, and ozone formation in the troposphere [294]. These issues are particularly prevalent in developing countries with rapidly expanding industries and those still dependent on burning coal as an energy source. However, the sociodemographic pattern of ozone pollution shows that the highest ppb levels occur in more developed countries as the nitrous oxides and VOC pollutant precursors are correlated with industrialisation and economic development [294]. HPCats can efficiently remediate toxic gaseous pollutants, especially porous materials with high surface areas, which are much better suited to binding and reacting with gaseous substrates than a gas/liquid system. A heterogeneous solid/gas phase reaction also naturally lends itself to flow chemistry as the expected application of the HPCat would be at the end of a gas exhaust stream.

Herrmann and co-workers recently demonstrated the photocatalytic ability of TiO_2_ in the remediation of nitrous oxide pollutants in the air using a fixed bed flow reactor and UV irradiation [295]. The flow system featured 5 mass flow controller units and 3 mixing chambers, which allowed control over the flow rates and concentrations of nitrous oxides, water vapour, and compressed air. The group manipulated the conditions to simulate the atmosphere and found that the humidity and mixing ratios were the most important factors to consider, consistent with the involvement of the adsorbed water molecules on the TiO_2_ photocatalyst forming hydroxyl and superoxide species that then oxidise adsorbed NO and NO_x_ species. The study highlighted the careful choice of the system conditions to accurately simulate the concentrations of pollutants in the atmosphere. This was in order to make the collected data independent of the reactor dimensions so it could be accurately extrapolated to any simulated atmospheric conditions. Contrary to the heterogeneous solid/solution photocatalysis discussed earlier in this review, gas phase heterogeneous photocatalysis processes are almost exclusively surface-based phenomena that occur in elementary steps between adsorbed species.

**Figure 21:**
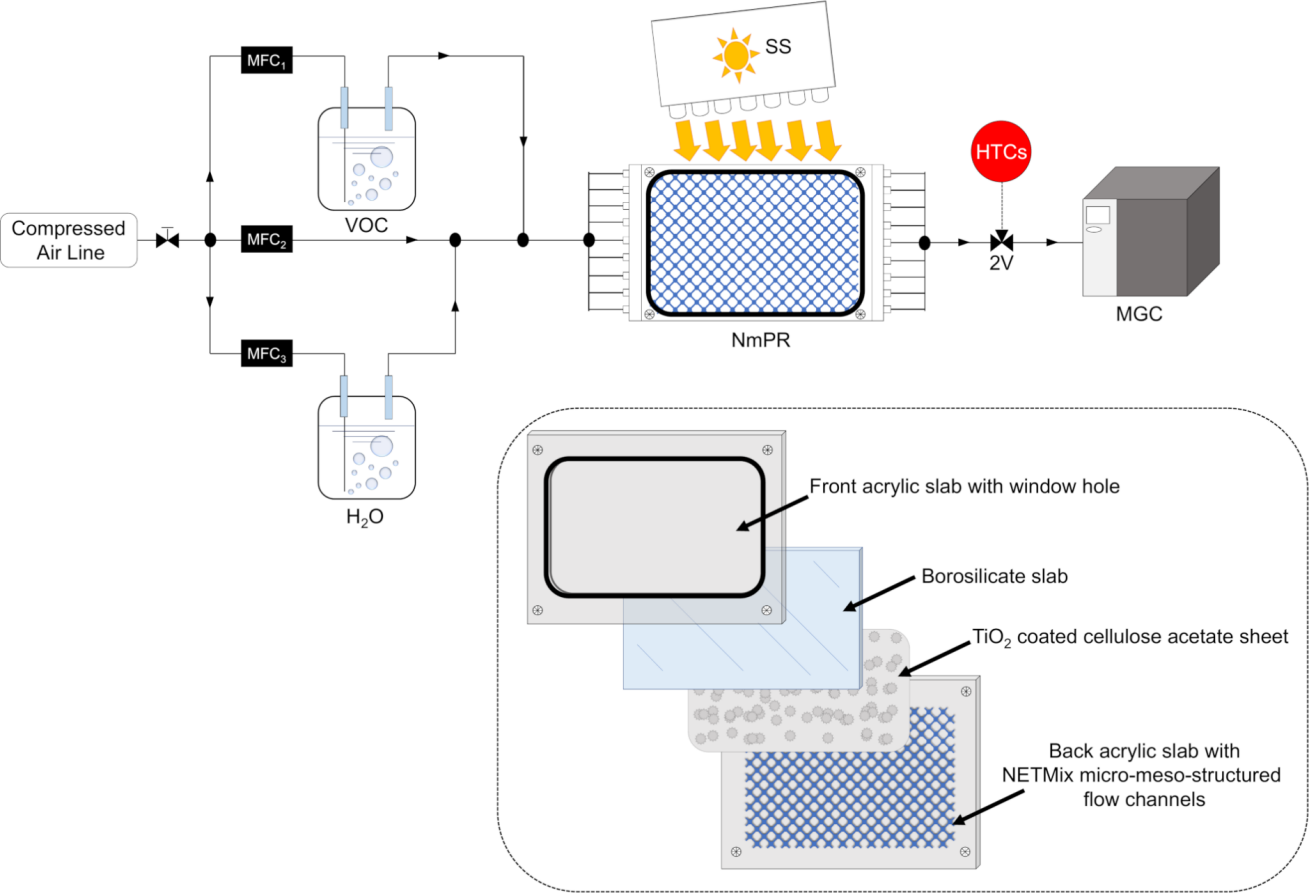
A) Schematic flow diagram using the TiO_2_-coated NETmix microfluidic device for an efficient mass transport during the degradation of gaseous volatile organic compounds [296]. MFC = mass flow controller, VOC = volatile organic compound reservoir Woulff bottle, H_2_O = water reservoir Woulff bottle, MGC = master gas chromatograph, 2V = two-way valve, NmPR = NETmix photoreactor, SS = Suntest solar simulator, HTCs = humidity, temperature, and CO_2_ sensor. B) Graphical representation of the component layers that form the NETmix microfluidic device fabricated by Vilar and co-workers [296].

The efficient oxidative degradation of gaseous *n*-decane was achieved in another report by Vilar and co-workers in which a cellulose acetate sheet was coated with a thin film of TiO_2_ as the HPCat (Figure 21A) [296]. The HPCat sheet was placed between a borosilicate window slab and a micro-meso-structured photoreactor. This comprised a slab of acrylic that had flow channels mechanically engraved into the material, forming a two-dimensional coated reactor with microchannels connecting to millimetre-scale “meso-chambers” in a diagonal “crisscross” pattern (Figure 21B). The design was inspired by the NETmix technology, developed by Dias and co-workers [297], and acts as an efficient static mixer unit for solution and gas flow streams.

The reactor was able to efficiently oxidise gaseous *n*-decane in the presence of water vapour to aldehyde and ketone byproducts, with a consistent performance over 72 hours of continuous irradiation. The authors proposed that a longer retention time within the reactor would sequentially oxidise and degrade the byproducts to smaller-carbon-chain intermediates and eventually lead to a total mineralisation. It was reported that 75 mg of TiO_2_ was the optimal loading, as higher loadings were observed to reduce the efficiency. This was due to the layer thickness of TiO_2_, increasing and reducing the surface area as the surface-immobilised layers became inaccessible to the gas flow. Additionally, the authors proposed the higher TiO_2_ layer depth contributed to the “back-irradiation” phenomenon in which the photogenerated charge carriers are further from the HPCat/gas interface, and charge recombination becomes significant. An increasing flow rate facilitated mixing in the NETmix-type static mixer channel design and enhanced the photodegradation efficiency up to a point where the residence time of the gaseous reagents in the reactor became the limiting factor.

## Conclusion

Heterogeneous photocatalysis and flow chemistry form a synergistic pairing that has the potential to become the “power couple” of photochemistry. The typical disadvantages of HPCats are largely mitigated through continuous flow reactors’ improved mass transport and irradiation. In this review, we have covered the different types of HPCats and how they function as photocatalysts. Enhancing charge transport and separation in semiconductor HPCats is a key factor to improve photocatalytic activity, which can be achieved through materials design and band gap engineering. The physical nature of metal oxide surfaces and the substrate’s adsorption have also been described, which could influence the performance of a HPCat regardless of its charge transport efficiency. The different types of flow reactors and systems that HPCats can be applied in, as well as their advantages and disadvantages, were discussed with the objective of guiding the reader toward the best reactor to suit their intended system.

Finally, we reviewed the different photoredox catalysis and energy transfer catalysis synthetic transformations that have been achieved with HPCats in flow reactors. The photocatalytic removal of pollutants from waste water and air were discussed with an emphasis on the flow systems and photoreactor designs that utilise static mixing technology, which could be effective if applied in organic synthesis. A recurring trend throughout the examples of heterogeneous photocatalysis discussed was that flow chemistry can significantly reduce the reaction times and increase the process productivity, often by orders of magnitude compared to analogous batch reactor protocols.

We suggest that the development of HPCats in continuous flow will lead to a new paradigm for sustainable photochemical syntheses. However, this is highly dependent upon advancements in several areas of scientific research, as represented in Figure 1B. We believe that interdisciplinary collaborations will provide rational design principles for more efficient HPCat materials and flow reactors, which will collectively exceed the capabilities of rare transition metal complex photocatalysts and provide more sustainable, large-scale photosynthetic methodologies.
